# Supplementary material for: Improving routine childhood immunisation outcomes in low-income and middle-income countries: an evidence gap map
Source: BMJ Open. 2022 Nov 10;12(11):e058258. doi: 10.1136/bmjopen-2021-058258 (PMC9660714; doi:10.1136/bmjopen-2021-058258)

Supplemental material: Improving routine childhood immunisation outcomes in low- and middle-income countries: an evidence gap map

Appendix A: Intervention-outcome framework and definitions **3**

Interventions 3

Outcomes 9

Cross-cutting themes 13

Appendix B: Detailed methods **14**

B.1 Database searches 14

1. Ovid MEDLINE(R) and Epub Ahead of Print, In-Process & Other Non-Indexed Citations, Daily and Versions(R) <1946 to May 15, 2019> Searched 16th May 2019 14

2. Embase Classic+Embase (Ovid) <1947 to 2019 May 15> Searched 16th May 2019 16

3. CAB Global Health (Ovid) <1910 to 2019 Week 19> Searched 17th May 2019 18

4. Cochrane Library – searched 17th May 2019 20

5. PsycINFO (Ovid) <1806 to May Week 2 2019> Searched 18th May 2019 22

6. CINAHL (Ebsco) – Searched 18th May 2019 23

7. Ebsco Discovery – Searched 19th May 2019 – limited to: Academic Search Complete (2411); Africa-Wide (2170); Ideas-Repec (123); Econlit (67); World Bank e-library (23) 28

8. Scopus – Searched 19th May 2019 32

9. Popline – Searched 19th May 2019 33

10. WHO Global Health Index – Searched 20th May 2019 34

11. Epistemonikos – Searched 20th May 2019 34

12. Pascal-Francis (Updated to 2015) – Searched 20th May 2019 35

B.2 Grey literature searches 36

B.3 Data extraction template 37

B.4 SR critical appraisal tool 39

Section A: *Methods used to identify, include and critically appraise studies* 39

Section B: *Methods used to analyse the findings* 44

Section C: *Overall assessment of the reliability of the review* 49

Appendix C: References – Included studies **50**

Impact evaluations 50

Systematic reviews 70

High confidence 70

Medium confidence 70

Low confidence 71

Appendix D: Additional figures 75

Figure D.1: Impact evaluations by specific intervention category (multi-component separated) . 76

Figure D.2: SR critical appraisals – methods used to identify, include, and critically appraise studies 77

Figure D.3: SR critical appraisals – analysis methods 78

Appendix A: Intervention-outcome framework and definitions

Interventions

Intervention			Definition/guidance
A. Caregiver-oriented	AA. Information & education	AA1. Sustained sensitization and education campaigns	Sustained interventions (i.e., those that are *not* designed with a fixed end date in mind) that provide targeted caregivers with information about immunisation and its importance, the vaccination schedule, or where and how to access immunisation services. For example, village health and nutrition days (VHNDs) in India in which health education and counselling services are provided to pregnant women and mothers of young children on a regular basis.
		AA2. One-time sensitization and education campaigns	One-off interventions (i.e., those designed with a fixed end date in mind) that provide targeted caregivers with information about immunisation and its importance, the vaccination schedule, or where and how to access immunisation services.
		AA3. Public information campaigns	Mass media campaigns through newspapers, radio and TV which provide caregivers with information about immunisation and its importance, the vaccination schedule, or where and how to access immunisation services. Because of the nature of communication mediums they cannot be targeted to a specific audience.
	AB. Incentives & motivation	AB1. Material/ monetary incentives for caregivers	Interventions that incentivise caregivers to vaccinate through items with monetary value. This could be cash transfers or material goods like food or home goods.
		AB2. Non-material incentives for caregivers	Interventions that seek to motivate caregivers to vaccinate through non-material incentives like social recognition. Unlike reminder messages, interventions in this category should seek to *create* or *strengthen* a desire to vaccinate, rather than activating a standing intention to vaccinate.
		AB3. Automated voice messages to caregivers	Use of automatically-generated voice messages (usually delivered to a mobile phone) that remind caregivers about upcoming vaccinations, provide them information on place and time of vaccination and encourage them to vaccinate. While these messages may contain some motivational component (e.g., stressing the importance of vaccination in addition to reminding caregivers about an upcoming appointment), voice messages should be categorised only here and not also under “non-material incentives”, unless they rely on a substantive motivational factor like social recognition.
		AB4. Written or pictorial messages (SMS, stickers, flyers etc.) to caregivers	Use of written messages/pictorial that remind caregivers about upcoming vaccinations, provide them information on place and time of vaccination and encourage them to vaccinate. While these messages may contain some motivational component (e.g., stressing the importance of vaccination in addition to reminding caregivers about an upcoming appointment), messages should be categorised only here and not also under “non-material incentives”, unless they rely on a substantive motivational factor like social recognition.

Intervention			Definition/guidance
		AB5. Changes to health system user fees	Any change to the monetary costs to users for accessing the health system. This can include introduction or elimination of fees at the point of service, or pre-payment or insurance schemes.

Intervention			Definition/guidance
B. Health system-oriented	BA. Education & training	BA1. Formal health worker training and education	Programmes that train or educate formal health workers (FHWs). FHWs are typically vaccinators (and they tend to provide/prescribe medication or administer tests such as recording blood glucose level, etc.). (The only likely exception to this would be oral polio vaccination (OPV), especially supplementary polio campaigns, where CHWs or community volunteers may be enlisted to administer the vaccination.)
		BA2. Community health worker training and education	Programmes that train or educate community health workers (CHWs). CHWs are defined as "paraprofessionals or lay individuals with an in-depth understanding of the community culture and language, have received standardised job-related training of a shorter duration than health professionals, and their primary goal is to provide culturally appropriate health services to the community."[1]
	BB. Planning, implementation, monitoring	BB1. Formal health worker involvement in planning & monitoring	Interventions that give FHWs substantive roles in creating plans/strategies to deliver vaccination services and/or monitor vaccination coverage in the community.
		BB2. Community health worker involvement in planning & monitoring	Interventions that give CHWs substantive roles in creating plans/strategies to deliver vaccination services and/or monitor vaccination coverage in the community.
		BB3. Paper-based tracking	Paper-based systems (e.g., logbooks) used by health workers to keep track of children in the community who are due for upcoming vaccinations or have not received scheduled vaccinations. Note that if a study merely mentions the existence of logbooks, that is not sufficient to code it as this intervention. The logbooks must be specifically used for tracking upcoming and missed vaccinations.
		BB4. Promoting outreach to vaccine-hesitant groups	Outreach to groups that, because of religious, cultural, or other reasons, are suspicious of vaccination or have specific fears about it (e.g., that vaccinations cause infertility or spread disease).
		BB5. Outreach to vulnerable populations (hard-to-reach, SES, caste, etc.)	Outreach to groups that are vulnerable in a way that affects their access to vaccination services. They may be in hard-to-reach geographical areas, have low socioeconomic status (including wealth and education), or be from groups marginalised based on caste, ethnicity, etc. This also includes interventions that set up temporary and mobile clinics to deliver vaccines, to make vaccination services more accessible.
		BB6. Outreach to migrant populations	Outreach to populations who have migrated temporarily or seasonally because of cultural or employment reasons.

[1] From Olaniran, A., Smith, H., Unkels, R., Bar-Zeev, S., & van den Broek, N. (2017). Who is a community health worker? – A systematic review of definitions. *Global Health Action*, *10*(1), 1272223. https://doi.org/10.1080/16549716.2017.1272223.

Intervention			Definition/guidance
		BB7. Home visits	Use of visits to caregivers' homes by health workers. This includes both visits to encourage caregivers to vaccinate their children, and visits to deliver vaccines, provided they are *targeted* visits to specific households for routine immunization. General door-to-door campaigns to deliver specific vaccines go under BC1.
		BB8. Campaigns to vaccinate refugee populations	Interventions that make a special effort to vaccinate populations which have been displaced temporarily or permanently because of conflict, war or famine.
	BC. Supplementary Immunisation Activities	BC1. National/sub-national immunisation days	Supplementary immunization activities (SIA) are mass immunization campaigns which complement routine immunization activities whereby a vaccine is taken simultaneously to many residents of a community within a defined short space of time. They have generally been conducted for polio and measles. They may be called national or sub-national immunization days. They may happen through booth days or door to door vaccination campaigns. On a booth day a large number of fixed site booths are set up throughout the target area for children to be brought to receive a specific vaccine like polio. In door to door campaigns vaccination teams go door to door to every house, checking each child under five to see if they have received the specific vaccine, and if they have not that vaccination is provided.
	BD. Incentives & motivation	BD1. Material/monetary incentives for health workers	Interventions that incentivise formal or community health workers to deliver vaccination services through items with monetary value. This could be cash transfers or material goods like food or home goods.
		BD2. Non-material incentives for health workers	Interventions that use non-material incentives like social recognition to incentivise formal or community health workers to deliver vaccination services.
		BD3. Automated voice messages to health workers	Use of automatically-generated voice messages (usually delivered to a mobile phone) that remind health workers about upcoming vaccinations for community members, provide them information to help plan their work, and encourage them to conduct outreach to those community members.
		BD4. Written or pictorial messages (SMS, stickers, flyers etc.) to health workers	Use of written/pictorial messages that remind health workers about upcoming vaccinations for community members, provide them information to help plan their work, and encourage them to conduct outreach to those community members.
		BD5. Pay-for-performance schemes	Schemes whereby health centres or districts receive funding based on their performance in delivering health services (e.g., they receive a given amount of funding for each child vaccinated or each antenatal care visit completed). Also known as "results-based financing".

Intervention			Definition/guidance
	BE. Infrastructure	BE1. Building & upgrading health clinics	Projects that build new permanent health clinics, or provide physical upgrades to existing clinics.
		BE2. Cold chain infrastructure improvements	Interventions that improve the ability of health systems to maintain vaccine cold chains.
	BF. Health system governance, policies and financing	BF1. Health system strategic planning	Initiatives at the national or sub-national level to develop plans and governance structures designed to improve vaccination services. This also includes interventions that improve the human resource availability, strategies, policies and plans in existing health governance and delivery structures that may or may not be directly related to immunization services.
		BF2. Vaccination guidelines	Changes to official national or sub-national guidelines about when and how vaccinations should be administered. This includes studies comparing two different approaches to administering vaccines (e.g., one measles dose vs. two), which could be made into guidelines.
		BF3. Changes to broader governance systems (beyond health systems)	Interventions that modify general governance systems not directly related to health. An example would be a policy dictating a certain level of representation for women in local or national governing bodies.
		BF4. Health system financing	Interventions that increase the national or sub-national financing of health or specifically for vaccination in absolute terms or as a proportion of GDP. This also includes results- or performance-based financing.
	BG. Technology & mHealth	BG1. New HMIS/Dashboard systems (incl. improved data collection)	New digital tools and systems designed to improve health system capacity to monitor and deliver vaccination services. This includes apps for mobile phones or tablets, as well as desktop-based software and setting up online dashboards. A common type of intervention in this category is giving health workers tablets with an app that allows them to register vaccination information (and for other health services) for community members, track their vaccination schedules and provide them readily-accessible information about vaccination.
		BG2. Capacity building (e.g., training) for existing systems	Initiatives to train people working in the health system (including frontline health workers and administrative personnel) to improve their ability to use existing digital tools and systems more effectively.
C. Other community member-oriented	CA. Other community member-oriented	CA1. Faith-based outreach/outreach using local leaders	Interventions that enlist influential community members (often religious or other traditional leaders) to promote vaccination in the community.
D. Community-level	DA. Communication & dialogue	DA1. Collaborating with whole community	Interventions that involve or plan to involve ALL community members beyond health workers in various aspects of the intervention, such as developing plans and solutions to improve immunization outcomes in the community.

Intervention			Definition/guidance
		DA2. Collaborating with selected community groups and networks	Interventions that involve *selected* groups or networks of community members beyond health workers (other than the traditional or religious leaders who fall under CA1) in developing plans and solutions to improve immunization outcomes in the community. This includes interventions focusing on mother's groups, father clubs, self-help groups, etc.
	DB. Tracking & registering	DB1. Community tracking and registering	Interventions that involve community members beyond health workers (e.g., caregivers or any other community members other than the traditional leaders who fall under CA1) in registering children with the health system so their vaccination status can be tracked, and/or tracking which children are due for vaccinations.
E. Policies and institutions	EA. Education policy and infrastructure	EA1. Education policy and infrastructure	Policy interventions that affect education levels of people in a country or region. An example would be an intervention that makes education mandatory for a particular population where it had previously been optional.
	EB. Non-health/ education infrastructure	EB1. Non-health/ education infrastructure (e.g., electrification)	General improvements in physical infrastructure beyond the health system. This may include electrification, roads, sanitation improvements, etc.

Outcomes

		Outcome	Definition/guidance
H. Behavioural, social and practical barriers faced by caregivers and communities	HAA. Thinking and feeling (attitudes, confidence)	HAA1. Knowledge about immunisation	Caregivers' knowledge about immunisation in general (i.e., its purpose and role in preventing disease)
		HAA2. Attitudes about immunisation	Caregivers' attitudes towards immunisation in general (i.e., whether they view it favourably or unfavourably or have high or low confidence in its efficacy)
		HAA3. Attitudes about health providers	Caregivers' attitudes about health providers in general (i.e., whether they generally trust health providers to provide high-quality and appropriate care)
	HAB. Social processes	HAB1. Community norms	Community-level attitudes and beliefs about immunisation, including whether there is social pressure to vaccinate or not vaccinate. This can be measured either objectively through aggregating community-level responses or subjectively by soliciting individual community members' beliefs about the norms in their community. This includes attitudes and beliefs about immunization of key influencers in the community like traditional or religious leaders.
		HAB2. Household norms & decision-making	Norms and practices determining who in a household (e.g., mother, father, mother-in-law) provides input to decisions about whether to vaccinate, and how much decision-making power individual household members have. This also covers attitudes towards immunisation of household members other than the primary caregiver.
	HAC. Readiness to vaccinate	HAC1. Readiness to vaccinate	Caregivers' motivation, intention and plan to vaccinate their children. Note this is more specific than general attitudes towards immunisation covered under HAA2
		HAC2. Reasons for not vaccinating	Caregivers' stated reasons for not vaccinating children. This may include factors such as convenience (which would also be coded under "perceived convenience of vaccination"), but only use this code if the factors mentioned are specifically framed as reasons for not vaccinating. Also, only use this code when the study measures *effects* of the intervention on this outcome. If the study gathers data on reasons for not vaccinating but does not provide an effect size for this as an outcome, use the cross-cutting theme but not this outcome code.

		HAD. Practical factors	HAD1. Awareness of place, time, schedule for vaccination	Caregivers' knowledge about when and where they should go for vaccinations.
			HAD2. Actual cost of vaccinating	Actual cost of vaccinating the child, including vaccine cost, transportation cost, loss of wage/income due to missed work, and providing gifts/unofficial payments to the health providers
			HAD3. Perceived convenience of vaccination	Subjective measures (i.e., caregivers' beliefs) of the convenience of taking the child for vaccination, such as opportunity costs of vaccinating a child (e.g., not able to care for a younger child), long lines at health clinic, and inconvenient day/time of vaccination.
			HAD4. Experience and satisfaction with health services	The actual experience of health services in the last visit such as duration of waiting time, availability of vaccine or vaccinator, and behaviour of the health staff (respect, rudeness). This also includes level of satisfaction with the health services, professionals and facilities.
			HAD5. Vaccination health card availability/ retention	Measures of whether caregivers possess vaccination health cards provided by the health system, and/or whether caregivers can show the vaccination health card.
			HAD6. Perception of vaccination side effects	Caregivers' perceptions of the likelihood and severity of side effects from vaccination, and their knowledge of how to recognise and treat normal side effects.
I. Delivery of vaccination services	IA. Health workforce	IAA. Comm. health workers	IAA1. Community HW motivation, capacity & performance	Any measure of CHWs' capacity to deliver quality and timely vaccination services.
			IAA2. Supply of CHWs	The total availability of CHW services in the community, taking into account both the number of CHWs and the time they have available.
		IAB. Vaccinators	IAB1. Formal HW supply	The total availability of FHW services in the community, taking into account both the number of FHWs and the time they have available.
			IAB2. Availability of HWs at vacc. point of service	Whether vaccinators are present at vaccination point of service (e.g., health clinic) when vaccination services are supposed to be offered. This includes measures of health worker "absenteeism". This is different from perception or experience of health workers from caregivers' point of view. The source of information for this outcome can be administrative data or survey of health facilities and staff.
			IAB3. Formal HW motivation, capacity & performance	Any measure of FHWs' capacity to deliver quality and timely vaccination services, or of their performance in doing so.
		IAC. Administrators	IAC1. Admin staffing	The number of staff in administrative posts in the health system (i.e., those not directly involved in health service provision).
			IAC2. Capacity of health admin. responsible for vaccination	The knowledge, skills, and motivation of staff in administrative posts in the health system, including leadership positions.
	IBA. Health information systems		IBA1. Immunisation data collection (quality, completeness)	The health system's capacity for and success in collecting data about vaccination coverage and service quality for regular monitoring and accountability.
			IBA2. Defaulter tracing	The health system's capacity for and success in identifying vaccination "defaulters" (i.e., those whose children receive early vaccine doses but do not return for subsequent vaccinations).
			IBA3. Supply chain management	The health system's capacity to monitor the supply of vaccines at points of service, ensure reliable supply chains, and avoid supply "bottlenecks".

		IBA4. Immunisation data availability/ transparency	How easy it is for stakeholders within and beyond the health system to access data about vaccination service quality, coverage and timeliness.
	ICA. Vaccine availability	ICA1. Stockouts	The frequency and duration of incidents when vaccines are out of stock at points of service, or when vaccines are in stock but cannot be administered to children who are brought in (e.g., because health workers are instructed not to open a new vial if there are not enough children to receive all doses in the vial, meaning that some of the vaccine would go to waste). Source of this information is administrative data and/or survey of health facilities and staff
		ICA2. Quality of cold chain infrastructure	The availability, quality and upkeep of physical equipment and place for cold chain storage of vaccines
	IDA. Resources	IDA1. National or sub-national vaccine financing	Change in financial resources for national or sub-national vaccination programmes, policies or strategies.

JAA. Vaccination coverage	JAA01. Full routine immunization for children	Binary measure of whether or not children have received all routine vaccinations for the relevant country or region.
	JAA02. BCG	Binary measure of whether or not children have received the BCG vaccine. This may be measured by checking whether children have a BCG vaccination scar.
	JAA03. DPT1	Binary measure of whether children have received first dose of the DPT/penta vaccine. If study does not specify which doses were included in the outcome measures (i.e., the outcome is just "DPT/pentavalent vaccination" then apply this code unless it is clear they are talking about full DPT/penta vaccination, in which case code it as JAA05: DPT3.
	JAA04. DPT2	Binary measure of whether or not children have received the second dose of the DPT or pentavalent vaccine
	JAA05. DPT3	Binary measure of whether or not children have received the third dose of the DPT or pentavalent vaccine. If the study does not specifically say "DPT3" (or "pentavalent 3"), but refers to "complete DPT/penta vaccination", then use this code.
	JAA06. OPV0	Binary measure of whether children have received 1st dose of oral polio vaccine (recommended for administration at birth).
	JAA07. OPV1	Binary measure of whether children have received 2nd dose of the oral polio vaccine (recommended for administration at 6 weeks).
	JAA08. OPV2	Binary measure of whether children have received the 3rd dose of the oral polio vaccine (recommended for administration at 10 weeks).
	JAA09. OPV3	Binary measure of whether children have received the 4th and final dose of the oral polio vaccine (recommended for administration at 14 weeks).
	JAA10. IPV	Binary measure of whether children have received inactivated polio vaccine, given as injection. Countries differ in their guidelines/practices regarding IPV, so, please note the number of doses and age(s) when administered.
	JAA11. Measles	Binary measure of whether or not children have received the measles vaccine
	JAA12. No vs. partial routine immunization	Proportion of children who receive at least one vaccination versus those who are completely unvaccinated.
	JAA13. Vacc. timeliness	Proportion of vaccinations delivered on time according to the recommended schedule, vs those that are delivered late.
	JAA14. Drop out rate for multi-dose vaccines	Proportion of children who fail to receive the complete course of a multi-dose vaccine (DPT/penta, OPV, or in some cases measles) after receiving the first dose.
	JAA15. Vaccination coverage (unspecified)	Use this code if the evaluation or SR refers to impacts on routine vaccination coverage for children, but without specifying which vaccines.
KAA. Health outcomes	KAA1. Childhood morbidity	Incidence of vaccine-preventable diseases or symptoms associated with those diseases (e.g., diarrhoea) among children under 5.
	KAA2. Neonatal/Infant/ Child Mortality	Incidence of mortality among children below five years from all causes. Neonatal mortality refers to death of a live-born baby within the first 28 days of life. *Infant mortality* is the death of young children under the age of 1. Child mortality, refers to the mortality of children under the age of five. This category includes all-cause mortality.

Cross-cutting themes

Theme	Definition/guidance
Hard-to-reach	Code as "yes" if the study performs subgroup analysis for hard-to-reach populations, or if the intervention specifically targets a population that the authors define as hard-to-reach. See the guidance for gender/equity coding in the protocol for further information on what constitutes subgroup analysis.
Sex	Code as "yes" if the study performs subgroup analysis by sex of the children, or if the intervention specifically targets children of one sex. See the guidance for gender/equity coding in the protocol for further information on what constitutes subgroup analysis.
SES	Code as "yes" if the study performs subgroup analysis by socioeconomic status, or if the intervention specifically targets households below the locally-defined poverty line. See the guidance for gender/equity coding in the protocol for further information on what constitutes subgroup analysis.
Maternal education	Code as "yes" if the study performs subgroup analysis by maternal education, or if the intervention specifically targets mothers with a given level of education. See the guidance for gender/equity coding in the protocol for further information on what constitutes subgroup analysis.
Cost information	Code as "yes" if the study presents data on the costs of the intervention. This may be a detailed cost-effectiveness or cost-benefit analysis, but that is not necessary.
Information sources	Code as "yes" if the study includes data about or discusses different sources through which participants receive information about vaccination, or if the study analyses impact of exposure to different information sources on various immunization outcomes. Code as "no" if only one type of information source is discussed.
Adverse effects	Code as "yes" if the study includes data about or discusses potential adverse effects on participants as a result of the intervention.
Multi-component	Code as "yes" if the study evaluates an intervention that is multi-component.

Appendix B: Detailed methods

This EGM followed the 3ie methodology and process for evidence gap maps (Snilstveit et al. 2017; 2016).

B.1 Database searches

1. **Ovid MEDLINE(R) and Epub Ahead of Print, In-Process & Other Non-Indexed Citations, Daily and Versions(R) <1946 to May 15, 2019> Searched 16th May 2019**

1 (immuniz* or immunis* or vaccin* or inoculat* or innoculat* or immunotherap* or prophyla*).ti,ab,kw. (672732)
2 immunization/ or immunization, passive/ or immunization schedule/ or immunization, secondary/ or immunotherapy, active/ or vaccination/ or Immunization Programs/ or mass vaccination/ (158163)
3 Tuberculosis Vaccines/ or BCG Vaccine/ or Diphtheria-Tetanus Vaccine/ or Meningococcal Vaccines/ or Pertussis Vaccine/ or Diphtheria-Tetanus-acellular Pertussis Vaccines/ or Diphtheria-Tetanus-Pertussis Vaccine/ or Diphtheria-Tetanus Vaccine/ or Measles Vaccine/ or Mumps Vaccine/ or Rubella Vaccine/ or Measles-Mumps-Rubella Vaccine/ or Poliovirus Vaccines/ or Poliovirus Vaccine, Inactivated/ or Poliovirus Vaccine, Oral/ or Japanese Encephalitis Vaccines/ or Rotavirus Vaccine/ (49639)
4 or/1-3 (720583)
5 developing countries.sh,kf. (83271)
6 (Africa or Asia or Caribbean or West Indies or South America or Latin America or Central America).ti,ab,kw. (200981)
7 Africa/ or Asia/ or Caribbean/ or West Indies/ or South America/ or Latin America/ or Central America/ (73389)
8 (Africa or Central America or South America or Caribbean or Central Asia or Afghanistan or Albania or Algeria or Angola or Argentina or Armenia or Armenian or Azerbaijan or Bangladesh or Benin or Byelarus or Byelorussian or Belarus or Belorussian or Belorussia or Belize or Bhutan or Bolivia or Bosnia or Herzegovina or Hercegovina or Botswana or Brazil or Bulgaria or Burkina Faso or Burkina Fasso or Upper Volta or Burundi or Urundi or Cambodia or Khmer Republic or Kampuchea or Cameroon or Cameroons or Cameron or Camerons or Cape Verde or Cabo Verde or Central African Republic or Chad or Tchad or China or Colombia or Comoros or Comoro Islands or Comores or Mayotte or Congo or Zaire or Costa Rica or Cote d'Ivoire or Ivory Coast or Cuba or Djibouti or French Somaliland or Dominica or Dominican Republic or East Timor or East Timur or Timor Leste or Ecuador or Egypt or United Arab Republic or El Salvador or Eritrea or Ethiopia or Fiji or Gabon or Gabonese Republic or Gambia or Gaza or Georgia Republic or Georgian Republic or Ghana or Grenada or Guatemala or Guinea or Guiana or Guyana or Haiti or Honduras or India or Maldives or Indonesia or Iran or Iraq or Jamaica or Jordan or Kazakhstan or Kazakh or Kenya or Kiribati or Korea or Kosovo or Kyrgyzstan or Kirghizia or Kyrgyz Republic or Kirghiz or Kirgizstan or Lao PDR or Laos or Lebanon or Lesotho or Basutoland or Liberia or Libya or Macedonia or Madagascar or Malagasy Republic or Malaysia or Malaya or Malay or Sabah or Sarawak or Malawi or Mali or Marshall Islands or Mauritania or Mauritius or Agalega Islands or Mexico or Micronesia or Middle East or Moldova or Moldovia or Moldovian or Mongolia or Montenegro or Morocco or Ifni or Mozambique or Myanmar or Myanma or Burma or Namibia or Nepal or Netherlands Antilles or Nicaragua or Niger or Nigeria or Muscat or Pakistan or Palau or Palestine or Panama or Paraguay or Peru or Philippines or Philipines or Phillipines or Phillippines or Papua New Guinea or Romania or Rumania or Roumania or Rwanda or Ruanda or Saint Lucia or St Lucia or Saint Vincent or St Vincent or Grenadines or Samoa or Samoan Islands or Navigator Island or Navigator Islands or Sao Tome or Senegal or Serbia or Montenegro or Seychelles or Sierra Leone or Sri Lanka or Solomon Islands or Somalia or Sudan or Suriname or Surinam or Swaziland or Eswatini or South Africa or Syria or Tajikistan or Tadzhikistan or Tadjikistan or Tadzhik or Tanzania or Thailand or Togo or Togolese Republic or Tonga or

Tunisia or Turkey or Turkmenistan or Turkmen or Uganda or Ukraine or Uzbekistan or Uzbek or Vanuatu or New Hebrides or Venezuela or Vietnam or Viet Nam or West Bank or Yemen or Zambia or Zimbabwe or Rhodesia).ti,ab,kw,sh. (1387951)

9 ((developing or less* developed or least developed or under developed or underdeveloped or middle income or low* income or underserved or under served or deprived or poor* or resource limited or resource constrained) adj (countr* or nation? or population? or world or state*)).ti,ab,kw. (91955)

10 ((developing or less* developed or least developed or under developed or underdeveloped or middle income or low* income or resource limited or resource constrained) adj (economy or economies)).ti,ab,kw. (504)

11 (low* adj (gdp or gnp or gross domestic or gross national)).ti,ab,kw. (234)

12 (low adj3 middle adj3 countr*).ti,ab,kw. (12819)

13 (lmic or lmics or third world or lami countr*).ti,ab,kw. (6486)

14 (transitional countr* or emerging econom* or global south).ti,ab,kw. (873)

15 or/5-14 (1487811)

16 4 and 15 (80257)

17 Parents/ or Fathers/ or Mothers/ or Grandparents/ or Caregivers/ or Single Parent/ or Pregnant Women/ or Child, Preschool/ or Infant/ or Infant, Newborn/ or Infant, Low Birth Weight/ or Infant, Small for Gestational Age/ or Infant, Very Low Birth Weight/ or Infant, Extremely Low Birth Weight/ or Infant, Postmature/ or Infant, Premature/ or Infant, Extremely Premature/ (1597959)

18 (child* or infant* or newborn* or neonat* or neo nat* or prenatal or pre natal or ante natal or antenatal or baby or babies or toddler* or preschool* or parent* or mother* or father* or maternal or paternal).ti,ab,kw. (2284298)

19 or/17-18 (2893768)

20 16 and 19 (28515)

21 (random* or experiment* or (match* adj2 (propensity or coarsened or covariate)) or "propensity score" or ("difference in difference*" or "difference-in-difference*" or "differences in difference*" or "differences-in-difference*" or "double difference*") or ("quasi-experimental" or "quasi experimental" or "quasi-experiment" or "quasi experiment") or ((estimator or counterfactual) and evaluation*) or "instrumental variable*" or (IV adj2 (estimation or approach)) or regression discontinuity or time series or segment* regression).ti,ab,kw. (2983909)

22 Randomized Controlled Trial/ or Random Allocation/ or Evaluation Studies/ or Propensity Score/ or Interrupted Time Series Analysis/ or Controlled Before-After Studies/ or Controlled Clinical Trial/ or Non-Randomized Controlled Trials as Topic/ (896249)

23 or/21-22 (3415351)

24 Cost Analysis/ or Cost-Benefit Analysis/ or Quality-Adjusted Life Years/ or Economics, Medical/ or Cost of Illness/ or Health Care Costs/ or Direct Service Costs/ or Budgets/ or Health Care Sector/ or Public Expenditures/ (193420)

25 (cost-effective* or cost-benefit).ti,ab,kw. (130711)

26 ("life year" or "life years" or qaly* or daly*).ti,ab,kw. (18749)

27 ((economic* or cost*) adj6 (mortality or death* or markov)).ti,ab,kw. (17246)

28 ("cost minimi*" or "cost-utilit*" or "economic evaluation*" or "economic review*" or "cost outcome" or "cost analys*" or "economic analys*" or "budget* impact analys*").ti,ab,kw. (27594)

29 or/24-28 (301485)

30 (review or meta-analysis).pt. (2557796)

31 meta-analysis/ or "systematic review"/ (163880)

32 cochrane database of systematic reviews.jn. (14162)

33 (systematic review or literature review).ti. (130870)

34 or/30-33 (2590211)

35 23 or 29 or 34 (5946083)
36 20 and 35 (8309)
37 exp Animals/ (22312530)
38 Humans/ (17732234)
39 37 not (37 and 38) (4580296)
40 36 not 39 (7984)

2. Embase Classic+Embase (Ovid) <1947 to 2019 May 15> Searched 16th May 2019

1 (immuniz* or immunis* or vaccin* or inoculat* or innoculat* or immunotherap* or prophyla*).ti,ab,kw. (919517)
2 immunization/ or active immunization/ or immunoprophylaxis/ or mass immunization/ or passive immunization/ or secondary immunization/ or exp vaccination/ or vaccination coverage/ (284063)
3 measles mumps vaccine/ or Haemophilus influenzae type b vaccine/ or rubella vaccine/ or diphtheria pertussis tetanus Haemophilus influenzae type b hepatitis B vaccine/ or measles mumps rubella vaccine/ or diphtheria pertussis poliomyelitis tetanus Haemophilus influenzae type b hepatitis B vaccine/ or diphtheria poliomyelitis tetanus vaccine/ or diphtheria tetanus vaccine/ or diphtheria vaccine/ or diphtheria pertussis tetanus Haemophilus influenzae type b vaccine/ or meningitis vaccine/ or measles vaccine/ or BCG vaccine/ or Meningococcus vaccine/ or Japanese encephalitis vaccine/ or diphtheria pertussis poliomyelitis tetanus hepatitis B vaccine/ or measles rubella vaccine/ or mumps rubella vaccine/ or pertussis vaccine/ or diphtheria pertussis tetanus vaccine/ or poliomyelitis vaccine/ or diphtheria pertussis poliomyelitis tetanus Haemophilus influenzae type b vaccine/ or mumps vaccine/ or diphtheria pertussis tetanus hepatitis B vaccine/ or Mycobacterium vaccine/ or triple vaccine/ or diphtheria pertussis poliomyelitis tetanus vaccine/ or varicella zoster vaccine/ or Rotavirus vaccine/ (95305)
4 or/1-3 (1000586)
5 developing country/ (92964)
6 "developing countr*".ti,ab,kw. (74327)
7 (Africa or Asia or Caribbean or West Indies or South America or Latin America or Central America).ti,ab,kw. (238430)
8 Africa/ or Asia/ or Caribbean/ or Caribbean Islands/ or "south and central america"/ or South America/ or Central America/ (156354)
9 (Africa or Central America or South America or Caribbean or Central Asia or Afghanistan or Albania or Algeria or Angola or Argentina or Armenia or Armenian or Azerbaijan or Bangladesh or Benin or Byelarus or Byelorussian or Belarus or Belorussian or Belorussia or Belize or Bhutan or Bolivia or Bosnia or Herzegovina or Hercegovina or Botswana or Brazil or Bulgaria or Burkina Faso or Burkina Fasso or Upper Volta or Burundi or Urundi or Cambodia or Khmer Republic or Kampuchea or Cameroon or Cameroons or Cameron or Camerons or Cape Verde or Cabo Verde or Central African Republic or Chad or Tchad or China or Colombia or Comoros or Comoro Islands or Comores or Mayotte or Congo or Zaire or Costa Rica or Cote d'Ivoire or Ivory Coast or Cuba or Djibouti or French Somaliland or Dominica or Dominican Republic or East Timor or East Timur or Timor Leste or Ecuador or Egypt or United Arab Republic or El Salvador or Eritrea or Ethiopia or Fiji or Gabon or Gabonese Republic or Gambia or Gaza or Georgia Republic or Georgian Republic or Ghana or Grenada or Guatemala or Guinea or Guiana or Guyana or Haiti or Honduras or India or Maldives or Indonesia or Iran or Iraq or Jamaica or Jordan or Kazakhstan or Kazakh or Kenya or Kiribati or Korea or Kosovo or Kyrgyzstan or Kirghizia or Kyrgyz Republic or Kirghiz or Kirgizstan or Lao PDR or Laos or Lebanon or Lesotho or Basutoland or Liberia or Libya or Macedonia or Madagascar or Malagasy Republic or Malaysia or Malaya or Malay or Sabah or Sarawak or Malawi or Mali or Marshall Islands or Mauritania or Mauritius or Agalega Islands or Mexico or Micronesia or Middle East or Moldova or Moldovia or Moldovian or Mongolia or Montenegro or Morocco or Ifni or Mozambique or Myanmar or Myanma or Burma or Namibia or

Nepal or Netherlands Antilles or Nicaragua or Niger or Nigeria or Muscat or Pakistan or Palau or Palestine or Panama or Paraguay or Peru or Philippines or Philipines or Phillipines or Phillippines or Papua New Guinea or Romania or Rumania or Roumania or Rwanda or Ruanda or Saint Lucia or St Lucia or Saint Vincent or St Vincent or Grenadines or Samoa or Samoan Islands or Navigator Island or Navigator Islands or Sao Tome or Senegal or Serbia or Montenegro or Seychelles or Sierra Leone or Sri Lanka or Solomon Islands or Somalia or Sudan or Suriname or Surinam or Swaziland or Eswatini or South Africa or Syria or Tajikistan or Tadzhikistan or Tadjikistan or Tadzhik or Tanzania or Thailand or Togo or Togolese Republic or Tonga or Tunisia or Turkey or Turkmenistan or Turkmen or Uganda or Ukraine or Uzbekistan or Uzbek or Vanuatu or New Hebrides or Venezuela or Vietnam or Viet Nam or West Bank or Yemen or Zambia or Zimbabwe or Rhodesia).ti,ab,kw,sh. (1693062)

10 ((developing or less* developed or least developed or under developed or underdeveloped or middle income or low* income or underserved or under served or deprived or poor* or resource limited or resource constrained) adj (countr* or nation? or population? or world or state*)).ti,ab,kw. (121399)

11 ((developing or less* developed or least developed or under developed or underdeveloped or middle income or low* income or resource limited or resource constrained) adj (economy or economies)).ti,ab,kw. (663)

12 (low* adj (gdp or gnp or gross domestic or gross national)).ti,ab,kw. (335)

13 (low adj3 middle adj3 countr*).ti,ab,kw. (15489)

14 (lmic or lmics or third world or lami countr*).ti,ab,kw. (7992)

15 (transitional countr* or emerging econom* or global south).ti,ab,kw. (1087)

16 or/5-15 (1853015)

17 4 and 16 (104331)

18 (child* or infant* or newborn* or neonat* or neo nat* or prenatal or pre natal or antenatal or antenatal or baby or babies or toddler* or preschool* or parent* or mother* or father* or maternal or paternal).ti,ab,kw. (3061147)

19 boy/ or girl/ or exp infant/ or preschool child/ or toddler/ or parent/ or exp father/ or exp mother/ or grandparent/ or grandfather/ or grandmother/ or caregiver/ or pregnant woman/ (1833747)

20 or/18-19 (3666036)

21 17 and 20 (34101)

22 (random* or experiment* or (match* adj2 (propensity or coarsened or covariate)) or "propensity score" or ("difference in difference*" or "difference-in-difference*" or "differences in difference*" or "differences-in-difference*" or "double difference*") or ("quasi-experimental" or "quasi experimental" or "quasi-experiment" or "quasi experiment") or ((estimator or counterfactual) and evaluation*) or "instrumental variable*" or (IV adj2 (estimation or approach)) or regression discontinuity or time series or segment* regression).ti,ab,kw. (3705127)

23 randomized controlled trial/ or controlled clinical trial/ or equivalence trial/ or non-inferiority trial/ or pragmatic trial/ or superiority trial/ or "randomized controlled trial (topic)"/ or evaluation study/ or exp program evaluation/ or propensity score/ or controlled study/ or time series analysis/ or quasi experimental study/ or instrumental variable analysis/ or mendelian randomization analysis/ (6960490)

24 or/22-23 (9221333)

25 (cost-effective* or cost-benefit).ti,ab,kw. (181445)

26 ("life year" or "life years" or qaly* or daly*).ti,ab,kw. (30322)

27 ((economic* or cost*) adj6 (mortality or death* or markov)).ti,ab,kw. (23530)

28 ("cost minimi*" or "cost-utilit*" or "economic evaluation*" or "economic review*" or "cost outcome" or "cost analys*" or "economic analys*" or "budget* impact analys*").ti,ab,kw. (43685)

29 "health care cost"/ or "drug cost"/ or health care financing/ or budget/ or cost/ or "cost benefit analysis"/ or economic evaluation/ or "cost control"/ or "cost effectiveness analysis"/ or "cost minimization analysis"/ or "cost utility analysis"/ or quality adjusted life year/ or disability-adjusted life year/ or "cost of illness"/ (538960)

30 or/25-29 (633936)
31 "review".pt. or "systematic review"/ or "systematic review (topic)"/ or meta analysis/ or "meta analysis (topic)"/ or cochrane library/ (2700519)
32 (systematic review or literature review).ti. (158376)
33 or/31-32 (2727777)
34 24 or 30 or 33 (11907798)
35 21 and 34 (15343)
36 limit 35 to exclude medline journals (1420)

3. CAB Global Health (Ovid) <1910 to 2019 Week 19> Searched 17th May 2019

1 (immuniz* or immunis* or vaccin* or inoculat* or innoculat* or immunotherap* or prophyla*).ti,ab. (263128)
2 immunization/ or passive immunization/ or vaccination/ or immunization programmes/ or mandatory vaccination/ or oral vaccination/ or vaccines/ or candidate vaccines/ or combined vaccines/ or conjugate vaccines/ or inactivated vaccines/ or live vaccines/ or pertussis vaccines/ or poliomyelitis vaccines/ or polyvalent vaccines/ or bcg vaccine/ or diphtheria pertussis tetanus vaccines/ or diphtheria tetanus pertussis poliomyelitis vaccines/ or measles mumps rubella vaccines/ (122515)
3 or/1-2 (271288)
4 exp africa/ (246366)
5 exp Central America/ or exp Latin America/ or exp South America/ (182184)
6 mexico/ (22593)
7 exp central asia/ (12607)
8 east asia/ or china/ or korea democratic people's republic/ or korea republic/ or mongolia/ (242282)
9 exp south asia/ or himalaya/ (151860)
10 exp south east asia/ or pacific rim/ (98947)
11 exp caribbean/ (22195)
12 exp pacific islands/ (14470)
13 exp developing countries/ (913995)
14 (Africa or Asia or Caribbean or West Indies or South America or Latin America or Central America).tw. (1028925)
15 (Afghanistan or Albania or Algeria or Angola or Argentina or Armenia or Armenian or Azerbaijan or Bangladesh or Benin or Byelarus or Byelorussian or Belarus or Belorussian or Belorussia or Belize or Bhutan or Bolivia or Bosnia or Herzegovina or Hercegovina or Botswana or Brazil or Bulgaria or Burkina Faso or Burkina Fasso or Upper Volta or Burundi or Urundi or Cambodia or Khmer Republic or Kampuchea or Cameroon or Cameroons or Cameron or Camerons or Cape Verde or Central African Republic or Chad or China or Colombia or Comoros or Comoro Islands or Comores or Mayotte or Congo or Zaire or Costa Rica or Cote d'Ivoire or Ivory Coast or Cuba or Djibouti or French Somaliland or Dominica or Dominican Republic or East Timor or East Timur or Timor Leste or Ecuador or Egypt or United Arab Republic or El Salvador or Eritrea or Ethiopia or Fiji or Gabon or Gabonese Republic or Gambia or Gaza or Georgia Republic or Georgian Republic or Ghana or Grenada or Guatemala or Guinea or Guiana or Guyana or Haiti or Honduras or India or Maldives or Indonesia or Iran or Iraq or Jamaica or Jordan or Kazakhstan or Kazakh or Kenya or Kiribati or Korea or Kosovo or Kyrgyzstan or Kirghizia or Kyrgyz Republic or Kirghiz or Kirgizstan or Lao PDR or Laos or Lebanon or Lesotho or Basutoland or Liberia or Libya or Macedonia or Madagascar or Malagasy Republic or Malaysia or Malaya or Malay or Sabah or Sarawak or Malawi or Mali or Marshall Islands or Mauritania or Mauritius or Agalega Islands or Mexico or Micronesia or Middle East or Moldova or Moldovia or Moldovian or Mongolia or Montenegro or Morocco or Ifni or Mozambique or Myanmar or Myanma or Burma or Namibia or Nepal or Netherlands Antilles or Nicaragua or Niger or Nigeria or Muscat or Pakistan or Palau

or Palestine or Panama or Paraguay or Peru or Philippines or Philipines or Phillipines or Phillippines or Papua New Guinea or Romania or Rumania or Roumania or Rwanda or Ruanda or Saint Lucia or St Lucia or Saint Vincent or St Vincent or Grenadines or Samoa or Samoan Islands or Navigator Island or Navigator Islands or Sao Tome or Senegal or Serbia or Montenegro or Seychelles or Sierra Leone or Sri Lanka or Solomon Islands or Somalia or Sudan or Suriname or Surinam or Swaziland or South Africa or Syria or Tajikistan or Tadzhikistan or Tadjikistan or Tadzhik or Tanzania or Thailand or Togo or Togolese Republic or Tonga or Tunisia or Turkey or Turkmenistan or Turkmen or Uganda or Ukraine or Uzbekistan or Uzbek or Vanuatu or New Hebrides or Venezuela or Vietnam or Viet Nam or West Bank or Yemen or Zambia or Zimbabwe).tw. (1003891)

16 ((developing or less* developed or least developed or under developed or underdeveloped or middle income or low* income or underserved or under served or deprived or poor* or resource limited or resource constrained) adj (countr* or nation? or population? or world or state*)).ti,ab. (52582)

17 ((developing or less* developed or least developed or under developed or underdeveloped or middle income or low* income or resource limited or resource constrained) adj (economy or economies)).ti,ab. (278)

18 (low* adj (gdp or gnp or gross domestic or gross national)).tw. (69)

19 (low adj3 middle adj3 countr*).tw. (6904)

20 (lmic or lmics or third world or lami countr*).tw. (22597)

21 (transitional countr* or emerging econom* or global south).ti,ab. (472)

22 or/4-21 (1156687)

23 3 and 22 (84490)

24 (child* or infant* or newborn* or neonat* or neo nat* or prenatal or pre natal or antenatal or antenatal or baby or babies or toddler* or preschool* or parent* or mother* or father* or maternal or paternal).ti,ab. (533665)

25 infants/ or low birth weight infants/ or neonates/ or premature infants/ or preschool children/ or parents/ or fathers/ or grandparents/ or mothers/ or careproviders/ or child careproviders/ or pregnant women/ (166504)

26 or/24-25 (541963)

27 23 and 26 (27216)

28 (random* or experiment* or (match* adj2 (propensity or coarsened or covariate)) or "propensity score" or ("difference in difference*" or "difference-in-difference*" or "differences in difference*" or "differences-in-difference*" or "double difference*") or ("quasi-experimental" or "quasi experimental" or "quasi-experiment" or "quasi experiment") or ((estimator or counterfactual) and evaluation*) or "instrumental variable*" or (IV adj2 (estimation or approach)) or regression discontinuity or time series or segment* regression).ti,ab. (501698)

29 randomized controlled trials/ or program evaluation/ or time series/ or regression analysis/ (39829)

30 or/28-29 (506758)

31 (cost-effective* or cost-benefit).ti,ab. (24530)

32 ("life year" or "life years" or qaly* or daly*).ti,ab. (5193)

33 ((economic* or cost*) adj6 (mortality or death* or markov)).ti,ab. (4632)

34 ("cost minimi*" or "cost-utilit*" or "economic evaluation*" or "economic review*" or "cost outcome" or "cost analys*" or "economic analys*" or "budget* impact analys*").ti,ab. (4648)

35 costs/ or estimated costs/ or health care costs/ or operating costs/ or exp "cost analysis"/ or exp "cost benefit analysis"/ or "cost control"/ or quality-adjusted life year/ or economic evaluation/ or budgets/ or public expenditure/ (31162)

36 or/31-35 (53514)

37 systematic reviews/ or meta-analysis/ (35616)

38 (systematic review or literature review or meta-analy* or metaanaly* or meta analy*).ti. (33890)

39 or/37-38 (42308)

40 30 or 36 or 39 (578827)

41 27 and 40 (5518)

4. Cochrane Library – searched 17th May 2019

#1 (immuniz* or immunis* or vaccin* or inoculat* or innoculat* or immunotherap* or prophyla*):ti,ab,kw 65058
#2 [mh ^immunization] or [mh ^"immunization, passive"] or [mh ^"immunization schedule"] or [mh ^"immunization, secondary"] or [mh ^"immunotherapy, active"] or [mh ^vaccination] or [mh ^"Immunization Programs"] or [mh ^"mass vaccination"] 5349
#3 [mh ^"Tuberculosis Vaccines"] or [mh ^"BCG Vaccine"] or [mh ^"Diphtheria-Tetanus Vaccine"] or [mh ^"Meningococcal Vaccines"] or [mh ^"Pertussis Vaccine"] or [mh ^"Diphtheria-Tetanus-acellular Pertussis Vaccines"] or [mh ^"Diphtheria-Tetanus-Pertussis Vaccine"] or [mh ^"Diphtheria-Tetanus Vaccine"] or [mh ^"Measles Vaccine"] or [mh ^"Mumps Vaccine"] or [mh ^"Rubella Vaccine"] or [mh ^"Measles-Mumps-Rubella Vaccine"] or [mh ^"Poliovirus Vaccines"] or [mh ^"Poliovirus Vaccine, Inactivated"] or [mh ^"Poliovirus Vaccine, Oral"] or [mh ^"Japanese Encephalitis Vaccines"] or [mh ^"Rotavirus Vaccine"] 2605
#4 {OR #1-#3} 65243
#5 MeSH descriptor: [Developing Countries] explode all trees 811
#6 MeSH descriptor: [Africa] explode all trees 6332
#7 MeSH descriptor: [Asia] explode all trees 17824
#8 MeSH descriptor: [Caribbean Region] explode all trees 381
#9 MeSH descriptor: [West Indies] explode all trees 363
#10 MeSH descriptor: [South America] explode all trees 2185
#11 MeSH descriptor: [Latin America] explode all trees 109
#12 MeSH descriptor: [Central America] explode all trees 256
#13 (Africa or Asia or Caribbean or "West Indies" or "South America" or "Latin America" or "Central America"):ti,ab,kw 10930
#14 (Afghanistan or Albania or Algeria or Angola or Argentina or Armenia or Armenian or Azerbaijan or Bangladesh or Benin or Byelarus or Byelorussian or Belarus or Belorussian or Belorussia or Belize or Bhutan or Bolivia or Bosnia or Herzegovina or Hercegovina or Botswana or Brazil or Bulgaria or "Burkina Faso" or "Burkina Fasso" or "Upper Volta" or Burundi or Urundi or Cambodia or "Khmer Republic" or Kampuchea or Cameroon or Cameroons or Cameron or Camerons or "Cape Verde" or "Central African Republic" or Chad or China or Colombia or Comoros or "Comoro Islands" or Comores or Mayotte or Congo or Zaire or "Costa Rica" or "Cote d'Ivoire" or "Ivory Coast" or Croatia or Cuba or Djibouti or "French Somaliland" or Dominica or "Dominican Republic" or "East Timor" or "East Timur" or "Timor Leste" or Ecuador or Egypt or "United Arab Republic" or "El Salvador" or Eritrea or Ethiopia or Fiji or Gabon or "Gabonese Republic" or Gambia or Gaza or "Georgia Republic" or "Georgian Republic" or Ghana or Grenada or Guatemala or Guinea or Guiana or Guyana or Haiti or Honduras or India or Maldives or Indonesia or Iran or Iraq or Jamaica or Jordan or Kazakhstan or Kazakh or Kenya or Kiribati or Korea or Kosovo or Kyrgyzstan or Kirghizia or "Kyrgyz Republic" or Kirghiz or Kirgizstan or "Lao PDR" or Laos or Lebanon or Lesotho or Basutoland or Liberia or Libya or Macedonia or Madagascar or "Malagasy Republic" or Malaysia or Malay or Sabah or Sarawak or Malawi or Mali or "Marshall Islands" or Mauritania or Mauritius or "Agalega Islands" or Mexico or Micronesia or "Middle East" or Moldova or Moldovia or Moldovian or Mongolia or Montenegro or Morocco or Ifni or Mozambique or Myanmar or Myanma or Burma or Namibia or Nepal or "Netherlands Antilles" or "New Caledonia" or Nicaragua or Niger or Nigeria or "Northern Mariana Islands" or Oman or Muscat or Pakistan or Palau or Palestine or Panama or Paraguay or Peru or Philippines or Philipines or Phillipines or Phillippines or "Papua New Guinea" or Portugal or Romania or Rumania or Roumania or Rwanda or Ruanda or "Saint Lucia" or "St Lucia" or "Saint Vincent" or "St Vincent" or Grenadines or Samoa or "Samoan Islands" or "Navigator Island" or "Navigator Islands" or "Sao Tome" or Senegal or Serbia or Montenegro or Seychelles or

"Sierra Leone" or "Sri Lanka" or Ceylon or "Solomon Islands" or Somalia or Sudan or Suriname or Surinam or Swaziland or "South Africa" or Syria or Tajikistan or Tadzhikistan or Tadjikistan or Tadzhik or Tanzania or Thailand or Togo or "Togolese Republic" or Tonga or Trinidad or Tobago or Tunisia or Turkey or Turkmenistan or Turkmen or Uganda or Ukraine or Uzbekistan or Uzbek or Vanuatu or "New Hebrides" or Venezuela or Vietnam or "Viet Nam" or "West Bank" or Yemen or Yugoslavia or Zambia or Zimbabwe):ti,ab,kw 67338

#15 ((developing or "less* developed" or "least developed" or "under developed" or underdeveloped or "middle income" or "low* income" or "resource limited" or "resource constrained") near/1 (economy or economies)):ti,ab,kw 15

#16 ((developing or "less* developed" or "least developed" or "under developed" or underdeveloped or "middle income" or "low* income" or underserved or "under served" or deprived or poor* or "resource limited" or "resource constrained") near/1 (countr* or nation? or population? or world or state*)):ti,ab,kw 6362

#17 (low* next (gdp or gnp or "gross domestic" or "gross national")):ti,ab,kw 49

#18 (low next/3 middle next/3 countr*):ti,ab,kw 1054

#19 (lmic or lmics or "third world" or "lami countr*"):ti,ab,kw 320

#20 ("emerging econom*" or "global south" or "transitional countr*"):ti,ab,kw 5

#21 #5 or #6 or #7 or #8 or #9 or #10 or #11 or #12 or #13 or #14 or #15 or #16 or #17 or #18 or #19 or #20 80139

#22 #4 and #21 7174

#23 (child* or infant* or newborn* or neonat* or "neo nat*" or prenatal or "pre natal" or antenatal or "ante natal" or baby or babies or toddler* or preschool* or parent* or mother* or father* or maternal or paternal):ti,ab,kw 200260

#24 [mh ^Parents] or [mh ^Fathers] or [mh ^Mothers] or [mh ^Grandparents] or [mh ^Caregivers] or [mh ^"Single Parent"] or [mh ^"Pregnant Women"] or [mh ^"Child, Preschool"] or [mh ^Infant] or [mh ^"Infant, Newborn"] or [mh ^"Infant, Low Birth Weight"] or [mh ^"Infant, Small for Gestational Age"] or [mh ^"Infant, Very Low Birth Weight"] or [mh ^"Infant, Extremely Low Birth Weight"] or [mh ^"Infant, Postmature"] or [mh ^"Infant, Premature"] or [mh ^"Infant, Extremely Premature"] 19319

#25 #23 or #24 201672

#26 #22 and #25 3990

#27 (random* or experiment* or (match* near/2 (propensity or coarsened or covariate)) or "propensity score" or ("difference in difference*" or "difference-in-difference*" or "differences in difference*" or "differences-in-difference*" or "double difference*") or ("quasi-experimental" or "quasi experimental" or "quasi-experiment" or "quasi experiment") or ((estimator or counterfactual) and evaluation*) or "instrumental variable*" or (IV near/2 (estimation or approach)) or "regression discontinuity" or "time series" or "segment* regression"):ti,ab,kw 929331

#28 [mh ^"Randomized Controlled Trial"] or [mh ^"Random Allocation"] or [mh ^"Evaluation Studies"] or [mh ^"Propensity Score"] or [mh ^"Interrupted Time Series Analysis"] or [mh ^"Controlled Before-After Studies"] or [mh ^"Controlled Clinical Trial"] or [mh ^"Non-Randomized Controlled Trials as Topic"] 21006

#29 #27 or #28 929342

#30 [mh ^"Cost Analysis"] or [mh ^"Cost-Benefit Analysis"] or [mh ^"Quality-Adjusted Life Years"] or [mh ^"Economics, Medical"] or [mh ^"Cost of Illness"] or [mh ^"Health Care Costs"] or [mh ^"Direct Service Costs"] or [mh ^Budgets] or [mh ^"Health Care Sector"] or [mh ^"Public Expenditures"] 9124

#31 (cost-effective* or cost-benefit):ti,ab,kw 22145

#32 ("life year" or "life years" or qaly* or daly*):ti,ab,kw 5073

#33 ((economic* or cost*) near/6 (mortality or death* or markov)):ti,ab,kw 2822

#34 ("cost minimi*" or "cost-utilit*" or "economic evaluation*" or "economic review*" or "cost outcome" or "cost analys*" or "economic analys*" or "budget* impact analys*"):ti,ab,kw 3517

#35 {OR #30-#34} 27818
#36 (review or meta-analysis):pt 26656
#37 ("systematic review" or "literature review"):ti 5036
#38 {OR #36-#37} 29887
#39 #29 or #35 or #38 936279
#40 #26 and #39 3055

5. PsycINFO (Ovid) <1806 to May Week 2 2019> Searched 18th May 2019

1 (immuniz* or immunis* or vaccin* or inoculat* or innoculat* or immunotherap* or prophyla*).ti,ab. (17647)
2 immunization/ or immunotherapy/ (5068)
3 or/1-2 (18043)
4 (Afghanistan or Albania or Algeria or Angola or Argentina or Armenia or Armenian or Azerbaijan or Bangladesh or Benin or Byelarus or Byelorussian or Belarus or Belorussian or Belorussia or Belize or Bhutan or Bolivia or Bosnia or Herzegovina or Hercegovina or Botswana or Brazil or Bulgaria or Burkina Faso or Burkina Fasso or Upper Volta or Burundi or Urundi or Cambodia or Khmer Republic or Kampuchea or Cameroon or Cameroons or Cameron or Camerons or Cape Verde or Central African Republic or Chad or China or Colombia or Comoros or Comoro Islands or Comores or Mayotte or Congo or Zaire or Costa Rica or Cote d'Ivoire or Ivory Coast or Cuba or Djibouti or French Somaliland or Dominica or Dominican Republic or East Timor or East Timur or Timor Leste or Ecuador or Egypt or United Arab Republic or El Salvador or Eritrea or Ethiopia or Fiji or Gabon or Gabonese Republic or Gambia or Gaza or Georgia Republic or Georgian Republic or Ghana or Grenada or Guatemala or Guinea or Guiana or Guyana or Haiti or Honduras or India or Maldives or Indonesia or Iran or Iraq or Jamaica or Jordan or Kazakhstan or Kazakh or Kenya or Kiribati or Korea or Kosovo or Kyrgyzstan or Kirghizia or Kyrgyz Republic or Kirghiz or Kirgizstan or Lao PDR or Laos or Lebanon or Lesotho or Basutoland or Liberia or Libya or Macedonia or Madagascar or Malagasy Republic or Malaysia or Malaya or Malay or Sabah or Sarawak or Malawi or Mali or Marshall Islands or Mauritania or Mauritius or Agalega Islands or Mexico or Micronesia or Middle East or Moldova or Moldovia or Moldovian or Mongolia or Montenegro or Morocco or Ifni or Mozambique or Myanmar or Myanma or Burma or Namibia or Nepal or Netherlands Antilles or Nicaragua or Niger or Nigeria or Muscat or Pakistan or Palau or Palestine or Panama or Paraguay or Peru or Philippines or Philipines or Phillipines or Phillippines or Papua New Guinea or Romania or Rumania or Roumania or Rwanda or Ruanda or Saint Lucia or St Lucia or Saint Vincent or St Vincent or Grenadines or Samoa or Samoan Islands or Navigator Island or Navigator Islands or Sao Tome or Senegal or Serbia or Montenegro or Seychelles or Sierra Leone or Sri Lanka or Solomon Islands or Somalia or Sudan or Suriname or Surinam or Swaziland or South Africa or Syria or Tajikistan or Tadzhikistan or Tadjikistan or Tadzhik or Tanzania or Thailand or Togo or Togolese Republic or Tonga or Tunisia or Turkey or Turkmenistan or Turkmen or Uganda or Ukraine or Uzbekistan or Uzbek or Vanuatu or New Hebrides or Venezuela or Vietnam or Viet Nam or West Bank or Yemen or Zambia or Zimbabwe).hw,ti,ab,cp,lo. (249271)
5 (Africa or Asia or Caribbean or West Indies or Middle East or South America or Latin America or Central America).hw,ti,ab,cp,lo. (47894)
6 ((developing or less* developed or least developed or under developed or underdeveloped or middle income or low* income or resource limited or resource constrained) adj (economy or economies)).ti,ab. (350)
7 ((developing or less* developed or least developed or under developed or underdeveloped or middle income or low* income or underserved or under served or deprived or poor* or resource limited or resource constrained) adj (countr* or nation? or population? or world or state*)).ti,ab. (16821)
8 (low* adj (gdp or gnp or gross domestic or gross national)).ti,ab. (42)
9 (low adj3 middle adj3 countr*).ti,ab. (2687)
10 (lmic or lmics or third world or lami countr*).ti,ab. (1637)

11 (transitional countr* or emerging econom* or global south).ti,ab. (1295)
12 developing countries/ or emerging economies/ (6069)
13 or/4-12 (275101)
14 3 and 13 (2041)
15 (child* or infant* or newborn* or neonat* or neo nat* or prenatal or pre natal or ante natal or antenatal or baby or babies or toddler* or preschool* or parent* or mother* or father* or maternal or paternal).ti,ab. (851334)
16 (childhood birth 12 yrs or infancy 2 23 mo or neonatal birth 1 mo or preschool age 2 5 yrs).ag. (517691)
17 family members/ or grandparents/ or exp parents/ or caregivers/ (126795)
18 or/15-17 (997122)
19 14 and 18 (944)
20 (random* or experiment* or (match* adj2 (propensity or coarsened or covariate)) or "propensity score" or ("difference in difference*" or "difference-in-difference*" or "differences in difference*" or "differences-in-difference*" or "double difference*") or ("quasi-experimental" or "quasi experimental" or "quasi-experiment" or "quasi experiment") or ((estimator or counterfactual) and evaluation*) or "instrumental variable*" or (IV adj2 (estimation or approach)) or regression discontinuity or time series or segment* regression).ti,ab. (571101)
21 experimental design/ or experimental methods/ or exp clinical trials/ or quantitative methods/ or quasi experimental methods/ or exp statistical regression/ or time series/ (41453)
22 or/20-21 (594997)
23 (cost-effective* or cost-benefit).ti,ab. (16148)
24 ("life year" or "life years" or qaly* or daly*).ti,ab. (2367)
25 ((economic* or cost*) adj6 (mortality or death* or markov)).ti,ab. (1335)
26 ("cost minimi*" or "cost-utilit*" or "economic evaluation*" or "economic review*" or "cost outcome" or "cost analys*" or "economic analys*" or "budget* impact analys*").ti,ab. (3788)
27 "costs and cost analysis"/ or budgets/ or health care costs/ or "cost containment"/ or funding/ or health care economics/ or resource allocation/ (31851)
28 or/23-27 (46005)
29 "literature review"/ or meta analysis/ (26684)
30 (systematic review or literature review).ti. (20723)
31 or/29-30 (46733)
32 22 or 28 or 31 (670259)
33 19 and 32 (197)

6. CINAHL (Ebsco) – Searched 18th May 2019

S45 S22 AND S44 Database - CINAHL Plus with Full Text
1,672
S44 S29 OR S39 OR S43
762,995
S43 S40 OR S41 OR S42
124,839
S42 TI("systematic review" or "literature review")
65,112
S41 (MH "Meta Analysis") OR (MH "Meta Synthesis")
39,339
S40 (MH "Literature Review") OR (MH "Systematic Review") OR (MH "Scoping Review")
76,956
S39 S30 OR S31 OR S32 OR S33 OR S34 OR S35 OR S36 OR S37 OR S38

151,231
S38 (MH "Public Expenditures") OR (MH "Resource Allocation") OR (MH "Health Resource Allocation")
10,627
S37 (MH "Budgets")
9,232
S36 (MH "Economic Aspects of Illness")
8,290
S35 (MH "Quality-Adjusted Life Years") OR (MH "Disability-Adjusted Life Years")
4,021
S34 (MH "Costs and Cost Analysis+") OR (MH "Health Care Costs+") OR (MH "Cost Control+")
102,662
S33 TI("cost minimi*" or "cost-utilit*" or "economic evaluation*" or "economic review*" or "cost outcome" or "cost analys*" or "economic analys*" or "budget* impact analys*") OR AB("cost minimi*" or "cost-utilit*" or "economic evaluation*" or "economic review*" or "cost outcome" or "cost analys*" or "economic analys*" or "budget* impact analys*") OR SU("cost minimi*" or "cost-utilit*" or "economic evaluation*" or "economic review*" or "cost outcome" or "cost analys*" or "economic analys*" or "budget* impact analys*")
23,886
S32 TI((economic* or cost*) N6 (mortality or death* or markov)) OR AB((economic* or cost*) N6 (mortality or death* or markov)) OR SU((economic* or cost*) N6 (mortality or death* or markov))
5,722
S31 TI("life year" or "life years" or qaly* or daly*) OR AB("life year" or "life years" or qaly* or daly*) OR SU("life year" or "life years" or qaly* or daly*)
8,344
S30 TI(cost-effective* or cost-benefit) OR AB(cost-effective* or cost-benefit) OR SU(cost-effective* or cost-benefit)
51,463
S29 S23 OR S24 OR S25 OR S26 OR S27 OR S28
544,911
S28 (MH "Quasi-Experimental Studies")
10,873
S27 (MH "Interrupted Time Series Analysis") OR (MH "Controlled Before-After Studies") OR (MH "Nonrandomized Trials")
791
S26 (MH "Evaluation Research")
114,845
S25 (MH "Random Assignment")
54,711
S24 (MH "Randomized Controlled Trials") OR (MH "Equivalence Trials")
81,940
S23 TI(random* or experiment* or (match* N2 (propensity or coarsened or covariate)) or "propensity score" or ("difference in difference*" or "difference-in-difference*" or "differences in difference*" or "differences-in-difference*" or "double difference*") or ("quasi-experimental" or "quasi experimental" or "quasi-experiment" or "quasi experiment") or ((estimator or counterfactual) and evaluation*) or "instrumental variable*" or (IV N2 (estimation or approach)) or "regression discontinuity" or "time series" or "segment* regression") OR AB(random* or experiment* or (match* N2 (propensity or coarsened or covariate)) or "propensity score" or ("difference in difference*" or "difference-in-difference*" or "differences in difference*" or "differences-in-difference*" or "double difference*") or ("quasi-experimental" or "quasi experimental" or "quasi-experiment" or "quasi experiment") or ((estimator or counterfactual) and evaluation*) or "instrumental

variable*" or (IV N2 (estimation or approach)) or "regression discontinuity" or "time series" or "segment* regression") OR SU(random* or experiment* or (match* N2 (propensity or coarsened or covariate)) or "propensity score" or ("difference in difference*" or "difference-in-difference*" or "differences in difference*" or "differences-in-difference*" or "double difference*") or ("quasi-experimental" or "quasi experimental" or "quasi-experiment" or "quasi experiment") or ((estimator or counterfactual) and evaluation*) or "instrumental variable*" or (IV N2 (estimation or approach)) or "regression discontinuity" or "time series" or "segment* regression")
465,698

S22 S15 AND S21
6,658

S21 S16 OR S17 OR S18 OR S19 OR S20
913,473

S20 (MH "Caregivers")
30,125

S19 (MH "Grandparents")
1,885

S18 (MH "Parents") OR (MH "Fathers") OR (MH "Mothers") OR (MH "Expectant Mothers")
70,610

S17 (MH "Child, Preschool") OR (MH "Infant+")
312,965

S16 TI(child* or infant* or newborn* or neonat* or "neo nat*" or prenatal or "pre natal" or "ante natal" or antenatal or baby or babies or toddler* or preschool* or parent* or mother* or father* or maternal or paternal) OR AB(child* or infant* or newborn* or neonat* or "neo nat*" or prenatal or "pre natal" or "ante natal" or antenatal or baby or babies or toddler* or preschool* or parent* or mother* or father* or maternal or paternal) OR SU(child* or infant* or newborn* or neonat* or "neo nat*" or prenatal or "pre natal" or "ante natal" or antenatal or baby or babies or toddler* or preschool* or parent* or mother* or father* or maternal or paternal)
891,134

S15 S4 AND S14
(14,504)

S14 S5 OR S6 OR S7 OR S8 OR S9 OR S10 OR S11 OR S12 OR S13
469,011

S13 TI ("transitional countr*" OR "emerging econom*" or "global south") OR AB ("transitional countr*" OR "emerging econom*" or "global south") OR SU ("transitional countr*" OR "emerging econom*" or "global south")
354

S12 TI (lmic or lmics or "third world" or "lami countr*") OR AB (lmic or lmics or "third world" or "lami countr*") OR SU (lmic or lmics or "third world" or "lami countr*")
1,821

S11 TI (low N3 middle N3 countr*) OR AB (low N3 middle N3 countr*) OR SU (low N3 middle N3 countr*)

6,168

S10 TI (low* N1 (gdp or gnp or "gross domestic" or "gross national" or GNI)) OR AB (low* N1 (gdp or gnp or "gross domestic" or "gross national" or GNI)) OR SU (low* N1 (gdp or gnp or "gross domestic" or "gross national" or GNI))
71

S9 TI ((developing or "less* developed" or "under developed" or underdeveloped or "middle income" or "low* income") N1 (economy or economies)) OR AB ((developing or "less* developed" or "under developed" or underdeveloped or "middle income" or "low* income") N1 (economy or economies)) OR SU ((developing or

"less* developed" or "under developed" or underdeveloped or "middle income" or "low* income") N1 (economy or economies))

120

S8 TI((developing or "less* developed" or "least developed" or "under developed" or underdeveloped or "middle income" or "low* income" or underserved or "under served" or deprived or poor* or "resource limited" or "resource constrained") N1 (countr* or nation? or population? or world or state*)) OR AB((developing or "less* developed" or "least developed" or "under developed" or underdeveloped or "middle income" or "low* income" or underserved or "under served" or deprived or poor* or "resource limited" or "resource constrained") N1 (countr* or nation? or population? or world or state*)) OR SU ((developing or "less* developed" or "least developed" or "under developed" or underdeveloped or "middle income" or "low* income" or underserved or "under served" or deprived or poor* or "resource limited" or "resource constrained") N1 (countr* or nation? or population? or world or state*))

37,075

S7 TI (Africa or Asia or Caribbean or "West Indies" or "South America" or "Latin America" or "Central America") OR AB (Africa or Asia or Caribbean or "West Indies" or "South America" or "Latin America" or "Central America") OR SU (Africa or Asia or Caribbean or "West Indies" or "South America" or "Latin America" or "Central America")

58,567

S6 TI (Afghanistan or Albania or Algeria or Angola or Antigua or Barbuda or Argentina or Armenia or Armenian or Aruba or Azerbaijan or Bangladesh or Benin or Byelarus or Byelorussian or Belarus or Belorussian or Belorussia or Belize or Bhutan or Bolivia or Bosnia or Herzegovina or Hercegovina or Botswana or Brazil or Bulgaria or "Burkina Faso" or "Burkina Fasso" or "Upper Volta" or Burundi or Urundi or Cambodia or "Khmer Republic" or Kampuchea or Cameroon or Cameroons or Cameron or Camerons or "Cape Verde" or "Central African Republic" or Chad or Chile or China or Colombia or Comoros or "Comoro Islands" or Comores or Mayotte or Congo or Zaire or "Costa Rica" or "Cote d'Ivoire" or "Ivory Coast" or Croatia or Cuba or Djibouti or "French Somaliland" or Dominica or "Dominican Republic" or "East Timor" or "East Timur" or "Timor Leste" or Ecuador or Egypt or "United Arab Republic" or "El Salvador" or Eritrea or Ethiopia or Fiji or Gabon or "Gabonese Republic" or Gambia or Gaza or "Georgia Republic" or "Georgian Republic" or Ghana or "Gold Coast" or Grenada or Guatemala or Guinea or Guam or Guiana or Guyana or Haiti or Honduras or India or Maldives or Indonesia or Iran or Iraq or Jamaica or Jordan or Kazakhstan or Kazakh or Kenya or Kiribati or Korea or Kosovo or Kyrgyzstan or Kirghizia or "Kyrgyz Republic" or Kirghiz or Kirgizstan or "Lao PDR" or Laos or Latvia or Lebanon or Lesotho or Basutoland or Liberia or Libya or Lithuania or Macedonia or Madagascar or "Malagasy Republic" or Malaysia or Malaya or Malay or Sabah or Sarawak or Malawi or Mali or "Marshall Islands" or Mauritania or Mauritius or "Agalega Islands" or Mexico or Micronesia or "Middle East" or Moldova or Moldovia or Moldovian or Mongolia or Montenegro or Morocco or Ifni or Mozambique or Myanmar or Myanma or Burma or Namibia or Nepal or "Netherlands Antilles" or "New Caledonia" or Nicaragua or Niger or Nigeria or Oman or Muscat or Pakistan or Palau or Palestine or Panama or Paraguay or Peru or Philippines or Philipines or Phillipines or Phillippines or Romania or Rumania or Roumania or Russia or Russian or Rwanda or Ruanda or "Saint Lucia" or "St Lucia" or "Saint Vincent" or "St Vincent" or Grenadines or Samoa or "Samoan Islands" or "Navigator Island" or "Navigator Islands" or "Sao Tome" or Senegal or Serbia or Montenegro or Seychelles or "Sierra Leone" or "Sri Lanka" or Ceylon or "Solomon Islands" or Somalia or Sudan or Suriname or Surinam or Swaziland or Syria or Tajikistan or Tadzhikistan or Tadjikistan or Tadzhik or Tanzania or Thailand or Togo or "Togolese Republic" or Tonga or Trinidad or Tobago or Tunisia or Turkey or Turkmenistan or Turkmen or Tuvalu or Uganda or Ukraine or Uruguay or Uzbekistan or Uzbek or Vanuatu or "New Hebrides" or Venezuela or Vietnam or "Viet Nam" or "West Bank" or Yemen or Yugoslavia or Zambia or Zimbabwe) OR AB (Afghanistan or Albania or Algeria or Angola or Antigua or Barbuda or Argentina or Armenia or Armenian or Aruba or Azerbaijan or Bangladesh or Benin or Byelarus or Byelorussian or Belarus or Belorussian or Belorussia or Belize or Bhutan or Bolivia or

Bosnia or Herzegovina or Hercegovina or Botswana or Brazil or Bulgaria or "Burkina Faso" or "Burkina Fasso" or "Upper Volta" or Burundi or Urundi or Cambodia or "Khmer Republic" or Kampuchea or Cameroon or Cameroons or Cameron or Camerons or "Cape Verde" or "Central African Republic" or Chad or Chile or China or Colombia or Comoros or "Comoro Islands" or Comores or Mayotte or Congo or Zaire or "Costa Rica" or "Cote d'Ivoire" or "Ivory Coast" or Croatia or Cuba or Djibouti or "French Somaliland" or Dominica or "Dominican Republic" or "East Timor" or "East Timur" or "Timor Leste" or Ecuador or Egypt or "United Arab Republic" or "El Salvador" or Eritrea or Ethiopia or Fiji or Gabon or "Gabonese Republic" or Gambia or Gaza or "Georgia Republic" or "Georgian Republic" or Ghana or "Gold Coast" or Grenada or Guatemala or Guinea or Guam or Guiana or Guyana or Haiti or Honduras or India or Maldives or Indonesia or Iran or Iraq or Jamaica or Jordan or Kazakhstan or Kazakh or Kenya or Kiribati or Korea or Kosovo or Kyrgyzstan or Kirghizia or "Kyrgyz Republic" or Kirghiz or Kirgizstan or "Lao PDR" or Laos or Latvia or Lebanon or Lesotho or Basutoland or Liberia or Libya or Lithuania or Macedonia or Madagascar or "Malagasy Republic" or Malaysia or Malaya or Malay or Sabah or Sarawak or Malawi or Mali or "Marshall Islands" or Mauritania or Mauritius or "Agalega Islands" or Mexico or Micronesia or "Middle East" or Moldova or Moldovia or Moldovian or Mongolia or Montenegro or Morocco or Ifni or Mozambique or Myanmar or Myanma or Burma or Namibia or Nepal or "Netherlands Antilles" or "New Caledonia" or Nicaragua or Niger or Nigeria or Oman or Muscat or Pakistan or Palau or Palestine or Panama or Paraguay or Peru or Philippines or Philipines or Phillipines or Phillippines or Romania or Rumania or Roumania or Russia or Russian or Rwanda or Ruanda or "Saint Lucia" or "St Lucia" or "Saint Vincent" or "St Vincent" or Grenadines or Samoa or "Samoan Islands" or "Navigator Island" or "Navigator Islands" or "Sao Tome" or Senegal or Serbia or Montenegro or Seychelles or "Sierra Leone" or "Sri Lanka" or Ceylon or "Solomon Islands" or Somalia or Sudan or Suriname or Surinam or Swaziland or Syria or Tajikistan or Tadzhikistan or Tadjikistan or Tadzhik or Tanzania or Thailand or Togo or "Togolese Republic" or Tonga or Trinidad or Tobago or Tunisia or Turkey or Turkmenistan or Turkmen or Tuvalu or Uganda or Ukraine or Uruguay or Uzbekistan or Uzbek or Vanuatu or "New Hebrides" or Venezuela or Vietnam or "Viet Nam" or "West Bank" or Yemen or Yugoslavia or Zambia or Zimbabwe) OR SU (Afghanistan or Albania or Algeria or Angola or Antigua or Barbuda or Argentina or Armenia or Armenian or Aruba or Azerbaijan or Bangladesh or Benin or Byelarus or Byelorussian or Belarus or Belorussian or Belorussia or Belize or Bhutan or Bolivia or Bosnia or Herzegovina or Hercegovina or Botswana or Brazil or Bulgaria or "Burkina Faso" or "Burkina Fasso" or "Upper Volta" or Burundi or Urundi or Cambodia or "Khmer Republic" or Kampuchea or Cameroon or Cameroons or Cameron or Camerons or "Cape Verde" or "Central African Republic" or Chad or Chile or China or Colombia or Comoros or "Comoro Islands" or Comores or Mayotte or Congo or Zaire or "Costa Rica" or "Cote d'Ivoire" or "Ivory Coast" or Croatia or Cuba or Djibouti or "French Somaliland" or Dominica or "Dominican Republic" or "East Timor" or "East Timur" or "Timor Leste" or Ecuador or Egypt or "United Arab Republic" or "El Salvador" or Eritrea or Ethiopia or Fiji or Gabon or "Gabonese Republic" or Gambia or Gaza or "Georgia Republic" or "Georgian Republic" or Ghana or "Gold Coast" or Grenada or Guatemala or Guinea or Guam or Guiana or Guyana or Haiti or Honduras or India or Maldives or Indonesia or Iran or Iraq or Jamaica or Jordan or Kazakhstan or Kazakh or Kenya or Kiribati or Korea or Kosovo or Kyrgyzstan or Kirghizia or "Kyrgyz Republic" or Kirghiz or Kirgizstan or "Lao PDR" or Laos or Latvia or Lebanon or Lesotho or Basutoland or Liberia or Libya or Lithuania or Macedonia or Madagascar or "Malagasy Republic" or Malaysia or Malaya or Malay or Sabah or Sarawak or Malawi or Mali or "Marshall Islands" or Mauritania or Mauritius or "Agalega Islands" or Mexico or Micronesia or "Middle East" or Moldova or Moldovia or Moldovian or Mongolia or Montenegro or Morocco or Ifni or Mozambique or Myanmar or Myanma or Burma or Namibia or Nepal or "Netherlands Antilles" or "New Caledonia" or Nicaragua or Niger or Nigeria or Oman or Muscat or Pakistan or Palau or Palestine or Panama or Paraguay or Peru or Philippines or Philipines or Phillipines or Phillippines or Romania or Rumania or Roumania or Russia or Russian or Rwanda or Ruanda or "Saint Lucia" or "St Lucia" or "Saint Vincent" or "St Vincent" or Grenadines or Samoa or "Samoan Islands" or "Navigator Island" or "Navigator Islands" or "Sao Tome" or Senegal or Serbia or Montenegro or Seychelles or "Sierra Leone" or "Sri Lanka" or Ceylon or "Solomon

Islands" or Somalia or Sudan or Suriname or Surinam or Swaziland or Syria or Tajikistan or Tadzhikistan or Tadjikistan or Tadzhik or Tanzania or Thailand or Togo or "Togolese Republic" or Tonga or Trinidad or Tobago or Tunisia or Turkey or Turkmenistan or Turkmen or Tuvalu or Uganda or Ukraine or Uruguay or Uzbekistan or Uzbek or Vanuatu or "New Hebrides" or Venezuela or Vietnam or "Viet Nam" or "West Bank" or Yemen or Yugoslavia or Zambia or Zimbabwe)

337,608

S5 MH (Africa+ OR Asia+ OR "South America+" OR "Central America+" OR Mexico OR "West Indies+" OR "Atlantic Islands+" OR Armenia OR Azerbaijan OR "Georgia (Republic)" OR "Indian Ocean Islands" OR "Pacific Islands")

366,684

S4 S1 OR S2 OR S3

(105,391)

S3 (MH "Pertussis Vaccine+") OR (MH "Vaccines, Combined+") OR (MH "Chickenpox Vaccine") OR (MH "Japanese Encephalitis Vaccines") OR (MH "Measles Vaccine+") OR (MH "Measles-Mumps-Rubella Vaccine+") OR (MH "Mumps Vaccine+") OR (MH "Poliovirus Vaccine+") OR (MH "Rubella Vaccine+") OR (MH "BCG Vaccine") OR (MH "Diphtheria-Tetanus Vaccine") OR (MH "Diphtheria-Tetanus-acellular Pertussis Vaccines") OR (MH "Diphtheria-Tetanus-Pertussis Vaccine") OR (MH "Meningococcal Vaccines") OR (MH "Rotavirus Vaccines")

8,714

S2 (MH "Vaccination Coverage") OR (MH "Pre-Exposure Prophylaxis") OR (MH "Immunization") OR (MH "Immunization Schedule") OR (MH "Immunization, Secondary")

23,373

S1 TI(immuniz* or immunis* or vaccin* or inoculat* or innoculat* or immunotherap* or prophyla*) OR AB(immuniz* or immunis* or vaccin* or inoculat* or innoculat* or immunotherap* or prophyla*) OR SU(immuniz* or immunis* or vaccin* or inoculat* or innoculat* or immunotherap* or prophyla*)

(105,391)

7. **Ebsco Discovery – Searched 19th May 2019 – limited to: Academic Search Complete (2411); Africa-Wide (2170); Ideas-Repec (123); Econlit (67); World Bank e-library (23)**

S21 S13 AND S20 Interface - EBSCO Discovery Service

17,156

S20 S14 OR S15 OR S16 OR S17 OR S18 OR S19

17,411,786

S19 TI (("systematic review" or "literature review" or meta-analy* or metaanaly* or "meta analy*")) OR SU (("systematic review" or "literature review" or meta-analy* or metaanaly* or "meta analy*"))

706,007

S18 TI("cost minimi*" or "cost-utilit*" or "economic evaluation*" or "economic review*" or "cost outcome" or "cost analys*" or "economic analys*" or "budget* impact analys*") OR AB("cost minimi*" or "cost-utilit*" or "economic evaluation*" or "economic review*" or "cost outcome" or "cost analys*" or "economic analys*" or "budget* impact analys*") OR SU("cost minimi*" or "cost-utilit*" or "economic evaluation*" or "economic review*" or "cost outcome" or "cost analys*" or "economic analys*" or "budget* impact analys*")

497,100

S17 TI((economic* or cost*) N6 (mortality or death* or markov)) OR AB((economic* or cost*) N6 (mortality or death* or markov)) OR SU((economic* or cost*) N6 (mortality or death* or markov))

. 69,853

S16 TI("life year" or "life years" or qaly* or daly*) OR AB("life year" or "life years" or qaly* or daly*) OR SU("life year" or "life years" or qaly* or daly*)
133,045

S15 TI(cost-effective* or cost-benefit) OR AB(cost-effective* or cost-benefit) OR SU(cost-effective* or cost-benefit)
848,452

S14 TI(random* or experiment* or (match* N2 (propensity or coarsened or covariate)) or "propensity score" or ("difference in difference*" or "difference-in-difference*" or "differences in difference*" or "differences-in-difference*" or "double difference*") or ("quasi-experimental" or "quasi experimental" or "quasi-experiment" or "quasi experiment") or ((estimator or counterfactual) and evaluation*) or "instrumental variable*" or (IV N2 (estimation or approach)) or "regression discontinuity" or "time series" or "segment* regression") OR AB(random* or experiment* or (match* N2 (propensity or coarsened or covariate)) or "propensity score" or ("difference in difference*" or "difference-in-difference*" or "differences in difference*" or "differences-in-difference*" or "double difference*") or ("quasi-experimental" or "quasi experimental" or "quasi-experiment" or "quasi experiment") or ((estimator or counterfactual) and evaluation*) or "instrumental variable*" or (IV N2 (estimation or approach)) or "regression discontinuity" or "time series" or "segment* regression") OR SU(random* or experiment* or (match* N2 (propensity or coarsened or covariate)) or "propensity score" or ("difference in difference*" or "difference-in-difference*" or "differences in difference*" or "differences-in-difference*" or "double difference*") or ("quasi-experimental" or "quasi experimental" or "quasi-experiment" or "quasi experiment") or ((estimator or counterfactual) and evaluation*) or "instrumental variable*" or (IV N2 (estimation or approach)) or "regression discontinuity" or "time series" or "segment* regression")
. 21,537,647

S13 S11 AND S12
82,102

S12 TI(child* or infant* or newborn* or neonat* or "neo nat*" or prenatal or "pre natal" or "ante natal" or antenatal or baby or babies or toddler* or preschool* or parent* or mother* or father* or maternal or paternal) OR AB(child* or infant* or newborn* or neonat* or "neo nat*" or prenatal or "pre natal" or "ante natal" or antenatal or baby or babies or toddler* or preschool* or parent* or mother* or father* or maternal or paternal) OR SU(child* or infant* or newborn* or neonat* or "neo nat*" or prenatal or "pre natal" or "ante natal" or antenatal or baby or babies or toddler* or preschool* or parent* or mother* or father* or maternal or paternal)
12,363,410

S11 S1 AND S10
302,160

S10 S2 OR S3 OR S4 OR S5 OR S6 OR S7 OR S8 OR S9
17,426,742

S9 TI ("transitional countr*" OR "emerging econom*" or "global south") OR AB ("transitional countr*" OR "emerging econom*" or "global south") OR SU ("transitional countr*" OR "emerging econom*" or "global south")
77,803

S8 TI (lmic or lmics or "third world" or "lami countr*") OR AB (lmic or lmics or "third world" or "lami countr*") OR SU (lmic or lmics or "third world" or "lami countr*")
86,226

S7 TI (low N3 middle N3 countr*) OR AB (low N3 middle N3 countr*) OR SU (low N3 middle N3 countr*)
61,578

S6 TI (low* N1 (gdp or gnp or "gross domestic" or "gross national" or GNI)) OR AB (low* N1 (gdp or gnp or "gross domestic" or "gross national" or GNI)) OR SU (low* N1 (gdp or gnp or "gross domestic" or "gross national" or GNI))

4,541

S5 TI ((developing or "less* developed" or "under developed" or underdeveloped or "middle income" or "low* income") N1 (economy or economies)) OR AB ((developing or "less* developed" or "under developed" or underdeveloped or "middle income" or "low* income") N1 (economy or economies)) OR SU ((developing or "less* developed" or "under developed" or underdeveloped or "middle income" or "low* income") N1 (economy or economies))

57,068

S4 TI((developing or "less* developed" or "least developed" or "under developed" or underdeveloped or "middle income" or "low* income" or underserved or "under served" or deprived or poor* or "resource limited" or "resource constrained") N1 (countr* or nation? or population? or world or state*)) OR AB((developing or "less* developed" or "least developed" or "under developed" or underdeveloped or "middle income" or "low* income" or underserved or "under served" or deprived or poor* or "resource limited" or "resource constrained") N1 (countr* or nation? or population? or world or state*)) OR SU ((developing or "less* developed" or "least developed" or "under developed" or underdeveloped or "middle income" or "low* income" or underserved or "under served" or deprived or poor* or "resource limited" or "resource constrained") N1 (countr* or nation? or population? or world or state*))

1,137,178

S3 TI (Africa or Asia or Caribbean or "West Indies" or "South America" or "Latin America" or "Central America") OR AB (Africa or Asia or Caribbean or "West Indies" or "South America" or "Latin America" or "Central America") OR SU (Africa or Asia or Caribbean or "West Indies" or "South America" or "Latin America" or "Central America")

5,836,383

S2 TI (Afghanistan or Albania or Algeria or Angola or Antigua or Barbuda or Argentina or Armenia or Armenian or Aruba or Azerbaijan or Bangladesh or Benin or Byelarus or Byelorussian or Belarus or Belorussian or Belorussia or Belize or Bhutan or Bolivia or Bosnia or Herzegovina or Hercegovina or Botswana or Brazil or Bulgaria or "Burkina Faso" or "Burkina Fasso" or "Upper Volta" or Burundi or Urundi or Cambodia or "Khmer Republic" or Kampuchea or Cameroon or Cameroons or Cameron or Camerons or "Cape Verde" or "Central African Republic" or Chad or Chile or China or Colombia or Comoros or "Comoro Islands" or Comores or Mayotte or Congo or Zaire or "Costa Rica" or "Cote d'Ivoire" or "Ivory Coast" or Croatia or Cuba or Djibouti or "French Somaliland" or Dominica or "Dominican Republic" or "East Timor" or "East Timur" or "Timor Leste" or Ecuador or Egypt or "United Arab Republic" or "El Salvador" or Eritrea or Ethiopia or Fiji or Gabon or "Gabonese Republic" or Gambia or Gaza or "Georgia Republic" or "Georgian Republic" or Ghana or "Gold Coast" or Grenada or Guatemala or Guinea or Guam or Guiana or Guyana or Haiti or Honduras or India or Maldives or Indonesia or Iran or Iraq or Jamaica or Jordan or Kazakhstan or Kazakh or Kenya or Kiribati or Korea or Kosovo or Kyrgyzstan or Kirghizia or "Kyrgyz Republic" or Kirghiz or Kirgizstan or "Lao PDR" or Laos or Latvia or Lebanon or Lesotho or Basutoland or Liberia or Libya or Lithuania or Macedonia or Madagascar or "Malagasy Republic" or Malaysia or Malaya or Malay or Sabah or Sarawak or Malawi or Mali or "Marshall Islands" or Mauritania or Mauritius or "Agalega Islands" or Mexico or Micronesia or "Middle East" or Moldova or Moldovia or Moldovian or Mongolia or Montenegro or Morocco or Ifni or Mozambique or Myanmar or Myanma or Burma or Namibia or Nepal or "Netherlands Antilles" or "New Caledonia" or Nicaragua or Niger or Nigeria or Oman or Muscat or Pakistan or Palau or Palestine or Panama or Paraguay or Peru or Philippines or Philipines or Phillipines or Phillippines or Romania or Rumania or Roumania or Russia or Russian or Rwanda or Ruanda or "Saint Lucia" or "St Lucia" or "Saint Vincent" or "St Vincent" or Grenadines or Samoa or "Samoan Islands" or "Navigator Island" or "Navigator Islands" or "Sao Tome" or Senegal or Serbia or Montenegro or Seychelles or "Sierra Leone" or "Sri Lanka" or Ceylon or

"Solomon Islands" or Somalia or Sudan or Suriname or Surinam or Swaziland or Syria or Tajikistan or Tadzhikistan or Tadjikistan or Tadzhik or Tanzania or Thailand or Togo or "Togolese Republic" or Tonga or Trinidad or Tobago or Tunisia or Turkey or Turkmenistan or Turkmen or Tuvalu or Uganda or Ukraine or Uruguay or Uzbekistan or Uzbek or Vanuatu or "New Hebrides" or Venezuela or Vietnam or "Viet Nam" or "West Bank" or Yemen or Yugoslavia or Zambia or Zimbabwe) OR AB (Afghanistan or Albania or Algeria or Angola or Antigua or Barbuda or Argentina or Armenia or Armenian or Aruba or Azerbaijan or Bangladesh or Benin or Byelarus or Byelorussian or Belarus or Belorussian or Belorussia or Belize or Bhutan or Bolivia or Bosnia or Herzegovina or Hercegovina or Botswana or Brazil or Bulgaria or "Burkina Faso" or "Burkina Fasso" or "Upper Volta" or Burundi or Urundi or Cambodia or "Khmer Republic" or Kampuchea or Cameroon or Cameroons or Cameron or Camerons or "Cape Verde" or "Central African Republic" or Chad or Chile or China or Colombia or Comoros or "Comoro Islands" or Comores or Mayotte or Congo or Zaire or "Costa Rica" or "Cote d'Ivoire" or "Ivory Coast" or Croatia or Cuba or Djibouti or "French Somaliland" or Dominica or "Dominican Republic" or "East Timor" or "East Timur" or "Timor Leste" or Ecuador or Egypt or "United Arab Republic" or "El Salvador" or Eritrea or Ethiopia or Fiji or Gabon or "Gabonese Republic" or Gambia or Gaza or "Georgia Republic" or "Georgian Republic" or Ghana or "Gold Coast" or Grenada or Guatemala or Guinea or Guam or Guiana or Guyana or Haiti or Honduras or India or Maldives or Indonesia or Iran or Iraq or Jamaica or Jordan or Kazakhstan or Kazakh or Kenya or Kiribati or Korea or Kosovo or Kyrgyzstan or Kirghizia or "Kyrgyz Republic" or Kirghiz or Kirgizstan or "Lao PDR" or Laos or Latvia or Lebanon or Lesotho or Basutoland or Liberia or Libya or Lithuania or Macedonia or Madagascar or "Malagasy Republic" or Malaysia or Malaya or Malay or Sabah or Sarawak or Malawi or Mali or "Marshall Islands" or Mauritania or Mauritius or "Agalega Islands" or Mexico or Micronesia or "Middle East" or Moldova or Moldovia or Moldovian or Mongolia or Montenegro or Morocco or Ifni or Mozambique or Myanmar or Myanma or Burma or Namibia or Nepal or "Netherlands Antilles" or "New Caledonia" or Nicaragua or Niger or Nigeria or Oman or Muscat or Pakistan or Palau or Palestine or Panama or Paraguay or Peru or Philippines or Philipines or Phillipines or Phillippines or Romania or Rumania or Roumania or Russia or Russian or Rwanda or Ruanda or "Saint Lucia" or "St Lucia" or "Saint Vincent" or "St Vincent" or Grenadines or Samoa or "Samoan Islands" or "Navigator Island" or "Navigator Islands" or "Sao Tome" or Senegal or Serbia or Montenegro or Seychelles or "Sierra Leone" or "Sri Lanka" or Ceylon or "Solomon Islands" or Somalia or Sudan or Suriname or Surinam or Swaziland or Syria or Tajikistan or Tadzhikistan or Tadjikistan or Tadzhik or Tanzania or Thailand or Togo or "Togolese Republic" or Tonga or Trinidad or Tobago or Tunisia or Turkey or Turkmenistan or Turkmen or Tuvalu or Uganda or Ukraine or Uruguay or Uzbekistan or Uzbek or Vanuatu or "New Hebrides" or Venezuela or Vietnam or "Viet Nam" or "West Bank" or Yemen or Yugoslavia or Zambia or Zimbabwe) OR SU (Afghanistan or Albania or Algeria or Angola or Antigua or Barbuda or Argentina or Armenia or Armenian or Aruba or Azerbaijan or Bangladesh or Benin or Byelarus or Byelorussian or Belarus or Belorussian or Belorussia or Belize or Bhutan or Bolivia or Bosnia or Herzegovina or Hercegovina or Botswana or Brazil or Bulgaria or "Burkina Faso" or "Burkina Fasso" or "Upper Volta" or Burundi or Urundi or Cambodia or "Khmer Republic" or Kampuchea or Cameroon or Cameroons or Cameron or Camerons or "Cape Verde" or "Central African Republic" or Chad or Chile or China or Colombia or Comoros or "Comoro Islands" or Comores or Mayotte or Congo or Zaire or "Costa Rica" or "Cote d'Ivoire" or "Ivory Coast" or Croatia or Cuba or Djibouti or "French Somaliland" or Dominica or "Dominican Republic" or "East Timor" or "East Timur" or "Timor Leste" or Ecuador or Egypt or "United Arab Republic" or "El Salvador" or Eritrea or Ethiopia or Fiji or Gabon or "Gabonese Republic" or Gambia or Gaza or "Georgia Republic" or "Georgian Republic" or Ghana or "Gold Coast" or Grenada or Guatemala or Guinea or Guam or Guiana or Guyana or Haiti or Honduras or India or Maldives or Indonesia or Iran or Iraq or Jamaica or Jordan or Kazakhstan or Kazakh or Kenya or Kiribati or Korea or Kosovo or Kyrgyzstan or Kirghizia or "Kyrgyz Republic" or Kirghiz or Kirgizstan or "Lao PDR" or Laos or Latvia or Lebanon or Lesotho or Basutoland or Liberia or Libya or Lithuania or Macedonia or Madagascar or "Malagasy Republic" or Malaysia or Malaya or Malay or Sabah or Sarawak or Malawi or Mali or "Marshall Islands" or Mauritania or Mauritius or "Agalega Islands" or Mexico or Micronesia or "Middle East"

or Moldova or Moldovia or Moldovian or Mongolia or Montenegro or Morocco or Ifni or Mozambique or Myanmar or Myanma or Burma or Namibia or Nepal or "Netherlands Antilles" or "New Caledonia" or Nicaragua or Niger or Nigeria or Oman or Muscat or Pakistan or Palau or Palestine or Panama or Paraguay or Peru or Philippines or Philipines or Phillipines or Phillippines or Romania or Rumania or Roumania or Russia or Russian or Rwanda or Ruanda or "Saint Lucia" or "St Lucia" or "Saint Vincent" or "St Vincent" or Grenadines or Samoa or "Samoan Islands" or "Navigator Island" or "Navigator Islands" or "Sao Tome" or Senegal or Serbia or Montenegro or Seychelles or "Sierra Leone" or "Sri Lanka" or Ceylon or "Solomon Islands" or Somalia or Sudan or Suriname or Surinam or Swaziland or Syria or Tajikistan or Tadzhikistan or Tadjikistan or Tadzhik or Tanzania or Thailand or Togo or "Togolese Republic" or Tonga or Trinidad or Tobago or Tunisia or Turkey or Turkmenistan or Turkmen or Tuvalu or Uganda or Ukraine or Uruguay or Uzbekistan or Uzbek or Vanuatu or "New Hebrides" or Venezuela or Vietnam or "Viet Nam" or "West Bank" or Yemen or Yugoslavia or Zambia or Zimbabwe)

14,848,010

S1 TI(immuniz* or immunis* or vaccin* or inoculat* or innoculat* or immunotherap* or prophyla*) OR AB(immuniz* or immunis* or vaccin* or inoculat* or innoculat* or immunotherap* or prophyla*) OR SU(immuniz* or immunis* or vaccin* or inoculat* or innoculat* or immunotherap* or prophyla*)

2,006,154

8. Scopus – Searched 19th May 2019

(TITLE-ABS-KEY (immuniz* OR immunis* OR vaccin* OR inoculat* OR innoculat* OR immunotherap* OR prophyla*))

AND

((TITLE-ABS-KEY (afghanistan OR albania OR algeria OR angola OR antigua OR barbuda OR argentina OR armenia OR armenian OR aruba OR azerbaijan OR bangladesh OR benin OR byelarus OR byelorussian OR belarus OR belorussian OR belorussia OR belize OR bhutan OR bolivia OR bosnia OR herzegovina OR hercegovina OR botswana OR brazil OR bulgaria OR "Burkina Faso" OR "Burkina Fasso" OR "Upper Volta" OR burundi OR urundi OR cambodia OR "Khmer Republic" OR kampuchea OR cameroon OR cameroons OR cameron OR camerons OR "Cape Verde" OR "Central African Republic" OR chad OR chile OR china OR colombia OR comoros OR "Comoro Islands" OR comores OR mayotte OR congo OR zaire OR "Costa Rica" OR "Cote d'Ivoire" OR "Ivory Coast" OR croatia OR cuba OR djibouti OR "French Somaliland" OR dominica OR "Dominican Republic" OR "East Timor" OR "East Timur" OR "Timor Leste" OR ecuador OR egypt OR "United Arab Republic" OR "El Salvador" OR eritrea OR ethiopia OR fiji OR gabon OR "Gabonese Republic" OR gambia OR gaza OR "Georgia Republic" OR "Georgian Republic" OR ghana OR "Gold Coast" OR grenada OR guatemala OR guinea OR guam OR guiana OR guyana OR haiti OR honduras OR india OR maldives OR indonesia OR iran OR iraq OR jamaica OR jordan OR kazakhstan OR kazakh OR kenya OR kiribati OR korea OR kosovo OR kyrgyzstan OR kirghizia OR "Kyrgyz Republic" OR kirghiz OR kirgizstan OR "Lao PDR" OR laos OR latvia OR lebanon OR lesotho OR basutoland OR liberia OR libya OR lithuania OR macedonia OR madagascar OR "Malagasy Republic" OR malaysia OR malaya OR malay OR sabah OR sarawak OR malawi OR mali OR "Marshall Islands" OR mauritania OR mauritius OR "Agalega Islands" OR mexico OR micronesia OR "Middle East" OR moldova OR moldovia OR moldovian OR mongolia OR montenegro OR morocco OR ifni OR mozambique OR myanmar OR myanma OR burma OR namibia OR nepal OR "Netherlands Antilles" OR "New Caledonia" OR nicaragua OR niger OR nigeria OR oman OR

muscat OR pakistan OR palau OR palestine OR panama OR paraguay OR peru OR philippines OR philipines OR phillipines OR phillippines OR romania OR rumania OR roumania OR russia OR russian OR rwanda OR ruanda OR "Saint Lucia" OR "St Lucia" OR "Saint Vincent" OR "St Vincent" OR grenadines OR samoa OR "Samoan Islands" OR "Navigator Island" OR "Navigator Islands" OR "Sao Tome" OR senegal OR serbia OR montenegro OR seychelles OR "Sierra Leone" OR "Sri Lanka" OR ceylon OR "Solomon Islands" OR somalia OR sudan OR suriname OR surinam OR swaziland OR syria OR tajikistan OR tadzhikistan OR tadjikistan OR tadzhik OR tanzania OR thailand OR togo OR "Togolese Republic" OR tonga OR trinidad OR tobago OR tunisia OR turkey OR turkmenistan OR turkmen OR tuvalu OR uganda OR ukraine OR uruguay OR uzbekistan OR uzbek OR vanuatu OR "New Hebrides" OR venezuela OR vietnam OR "Viet Nam" OR "West Bank" OR yemen OR yugoslavia OR zambia OR zimbabwe)) OR (TITLE-ABS-KEY (africa OR asia OR caribbean OR "West Indies" OR "South America" OR "Latin America" OR "Central America")) OR (TITLE-ABS-KEY ((developing OR "less* developed" OR "least developed" OR "under developed" OR underdeveloped OR "middle income" OR "low* income" OR underserved OR "under served" OR deprived OR poor* OR "resource limited" OR "resource constrained") W/1 (countr* OR nation? OR population? OR world OR state*))) OR (TITLE-ABS-KEY ((developing OR "less* developed" OR "under developed" OR underdeveloped OR "middle income" OR "low* income") W/1 (economy OR economies))) OR (TITLE-ABS-KEY (low* W/1 (gdp OR gnp OR "gross domestic" OR "gross national" OR gni))) OR (TITLE-ABS-KEY (low W/3 middle W/3 countr*)) OR (TITLE-ABS-KEY (lmic OR lmics OR "third world" OR "lami countr*")) OR (TITLE-ABS-KEY ("transitional countr*" OR "emerging econom*" OR "global south")))

AND

(TITLE-ABS-KEY (child* OR infant* OR newborn* OR neonat* OR "neo nat*" OR prenatal OR "pre natal" OR "ante natal" OR antenatal OR baby OR babies OR toddler* OR preschool* OR parent* OR mother* OR father* OR maternal OR paternal))

AND

((TITLE-ABS-KEY (random* OR experiment* OR (match* W/2 (propensity OR coarsened OR covariate)) OR "propensity score" OR ("difference in difference*" OR "difference-in-difference*" OR "differences in difference*" OR "differences-in-difference*" OR "double difference*") OR ("quasi-experimental" OR "quasi experimental" OR "quasi-experiment" OR "quasi experiment") OR ((estimator OR counterfactual) AND evaluation*) OR "instrumental variable*" OR (iv W/2 (estimation OR approach)) OR "regression discontinuity" OR "time series" OR "segment* regression")) OR (TITLE-ABS-KEY (cost-effective* OR cost-benefit)) OR (TITLE-ABS-KEY ("life year" OR "life years" OR qaly* OR daly*)) OR (TITLE-ABS-KEY ((economic* OR cost*) W/6 (mortality OR death* OR markov))) OR (TITLE-ABS-KEY ("cost minimi*" OR "cost-utilit*" OR "economic evaluation*" OR "economic review*" OR "cost outcome" OR "cost analys*" OR "economic analys*" OR "budget* impact analys*")) OR (TITLE-ABS-KEY ("systematic review" OR "literature review" OR meta-analy* OR metaanaly* OR "meta analy*")))

AND

(LIMIT-TO (SUBJAREA , "MEDI") OR LIMIT-TO (SUBJAREA , "SOCI") OR LIMIT-TO (SUBJAREA , "NURS") OR LIMIT-TO (SUBJAREA , "MULT") OR LIMIT-TO (SUBJAREA , "ECON") OR LIMIT-TO (SUBJAREA , "HEAL") OR LIMIT-TO (SUBJAREA , "PSYC") OR LIMIT-TO (SUBJAREA , "DECI")) - 8214 hits

9. **Popline – Searched 19th May 2019**

ALL FIELDS: (immuniz* OR immunis* OR vaccin* OR inoculat* OR innoculat* OR immunotherap* OR prophyla*)
AND
ALL FIELDS: (child* OR infant* OR newborn* OR neonat* OR "neo nat*" OR prenatal OR "pre natal" OR "ante natal" OR antenatal OR baby OR babies OR toddler* OR preschool* OR parent* OR mother* OR father* OR maternal OR paternal)
AND
ALL FIELDS: (random* OR experiment* OR (match* AND (propensity OR coarsened OR covariate)) OR "propensity score" OR ("difference in difference*" OR "difference-in-difference*" OR "differences in difference*" OR "differences-in-difference*" OR "double difference*") OR ("quasi-experimental" OR "quasi experimental" OR "quasi-experiment" OR "quasi experiment") OR ((estimator OR counterfactual) AND evaluation*) OR "instrumental variable*" OR (iv AND (estimation OR approach)) OR "regression discontinuity" OR "time series" OR "segment* regression")
Result: 1134 hits

10. **WHO Global Health Index – Searched 20th May 2019**

(immuniz* or immunis* or vaccin* or inoculat* or innoculat* or immunotherap* or prophyla*) AND (child* OR infant* OR newborn* OR neonat* OR "neo nat*" OR prenatal OR "pre natal" OR "ante natal" OR antenatal OR baby OR babies OR toddler* OR preschool* OR parent* OR mother* OR father* OR maternal OR paternal) AND (random* OR experiment* OR (match* AND (propensity OR coarsened OR covariate)) OR "propensity score" OR ("difference in difference*" OR "difference-in-difference*" OR "differences in difference*" OR "differences-in-difference*" OR "double difference*") OR ("quasi-experimental" OR "quasi experimental" OR "quasi-experiment" OR "quasi experiment") OR ((estimator OR counterfactual) AND evaluation*) OR "instrumental variable*" OR (iv AND (estimation OR approach)) OR "regression discontinuity" OR "time series" OR "segment* regression") – 2833 hits

11. **Epistemonikos – Searched 20th May 2019**

(title:((immuniz* OR immunis* OR vaccin* OR inoculat* OR innoculat* OR immunotherap* OR prophyla*) AND (child* OR infant* OR newborn* OR neonat* OR "neo nat*" OR prenatal OR "pre natal" OR "ante natal" OR antenatal OR baby OR babies OR toddler* OR preschool* OR parent* OR mother* OR father* OR maternal OR paternal) AND (random* OR experiment* OR (match* AND (propensity OR coarsened OR covariate)) OR "propensity score" OR ("difference in difference*" OR "difference-in-difference*" OR "differences in difference*" OR "differences-in-difference*" OR "double difference*") OR ("quasi-experimental" OR "quasi experimental" OR "quasi-experiment" OR "quasi experiment") OR ((estimator OR counterfactual) AND evaluation*) OR "instrumental variable*" OR (iv AND (estimation OR approach)) OR "regression discontinuity" OR "time series" OR "segment* regression")) OR abstract:((immuniz* OR immunis* OR vaccin* OR inoculat* OR innoculat* OR immunotherap* OR prophyla*) AND (child* OR infant* OR newborn* OR neonat* OR "neo nat*" OR prenatal OR "pre natal" OR "ante natal" OR antenatal OR baby OR babies OR toddler* OR preschool* OR parent* OR mother* OR father* OR maternal OR paternal) AND (random* OR experiment* OR (match* AND (propensity OR coarsened OR covariate)) OR "propensity score" OR ("difference in difference*" OR "difference-in-difference*" OR "differences in difference*" OR "differences-in-difference*" OR "double difference*") OR ("quasi-experimental" OR "quasi experimental" OR "quasi-experiment" OR "quasi experiment") OR ((estimator OR counterfactual) AND evaluation*) OR "instrumental variable*" OR (iv AND (estimation OR approach)) OR "regression discontinuity" OR "time series" OR "segment* regression"))) – 3041 hits

12. Pascal-Francis (Updated to 2015) – Searched 20th May 2019

1. (((immuniz* OR immunis* OR vaccin* OR inoculat* OR innoculat* OR immunotherap* OR prophyla*) AND (child* OR infant* OR newborn* OR neonat* OR "neo nat*" OR prenatal OR "pre natal" OR "ante natal" OR antenatal OR baby OR babies OR toddler* OR preschool* OR parent* OR mother* OR father* OR maternal OR paternal) AND (random* OR experiment* OR (match* AND (propensity OR coarsened OR covariate)) OR "propensity score" OR ("difference in difference*" OR "difference-in-difference*" OR "differences in difference*" OR "differences-in-difference*" OR "double difference*") OR ("quasi-experimental" OR "quasi experimental" OR "quasi-experiment" OR "quasi experiment") OR ((estimator OR counterfactual) AND evaluation*) OR "instrumental variable*" OR (iv AND (estimation OR approach)) OR "regression discontinuity" OR "time series" OR "segment* regression"))) AND (((low* AND (gdp OR gnp OR "gross domestic" OR "gross national" OR gni)) OR (low AND middle AND countr*) OR lmic OR lmics OR "third world" OR "lami countr*" OR "transitional countr*" OR "emerging econom*" OR "global south")) – 1 hit

2. (((immuniz* OR immunis* OR vaccin* OR inoculat* OR innoculat* OR immunotherap* OR prophyla*) AND (child* OR infant* OR newborn* OR neonat* OR "neo nat*" OR prenatal OR "pre natal" OR "ante natal" OR antenatal OR baby OR babies OR toddler* OR preschool* OR parent* OR mother* OR father* OR maternal OR paternal) AND (random* OR experiment* OR (match* AND (propensity OR coarsened OR covariate)) OR "propensity score" OR ("difference in difference*" OR "difference-in-difference*" OR "differences in difference*" OR "differences-in-difference*" OR "double difference*") OR ("quasi-experimental" OR "quasi experimental" OR "quasi-experiment" OR "quasi experiment") OR ((estimator OR counterfactual) AND evaluation*) OR "instrumental variable*" OR (iv AND (estimation OR approach)) OR "regression discontinuity" OR "time series" OR "segment* regression"))) AND (((developing OR "less* developed" OR "least developed" OR "under developed" OR underdeveloped OR "middle income" OR "low* income" OR underserved OR "under served" OR deprived OR poor* OR "resource limited" OR "resource constrained") AND (countr* OR nation? OR population? OR world OR state* OR economy OR economies))) – 74 hits

3. (((immuniz* OR immunis* OR vaccin* OR inoculat* OR innoculat* OR immunotherap* OR prophyla*) AND (child* OR infant* OR newborn* OR neonat* OR "neo nat*" OR prenatal OR "pre natal" OR "ante natal" OR antenatal OR baby OR babies OR toddler* OR preschool* OR parent* OR mother* OR father* OR maternal OR paternal) AND (random* OR experiment* OR (match* AND (propensity OR coarsened OR covariate)) OR "propensity score" OR ("difference in difference*" OR "difference-in-difference*" OR "differences in difference*" OR "differences-in-difference*" OR "double difference*") OR ("quasi-experimental" OR "quasi experimental" OR "quasi-experiment" OR "quasi experiment") OR ((estimator OR counterfactual) AND evaluation*) OR "instrumental variable*" OR (iv AND (estimation OR approach)) OR "regression discontinuity" OR "time series" OR "segment* regression"))) AND ((africa OR asia OR caribbean OR "West Indies" OR "South America" OR "Latin America" OR "Central America")) – 285 hits

((immuniz* OR immunis* OR vaccin* OR inoculat* OR innoculat* OR immunotherap* OR prophyla*) AND (child* OR infant* OR newborn* OR neonat* OR "neo nat*" OR prenatal OR "pre natal" OR "ante natal" OR antenatal OR baby OR babies OR toddler* OR preschool* OR parent* OR mother* OR father* OR maternal OR paternal) AND (random* OR experiment* OR (match* AND (propensity OR coarsened OR covariate)) OR "propensity score" OR ("difference in difference*" OR "difference-in-difference*" OR "differences in difference*" OR "differences-in-difference*" OR "double difference*") OR ("quasi-experimental" OR "quasi experimental" OR "quasi-experiment" OR "quasi experiment") OR ((estimator OR counterfactual) AND evaluation*) OR "instrumental variable*" OR (iv AND (estimation OR approach)) OR "regression discontinuity" OR "time series" OR "segment* regression")) AND (afghanistan OR albania OR algeria OR angola OR antigua OR barbuda OR argentina OR armenia OR armenian OR aruba OR azerbaijan OR bangladesh OR benin OR byelarus OR byelorussian OR belarus OR belorussian OR belorussia OR belize OR bhutan OR bolivia OR bosnia OR herzegovina OR hercegovina OR botswana OR brazil OR bulgaria OR "Burkina Faso" OR "Burkina Fasso" OR "Upper Volta" OR burundi OR urundi OR cambodia OR "Khmer

Republic" OR kampuchea OR cameroon OR cameroons OR cameron OR camerons OR "Cape Verde" OR "Central African Republic" OR chad OR chile OR china OR colombia OR comoros OR "Comoro Islands" OR comores OR mayotte OR congo OR zaire OR "Costa Rica" OR "Cote d'Ivoire" OR "Ivory Coast" OR croatia OR cuba OR djibouti OR "French Somaliland" OR dominica OR "Dominican Republic" OR "East Timor" OR "East Timur" OR "Timor Leste" OR ecuador OR egypt OR "United Arab Republic" OR "El Salvador" OR eritrea OR ethiopia OR fiji OR gabon OR "Gabonese Republic" OR gambia OR gaza OR "Georgia Republic" OR "Georgian Republic" OR ghana OR "Gold Coast" OR grenada OR guatemala OR guinea OR guam OR guiana OR guyana OR haiti OR honduras OR india OR maldives OR indonesia OR iran OR iraq OR jamaica OR jordan OR kazakhstan OR kazakh OR kenya OR kiribati OR korea OR kosovo OR kyrgyzstan OR kirghizia OR "Kyrgyz Republic" OR kirghiz OR kirgizstan OR "Lao PDR" OR laos OR latvia OR lebanon OR lesotho OR basutoland OR liberia OR libya OR lithuania OR macedonia OR madagascar OR "Malagasy Republic" OR malaysia OR malaya OR malay OR sabah OR sarawak OR malawi OR mali OR "Marshall Islands" OR mauritania OR mauritius OR "Agalega Islands" OR mexico OR micronesia OR "Middle East" OR moldova OR moldovia OR moldovian OR mongolia OR montenegro OR morocco OR ifni OR mozambique OR myanmar OR myanma OR burma OR namibia OR nepal OR "Netherlands Antilles" OR "New Caledonia" OR nicaragua OR niger OR nigeria OR oman OR muscat OR pakistan OR palau OR palestine OR panama OR paraguay OR peru OR philippines OR philipines OR phillipines OR phillippines OR romania OR rumania OR roumania OR russia OR russian OR rwanda OR ruanda OR "Saint Lucia" OR "St Lucia" OR "Saint Vincent" OR "St Vincent" OR grenadines OR samoa OR "Samoan Islands" OR "Navigator Island" OR "Navigator Islands" OR "Sao Tome" OR senegal OR serbia OR montenegro OR seychelles OR "Sierra Leone" OR "Sri Lanka" OR ceylon OR "Solomon Islands" OR somalia OR sudan OR suriname OR surinam OR swaziland OR syria OR tajikistan OR tadzhikistan OR tadjikistan OR tadzhik OR tanzania OR thailand OR togo OR "Togolese Republic" OR tonga OR trinidad OR tobago OR tunisia OR turkey OR turkmenistan OR turkmen OR tuvalu OR uganda OR ukraine OR uruguay OR uzbekistan OR uzbek OR vanuatu OR "New Hebrides" OR venezuela OR vietnam OR "Viet Nam" OR "West Bank" OR yemen OR yugoslavia OR zambia OR zimbabwe) – 590 hits

B.2 Grey literature searches

To identify relevant grey literature, we will search the following databases and websites:

- Campbell Library
- Google Scholar
- Open-Grey
- Grey Literature Report
- Social Science Research Network (SSRN)
- Eldis
- GAVI
- Innovations for Poverty Action (IPA)
- Abdul Latif Jameel Poverty Action Lab (J-PAL)
- 3ie Impact Evaluation Repository
- 3ie Systematic Review Repository
- Registry of International Development Impact Evaluations (RIDIE)
- Global Development Network
- World Bank Development Impact Evaluation (DIME) and Impact Evaluation Policy Papers
- Inter-American Development Bank

- Center for Global Development
- Center for Effective Global Action (CEGA)
- DFID Research for Development (R4D)
- USAID

B.3 Data extraction template

Coding field	Description/coding instructions
Study title	Use only the English version of the publication's main title. If paper is not written in English and has the title translated, use the translated version of the title. If the publication does not provide an English version, include the title in its original language.
Foreign title	When publication is not written in English, code the original title using original accents and special characters.
Language	Select **full text** language that applies: English, French, Spanish, Portuguese
Authors	Enter all study authors
Publication type	Whether the study is a journal article, working paper, report, etc.
Publication source	The journal, working paper series, or institution publishing the study
Year	Input the year when the print version of the study was published. If the publication is online-only, then input the date the publication appeared online.
Country or countries	The country or countries where the study was conducted
Equity focus	How does this study consider gender and/or equity?
Equity dimension	Which dimension(s) of gender and/or equity does the intervention target?
Equity description	Open answer - provide a description of how the study considers gender and equity, and for which population to corroborate answers above (include page numbers where relevant)
Adverse effects	Input any potential adverse effects of the intervention that the study reports on. If study does not report on any potential adverse effects, code as “Not applicable”.
Evaluation design	The general evaluation design (i.e., experimental or quasi-experimental)
Evaluation method	The specific method used to evaluate impact (mainly applicable to quasi-experimental studies—regression discontinuity, instrumental variables, etc.)
Mixed methods	Select YES if study includes quantitative and qualitative analyses, otherwise select NO.
Unit of observation	The unit(s) of observation/analysis used in the study, e.g., individual, household, village, etc.
Programme name	Input the name of the project/programme being evaluated (if any)

Implementing agency	Input the name of the agency or agencies implementing the programme
Programme funding agency	Input the name of the agency or agencies funding the programme (note: this is not the same as organizations that fund the research of the evaluation)
Research funding agency	Input the name of the agency or agencies funding the research (note: this is not the same as organizations that fund the programme)
Intervention A category	Select the framework category for the first intervention
Intervention A description	Briefly describe the activities that comprise the intervention.
Add rows for additional interventions as needed.	
Outcome 1 category	Select the framework category for the first outcome.
Outcome 1 description	Briefly describe the first outcome.
Add rows for additional outcomes as needed.	
Hard-to-reach	Does the study report on differential impacts for hard-to-reach populations?
Sex	Does the study report on differential impacts by gender?
Socioeconomic status	Does the study report on differential impacts by socioeconomic status (e.g., wealth quantile, caste)?
Maternal education	Does the study report on differential impacts by maternal education level?
Cost analysis	Does the study include cost or cost-effectiveness analysis?
Information sources	Does the study include data on different information sources about vaccination?
Adverse effects	Does the study provide data about potential adverse effects of the intervention?
Multi-component	Is the intervention multi-component?
Add rows for additional outcomes as needed.	

B.4 SR critical appraisal tool

Checklist for making judgements about how much confidence to place in a systematic review of effects (adapted version of SURE checklist)[2]

**Assessed by**:
**Date**:

Overall Notes. When the primary publication does not provide the information needed to appraise, information in other version of the publication can be used (document additional source in the appraisal). Study protocols cannot be used as a source, as the plans described in the protocols might not have been implemented.

We provide authors with the completed checklist and they can provide additional information.

Section A: *Methods used to identify, include and critically appraise studies*

**A.1 Were the criteria used for deciding which studies to include in the review reported?**<br><br>Did the authors specify:<br>☐ Types of studies<br>☐ Participants/ settings/ population<br>☐ Intervention(s)<br>☐ Outcome(s)<br><br>*Note.* This information cannot be determined by looking at the types of studies included, because some eligible populations, designs, interventions, and outcomes might not have been examined in the studies.	☐ Yes<br>☐ Partially<br>☐ No<br><br>*Coding guide - check the answers above*<br>*YES: All four should be yes*<br>*NO: All four should be no*<br>*PARTIALLY: Any other*
Documentation/Comments (note important limitations or uncertainty)	

[2] Adapted from Supporting the Use of Research Evidence (SURE) Collaboration. SURE checklist for making judgements about how much confidence to place in a systematic review. In: (SURE Collaboration 2011).

**A.2 Was the search for evidence reasonably comprehensive?**<br>Were the following done:<br>☐ Language bias avoided (no restriction of inclusion based on language)<br>☐ No restriction of inclusion based on publication status<br>☐ Relevant databases searched: at least one database that includes grey/unpublished literature,[3] as well as either: (a) for health, at least two relevant comprehensive subject databases (such as PubMed/MEDLINE, EMBASE and CENTRAL),[4] or (b) for social sciences, at least two relevant comprehensive subject databases (such as IDEAS) and one comprehensive general database (such as EconLit, PsychInfo, Scopus)<br>☐ Reference lists in included articles checked<br>☐ Authors/experts contacted<br><br>*Notes*. When authors do not mention limitation on language or publication status, code *Yes*. The use of “published” often simply means released (e.g, “studies published between 1990 – 2010”) and not necessarily that studies were excluded based on publication status; do not code No simply because the authors use “published” in this way. When authors do not mention that reference lists were searched or experts contacted, code *No*. If authors were only contacted for study results data, Code *No*. Checking reference lists of review articles does not fully meet A2.4 requirement (code *Partially*) but is a mitigating factor.	☐ Yes<br>☐ Partially<br>☐ No<br>☐ Can’t tell<br><br>*Coding guide - check the answers above:*<br>*YES: All five should be yes*<br>*PARTIALLY: Relevant databases and reference lists are both reported*<br>*NO: Any other*
Documentation/Comments (note important limitations or uncertainty)	

[3] Grey literature typically means research that is not published in sources such as books or journal articles. The following databases include grey literature: Academic Search Complete (includes many conference proceedings), CAB Abstracts, search conducted using CADATH checklist, clinicaltrials.gov, Cochrane Central Register of Controlled Trials (CENTRAL), Cochrane Library, Embase (includes 3.6m+ conference abstracts), Google, Google Scholar, Healthcare Management Information Consortium (HMIC), IDEAS/RePEc, National Technical Information Service (NTIS), OpenSIGLE/OpenGrey, PsycEXTRA, Scopus (includes ~10m conference papers). If you identify additional sources, please add to the list. Searching websites of relevant governmental agencies and non-governmental organizations can also identify grey literature. Note that MEDLINE/PubMed, a comprehensive data base of journals, does not include grey literature: “For indexing in MEDLINE, NLM currently selects publications that it considers to be journals.”; see also Citrome L. Beyond PubMed: Searching the "Grey Literature" for Clinical Trial Results. Innov Clin Neurosci. 2014;11(7-8):42-46.
[4] If the search is for studies in China, the CNKI (China National Knowledge infrastructure) is acceptable as a comprehensive database.

**A.3 Does the review cover an appropriate time period?**<br><br>*Is the search period comprehensive enough that relevant literature is unlikely to be omitted?*<br><br>*Note.* If the authors do not report the search period, check the publication date of the earliest included study. If the study was published before 1990 this can be coded *Yes*.	☐ Yes<br>☐ Can't tell (only use if no information about time period for search)<br>☐ No<br>☐Unsure<br><br>*Coding guide:*<br>*YES: Generally this means searching the literature at least back to 1990*<br>*NO: Generally if the search does not go back to 1990*<br>*CAN'T TELL: No information about time period for search*<br><br>*Note: With reference to the above – there may be important reasons for adopting different dates for the search, e.g. depending on the intervention. If you think there are limitations with the timeframe adopted for the search which have not been noted and justified by the authors, you should code this item as a NO and specify your reason for doing so in the comment box below. Older reviews should not be downgraded, but the fact that the search was conducted some time ago should be noted in the quality assessment. Always report the time period for the search in the comment box.*
Documentation/Comments (note important limitations or uncertainty)	

**A.4 Was bias in the selection of articles avoided?**<br><br>Did the authors specify:<br>☐ Independent screening of full text by at least 2 reviewers<br>☐ List of included studies provided<br>☐ List of excluded studies provided<br><br>*Notes*. Independent screening means that both screeners screened all full-text without knowing what the other screener decided (that is, one screener and one verifier does not meet criterion). If the authors note two screeners and do not use the word “independent” but mention a third reconciler to resolve differences, assume independence. When authors do not mention whether independent screening was conducted by at least two reviewers, code *No*. The list of excluded studies does not need to include studies that were abstract screened out as ineligible. Because journals often have word count limits, reviews published in journals do not need to have a list of excluded studies and are coded *Not Applicable.*	☐ Yes<br>☐ Partially<br>☐ No<br><br>*Coding guide:*<br>*YES: All three should be yes, although reviews published in journals are unlikely to have a list of excluded studies (due to limits on word count) and the review should not be penalised for this.*<br>*PARTIALLY: Independent screening and list of included studies provided are both reported*<br>*NO: All other. If list of included studies provided, but the authors do not report whether or not the screening has been done by 2 reviewers review is downgraded to NO.*
Documentation/Comments (note important limitations or uncertainty)	

**A.5 Did the authors use appropriate criteria to assess the quality and risk of bias in analysing the studies that are included?**[5]<br>☐ The criteria used for assessing the quality/ risk of bias were reported<br>☐ A table or summary of the assessment of each included study for each criterion was reported<br>☐ Sensible criteria were used that focus on the quality/ risk of bias (and not other qualities of the studies, such as precision or applicability/external validity). "Sensible" is defined as a recognised quality appraisal tool/ checklist, or similar tool which assesses bias in included studies Please see footnotes for details of the main types of bias such a tool should assess.<br><br>*Notes.* Identified tools with sensible criteria include: Academy of Nutrition and Dietetics Quality Criteria Checklist, Child Health Epidemiology Reference Group (CHERG) study design & quality standards, Cochrane Handbook, The Delphi List, Effective Public Health Practice Project (EPHPP) Quality Assessment Tool, Grading of Recommendations Assessment, Development and Evaluation (GRADE) RoB criteria, Guide to Community Preventative Services Study Quality tool, Joanna Briggs Institute Checklists for RCT/QED, National Institutes of Health's Quality Assessment Tool for Controlled Intervention Studies (sometimes labelled NHLBI tool). For case-control studies and cohort studies, the Newcastle-Ottawa Scale uses sensible criteria that are focused on risk of bias as does Methodological Index for Non-Randomized Studies (MINORS). Note that these designs typically are not as rigorous as RCTs or even QEDs.	☐ Yes<br>☐ Partially<br>☐ No<br><br>*Coding guide:*<br>*YES: All three should be yes*<br>*PARTIALLY: The first and third criteria should be reported. If the authors report the criteria for assessing risk of bias and report a summary of this assessment for each criterion, but the criteria may be only partially sensible (e.g. do not address all possible risks of bias, but do address some), we downgrade to PARTIALLY.*<br>*NO: Any other*
Documentation/Comments (note important limitations or uncertainty)	

[5] **Risk of bias** is the extent to which bias may be responsible for the findings of a study. **Bias** is a systematic error or deviation from the truth in results or inferences. In studies of the effects of social, economic and health care interventions, the main types of bias arise from systematic differences in the groups that are compared (selection bias), the intervention that is provided, or exposure to other factors apart from the intervention of interest (performance bias/contamination), withdrawals or exclusions of people entered into a study (attrition bias) or how outcomes are assessed (detection bias) and reported (reporting bias). Reviews of social science studies may be particularly affected by reporting bias, where a biased subset of all the relevant data and analyses is presented. Assessments of the risk of bias are sometimes also referred to as assessments of the **validity** or **quality** of a study. **Validity** is the extent to which a result (of a measurement or study) is likely to be true. **Quality** is a vague notion of the strength or validity of a study, often indicating the extent of control over bias.

**A.6 Overall – how much confidence do you have in the methods used to identify, include and critically appraise studies?**<br>*Summary assessment score A relates to the 5 questions above.*<br>*High confidence applicable when the answers to the questions in section A are all assessed as 'yes'*<br>*Low confidence applicable when any of the following are assessed as 'NO' above: not reporting explicit selection criteria (A1), not conducting reasonably comprehensive search (A2), not avoiding bias in selection of articles (A4 , not assessing the risk of bias in included studies (A5)*<br>*Medium confidence applicable for any other – i.e. section A3 is assessed as 'NO' or can't tell and remaining sections are assessed as 'partially' or 'can't tell'*	☐ **Low confidence** (limitations are important enough that the results of the review are not reliable)<br>☐ **Medium confidence** (limitations are important enough that it would be worthwhile to search for another systematic review and to interpret the results of this review cautiously, if a better review cannot be found)<br>☐ **High confidence** (only minor limitations)
Documentation/Comments (note important limitations or uncertainty)	

*Section B***:** *Methods used to analyse the findings*

**B.1 Were the characteristics and results of the included studies reliably reported?**<br>Was there:<br>☐ Independent data extraction by at least 2 reviewers<br>☐ A table or summary of the characteristics of the participants, interventions and outcomes for the included studies<br>☐ A table or summary of the results of all the included studies<br><br>*Notes*. Independent extraction means that both extractors extracted all data without knowing what the other extractor decided (that is, one extractor and one verifier does not meet criterion). If the authors note two reviewers and do not use the word "independent" but mention a third reconciler to resolve differences, assume independence. When authors do not mention whether independent extraction was conducted by at least two reviewers, code *No*. Forest plots are an appropriate summary of the results, as is reporting summarizes the findings by outcome domain.	☐ Yes<br>☐ No<br>☐ Partially<br>☐ Not applicable (e.g. no included studies)<br><br>*Coding guide:*<br>*YES: All three should be yes*<br>*PARTIALLY: Criteria one and three are yes, but some information is lacking on second criteria.*<br>*No: None of these are reported. If the review does not report whether data was independently extracted by 2 reviewers (possibly a reporting error), we downgrade to NO.*<br>*NOT APPLICABLE: if no studies/no data*
Documentation/Comments (note important limitations or uncertainty)	

**B.2 Are the methods used by the review authors to analyse the findings of the included studies clear, including methods for calculating effect sizes if applicable?**<br><br>*Note*. An example of acceptable reporting: “fixed effects meta-analysis, with standardized mean differences for continuous outcomes and response ratios for dichotomous outcomes”	☐ Yes<br>☐ Partially<br>☐ No<br>☐ Not applicable (e.g. no studies or no data)<br><br>*Coding guide:*<br>*YES: Methods used clearly reported. If it is clear that the authors use narrative synthesis, they don't need to say this explicitly.*<br>*PARTIALLY: Some reporting on methods but lack of clarity*<br>*NO: Nothing reported on methods*<br>*NOT APPLICABLE: if no studies/no data*
Documentation/Comments (note important limitations or uncertainty)	
**B.3 Did the review describe the extent of heterogeneity?**<br><br>☐ Did the review ensure that included studies were similar enough that it made sense to combine them, sensibly divide the included studies into homogeneous groups, or sensibly conclude that it did not make sense to combine or group the included studies?<br>☐ Did the review discuss the extent to which there were important differences in the results of the included studies?<br>☐ If a meta-analysis was done, was the I^2, chi square test for heterogeneity or other appropriate statistic reported? If no statistical test was reported, is a qualitative justification made for the use of random effects?<br><br>*Notes.* Code B3.1 *No* if analyses includes studies with implausibly different interventions, comparisons, or populations. If a narrative analysis, the authors need to have a rationale for why studies were not combined (such as interventions were too different) or Code B3.1 as *No*. For meta-analyses, reporting a metric for heterogeneity is sufficient for B3.2. For non-meta-analysis, mentioning heterogeneity in results is enough (for example, The impacts varied from X to Y or Study A found X and Study B found Y).	☐ Yes<br>☐ Partially<br>☐ No<br>☐ Not applicable (e.g. no studies or no data)<br><br>*Coding guide:*<br>*YES: First two should be yes, and third category should be yes if applicable should be yes*<br>*PARTIALLY: The first category is yes*<br>*NO: Any other*<br>*NOT APPLICABLE: if no studies/no data*
Documentation/Comments (note important limitations or uncertainty)	

**B.4 Were the findings of the relevant studies combined (or not combined) appropriately relative to the <u>primary question</u> the review addresses and the available data?**<br><br>How was the data analysis done?<br>☐ Descriptive only<br>☐ Vote counting based on direction of effect<br>☐ Vote counting based on statistical significance<br>☐ Description of range of effect sizes<br>☐ Meta-analysis<br>☐ Meta-regression<br>☐ Other: specify<br>☐ Not applicable (e.g. no studies or no data)<br>How were the studies weighted in the analysis?<br>☐ Equal weights (this is what is done when vote counting is used)<br>☐ By quality or study design (this is rarely done)<br>☐ Inverse variance (this is what is typically done in a meta-analysis)<br>☐ Number of participants (sample size)<br>☐ Other: specify<br>☐ Not clear<br>☐ Not applicable (e.g. no studies or no data)<br>Did the review address unit of analysis errors?<br>☐ Yes - took clustering into account in the analysis (e.g. used intra-cluster correlation coefficient)<br>☐ No, but acknowledged problem of unit of analysis errors<br>☐ No mention of issue<br>☐ Not applicable - no clustered trials or studies included	☐ Yes<br>☐ Partially<br>☐ No<br>☐ Not applicable (e.g. no studies or no data)<br>☐ Can't tell<br><br>*Coding guide:*<br>*YES: If appropriate table, graph or meta-analysis (or descriptive where meta-analysis not possible and authors report magnitude of effects for all included studies) AND appropriate weights AND unit of analysis errors addressed (if appropriate).*<br>*PARTIALLY: If appropriate table, graph or meta-analysis AND appropriate weights AND unit of analysis errors not mentioned or not addressed (and should have been).*<br>*NO: If descriptive OR vote counting (where quantitative analyses would have been possible) OR inappropriate reporting of table, graph or meta-analyses.*<br>*NOT APPLICABLE: if no studies/no data*<br>*CAN'T TELL: if unsure (note reasons in comments below)*
Documentation/Comments (note important limitations or uncertainty)	

**B. 5 Does the review report evidence appropriately?**<br><br>☐ The review makes clear which evidence is subject to low risk of bias in assessing causality (attribution of outcomes to intervention), and which is likely to be biased, and does so appropriately<br>☐ Where studies of differing risk of bias are included, results are reported and analysed separately by risk of bias status<br><br>*Notes.* Making clear which evidence is subject to low risk of bias can be accomplished in a table listing RoB for each study or by listing RoB for each study on each RoB criterion; that is, if A5.2 is *Yes*, then B5.1 is *Yes* (but the reverse is not true). Reporting only study design is not sufficient to meet B5.1. For B5.2, narrative analysis must group or report by RoB, it is not sufficient to simply report RoB of each study. If the SR does not use sensible criteria to assess RoB, then B5.1 is *No*.<br><br>*Note on reporting evidence and risk of bias:* For reviews of effects of 'large n' interventions, experimental and quasi-experimental designs should be included (if available). For reviews of effects of 'small n' interventions, designs appropriate to attribute changes to the intervention should be included (e.g. pre-post with assessment of confounders).	☐ Yes<br>☐ No<br>☐ Partially<br>☐ Not applicable<br><br>*Coding guide:*<br>*YES: Both criteria should be fulfilled (where applicable)*<br>*NO: Criteria not fulfilled*<br>*PARTIALLY: Only one criteria fulfilled, or when there is limited reporting of quality appraisal (the latter applies only when inclusion criteria for study design are appropriate)*<br>*NOT APPLICABLE: No included studies*
Documentation/Comments (note important limitations or uncertainty)	
**B.6 Did the review examine the extent to which specific factors might explain differences in the results of the included studies?**<br><br>☐ Were factors that the review authors considered as likely explanatory factors clearly described?<br>☐ Was a sensible method used to explore the extent to which key factors explained heterogeneity?<br>☐ Descriptive/textual<br>☐ Graphical<br>☐ Meta-analysis by sub-groups<br>☐ Meta-regression<br>☐ Other	☐ Yes<br>☐ Partially<br>☐ No<br>☐ Not applicable<br><br>*Coding guide:*<br>*YES: Explanatory factors clearly described and appropriate methods used to explore heterogeneity*<br>*PARTIALLY: Explanatory factors described but for meta-analyses, sub-group analysis or meta-regression not reported (when they should have been)*<br>*NO: No description or analysis of likely explanatory factors*<br>*NOT APPLICABLE: e.g. too few studies, no important differences in the results of the included studies, or the included studies were so dissimilar that it would not make sense to explore heterogeneity of the results*
Documentation/Comments (note important limitations or uncertainty)	

**B.7 Overall - how much confidence do you have in the methods used to analyse the findings relative to the primary question addressed in the review?**<br><br>*Summary assessment score B relates to the 5 questions in this section, regarding the analysis.*<br><br>*High confidence applicable when all the answers to the questions in section B are assessed as 'yes'.*<br><br>*Low confidence applicable when any of the following are assessed as 'NO' above: critical characteristics of the included studies not reported (B1), not describing the extent of heterogeneity (B3), combining results inappropriately (B4), reporting evidence inappropriately (B5).*<br><br>*Medium confidence applicable for any other: i.e. the "Partial" option is used for any of the 6 preceding questions or questions and/or B.2 and/ or B.6 are assessed as 'no'.*	☐ **Low confidence** (limitations are important enough that the results of the review are not reliable)<br><br>☐ **Medium confidence** (limitations are important enough that it would be worthwhile to search for another systematic review and to interpret the results of this review cautiously, if a better review cannot be found)<br><br>☐ **High confidence** (only minor limitations)
Documentation/Comments (note important limitations or uncertainty)	

Section C: Overall assessment of the reliability of the review

**C.1 Are there any other aspects of the review not mentioned before which lead you to question the results?**	☐ Additional methodological concerns – only one person reviewing<br>☐ Robustness<br>☐ Interpretation<br>☐ Conflicts of interest (of the review authors or for included studies)<br>☐ Other<br>☐ No other quality issues identified
**C.2 Are there any mitigating factors which should be taken into account in determining the reviews reliability?**	☐ Limitations acknowledged<br>☐ No strong policy conclusions drawn (including in abstract/ summary)<br>☐ Any other factors
Documentation/Comments (note important limitations or uncertainty)	
C.3 **Based on the above assessments of the methods how would you rate the reliability of the review?**<br><br>☐ **Low confidence in conclusions about effects**:<br><br>☐ **Medium confidence in conclusions about effects**:<br><br>☐ **High confidence in conclusions about effects** :<br><br>*Coding guide:*<br>**High confidence in conclusions about effects**: high confidence noted overall for sections A and B, unless moderated by answer to C1<br>**Medium confidence in conclusions about effects**: medium confidence noted overall for sections A or B<br>**Low confidence in conclusions about effects**: low confidence noted overall for sections A or B, unless moderated by answer to C1 or C2.<br><br>Limitations should be summarised above, based on what was noted in Sections A, B and C.	

Appendix C: References – Included studies

Impact evaluations

Aaby, Peter, Adam Roth, Henrik Ravn, Bitiguida Mutna Napirna, Amabelia Rodrigues, Ida Maria Lisse, Lone Stensballe, et al. 2011. "Randomized Trial of BCG Vaccination at Birth to Low-Birth-Weight Children: Beneficial Nonspecific Effects in the Neonatal Period?" *The Journal of Infectious Diseases* 204 (2): 245–52. https://doi.org/10/ds6pbx.

Adamu, Abdu A, Olalekan A Uthman, Muktar A Gadanya, and Charles S Wiysonge. 2019. "Implementation and Evaluation of a Collaborative Quality Improvement Program to Improve Immunization Rate and Reduce Missed Opportunities for Vaccination in Primary Health-Care Facilities: A Time Series Study in Kano, Nigeria." *Expert Review of Vaccines* 18 (9): 969–91. https://doi.org/10/gg9pgt.

Admassie, Assefa, Degnet Abebaw, and Andinet D. Woldemichael. 2009. "Impact Evaluation of the Ethiopian Health Services Extension Programme." *Journal of Development Effectiveness* 1 (4): 430–49. https://doi.org/10/b7bgrs.

Aggarwal, Shilpa. 2018. "The Long Road to Health: Healthcare Utilization Impacts of a Road Pavement Policy in Rural India." Working paper. Hyderabad: Indian School of Business. http://eprints.exchange.isb.edu/884/.

Ahmed, Tashrik, Aneesa Arur, Damien de Walque, and Gil Shapira. 2019. "Incentivizing Quantity and Quality of Care: Evidence from an Impact Evaluation of Performance-Based Financing in the Health Sector in Tajikistan." Policy Research Working Papers 8951. Washington, DC: The World Bank. https://doi.org/10.1596/1813-9450-8951.

Alatas, Vivi, Arun Chandrasekhar, Markus Mobius, Benjamin Olken, and Cindy Paladines. 2019. "When Celebrities Speak: A Nationwide Twitter Experiment Promoting Vaccination in Indonesia." NBER Working Paper Series 25589. Cambridge, MA: National Bureau of Economic Research. https://doi.org/10.3386/w25589.

Alhassan, Robert Kaba, Edward Nketiah-Amponsah, Martin Amogre Ayanore, Agani Afaya, Solomon Mohammed Salia, Japiong Milipaak, Evelyn Korkor Ansah, and Seth Owusu-Agyei. 2019. "Impact of a Bottom-up Community Engagement Intervention on Maternal and Child Health Services Utilization in Ghana: A Cluster Randomised Trial." *BMC Public Health* 19 (1). https://doi.org/10/ghpbgx.

Amaral, João, Eleanor Gouws, Jennifer Bryce, Álvaro Jorge Madeiro Leite, Antonio Ledo Alves da Cunha, and Cesar G. Victora. 2004. "Effect of Integrated Management of Childhood Illness (IMCI) on Health Worker Performance in Northeast-Brazil." *Cadernos de Saúde Pública* 20 (suppl 2): S209–19. https://doi.org/10/dbvvrz.

Andersson, Neil, Anne Cockcroft, Noor M Ansari, Khalid Omer, Manzoor Baloch, Ari Ho Foster, Bev Shea, George A Wells, and José Legorreta Soberanis. 2009. "Evidence-Based Discussion Increases Childhood Vaccination Uptake: A Randomised Cluster Controlled Trial of Knowledge Translation in Pakistan." *BMC International Health and Human Rights* 9 (S1). https://doi.org/10/fpnrqq.

Andrade, Mônica Viegas, Flávia Chein, Laetícia Rodrigues de Souza, and Jaume Puig-Junoy. 2012. "Income Transfer Policies and the Impacts on the Immunization of Children: The Bolsa Família Program." *Cadernos de Saúde Pública* 28 (7): 1347–58. https://doi.org/10/gghf2x.

Andreoni, James, Michael Callen, Muhammad Yasir Khan, Karrar Jaffar, and Charles Sprenger. 2016. “Using Preference Estimates to Customize Incentives: An Application to Polio Vaccination Drives in Pakistan.” NBER Working Paper Series 22019. Cambridge, MA: National Bureau of Economic Research. https://doi.org/10.3386/w22019.

Anjum, Qudsia, Aamir Omair, S. N. Bazmi Inam, Yousuf Ahmed, Yaseen Usman, and Shazia Shaikh. 2004. “Improving Vaccination Status of Children under Five through Health Education.” *JPMA. The Journal of the Pakistan Medical Association* 54 (12): 610–13.

Aquino, Rosana, Nelson F. de Oliveira, and Mauricio L. Barreto. 2009. “Impact of the Family Health Program on Infant Mortality in Brazilian Municipalities.” *American Journal of Public Health* 99 (1): 87–93. https://doi.org/10/btntq8.

Arifeen, Shams E, DM Emdadul Hoque, Tasnima Akter, Muntasirur Rahman, Mohammad Enamul Hoque, Khadija Begum, Enayet K Chowdhury, et al. 2009. “Effect of the Integrated Management of Childhood Illness Strategy on Childhood Mortality and Nutrition in a Rural Area in Bangladesh: A Cluster Randomised Trial” 374: 393–403. https://doi.org/10/dc85jg.

Arraiz, Irani, and Sandra Rozo. 2011. “Same Bureaucracy, Different Outcomes in Human Capital? How Indigenous and Rural Non-Indigenous Areas in Panama Responded to the CCT.” OVE Working Papers 03/11. Washington, D.C.: Inter-American Development Bank. http://www.ssrn.com/abstract=1847124.

Assegaai, T, G Reagon, and H Schneider. 2018. “Evaluating the Effect of Ward-Based Outreach Teams on Primary Healthcare Performance in North West Province, South Africa: A Plausibility Design Using Routine Data.” *South African Medical Journal* 108 (4): 329–35. https://doi.org/10/gdn23v.

Atnafu, Asfaw, Kate Otto, and Christopher H. Herbst. 2017. “The Role of MHealth Intervention on Maternal and Child Health Service Delivery: Findings from a Randomized Controlled Field Trial in Rural Ethiopia.” *MHealth* 3 (September). https://doi.org/10/gcmhjc.

Attanasio, Orazio, Luis Carlos Gómez, Patricia Heredia, and Marcos Vera-Hernández. n.d. “The Short-Term Impact of a Conditional Cash Subsidy on Child Health and Nutrition in Colombia,” 15.

Ayieko, Philip, Stephen Ntoburi, John Wagai, Charles Opondo, Newton Opiyo, Santau Migiro, Annah Wamae, et al. 2011. “A Multifaceted Intervention to Implement Guidelines and Improve Admission Paediatric Care in Kenyan District Hospitals: A Cluster Randomised Trial.” Edited by Igor Rudan. *PLoS Medicine* 8 (4). https://doi.org/10/dtj73r.

Balasubramaniam, Sudharsanam, Somesh Kumar, Reena Sethi, Elaine Charurat, Kamlesh Lalchandani, Anne Schuster, and Bulbul Sood. 2018. “Quasi-Experimental Study of Systematic Screening for Family Planning Services among Postpartum Women Attending Village Health and Nutrition Days in Jharkhand, India.” *International Journal of Integrated Care* 18 (1). https://doi.org/10/gg9pgr.

Banerjee, A. V., E. Duflo, R. Glennerster, and D. Kothari. 2010. “Improving Immunisation Coverage in Rural India: Clustered Randomised Controlled Evaluation of Immunisation Campaigns with and without Incentives.” *BMJ* 340 (may17 1). https://doi.org/10/ffv4ff.

Banerjee, Abhijit, Arun Chandrasekhar, Esther Duflo, Suresh Dalpath, John Floretta, Matthew Jackson, Harini Kannan, Anna Schrimpf, and Maheshwor Shrestha. 2020. “Evaluating the Impact of Interventions to Improve Full Immunisation Rates in Haryana, India.” Impact

Evaluation Report 126. New Delhi: International Initiative for Impact Evaluation (3ie). https://doi.org/10.23846/TW10IE126.

Banerjee, Rakesh, and Ashish Sachdeva. 2015. "Pathways to Preventive Health, Evidence from India's Rural Road Program." USC-INET Research Paper 15–19. Los Angeles, CA: USC Dornsife Institute for New Economic Thinking. http://www.ssrn.com/abstract=2636999.

Bangure, Donewell, Daniel Chirundu, Notion Gombe, Tawanda Marufu, Gibson Mandozana, Mufuta Tshimanga, and Lucia Takundwa. 2015. "Effectiveness of Short Message Services Reminder on Childhood Immunization Programme in Kadoma, Zimbabwe - a Randomized Controlled Trial, 2013." *BMC Public Health* 15 (1). https://doi.org/10/f63zrg.

Banwat, M.E. 2015. "Effect of Peer Education on Knowledge, Attitude and Completeness of Childhood Routine Immunization in a Rural Community of Plateau State." *Research Journal of Health Sciences* 3 (4): 264–74. https://www.ajol.info/index.php/rejhs/article/view/143332.

Barham, Tania. 2005. "The Impact of the Mexican Conditional Cash Transfer Program on Immunization Rates." Boulder: University of Colorado. https://ibs.colorado.edu/barham/wp/CCTimmunWBfin.pdf.

Barham, Tania, Logan E. Brenzel, and John A. Maluccio. 2007. "Beyond 80%: Are There New Ways of Increasing Vaccination Coverage? Evaluation of CCT Programs in Mexico and Nicaragua." HNP Discussion Paper 41537. Washington: World Bank. http://www.ssrn.com/abstract=993760.

Barham, Tania, and John A. Maluccio. 2009. "Eradicating Diseases: The Effect of Conditional Cash Transfers on Vaccination Coverage in Rural Nicaragua." *Journal of Health Economics* 28 (3): 611–21. https://doi.org/10/czhvx3.

Basinga, Paulin, P. J. Gertler, Agnes Binagwaho, A. L. B. Soucat, J. R. Sturdy, and C. M. J. Vermeersch. 2009. "Impact of Performance Based Financing in Rwanda: Health Facility Level Analysis." Working Paper 32. New Delhi: Global Development Network. http://www.gdn.int/impact-performance-based-financing-rwanda-health-facility-level-analysis.

Basinga, Paulin, Paul J Gertler, Agnes Binagwaho, Agnes LB Soucat, Jennifer Sturdy, and Christel MJ Vermeersch. 2011. "Effect on Maternal and Child Health Services in Rwanda of Payment to Primary Health-Care Providers for Performance: An Impact Evaluation." *The Lancet* 377 (9775): 1421–28. https://doi.org/10/b2f469.

Beck, Simon, Anni-Maria Pulkki-Brännström, and Miguel San Sebastián. 2015. "Basic Income – Healthy Outcome? Effects on Health of an Indian Basic Income Pilot Project: A Cluster Randomised Trial." *Journal of Development Effectiveness* 7 (1): 111–26. https://doi.org/10/f3n3kb.

Berhane, Yemane, and Joyce Pickering. 1993. "Are Reminder Stickers Effective in Reducing Immunization Dropout Rates in Addis Ababa, Ethiopia?" *Journal of Tropical Medicine and Hygiene* 96 (3): 139–45. https://europepmc.org/article/med/8505766.

Bernal, Pedro, and Sebastian Martinez. 2020. "In-Kind Incentives and Health Worker Performance: Experimental Evidence from El Salvador." *Journal of Health Economics* 70 (March). https://doi.org/10/ghpbkj.

Biemba, Godfrey, Kojo Yeboah-Antwi, Kathryn Bradford Vosburg, Margaret L. Prust, Brett Keller, Yekoyesew Worku, Happy Zulu, Emily White, and Davidson H. Hamer. 2016. "Effect of

Deploying Community Health Assistants on Appropriate Treatment for Diarrhoea, Malaria and Pneumonia: Quasi-Experimental Study in Two Districts of Zambia.” *Tropical Medicine & International Health* 21 (8): 985–94. https://doi.org/10/gghf23.

Binyaruka, Peter, Edith Patouillard, Timothy Powell-Jackson, Giulia Greco, Ottar Maestad, and Josephine Borghi. 2015. “Effect of Paying for Performance on Utilisation, Quality, and User Costs of Health Services in Tanzania: A Controlled before and after Study.” Edited by Jan Ostermann. *PLOS ONE* 10 (8). https://doi.org/10/gghf22.

Björkman, Martina, and Jakob Svensson. 2009. “Power to the People: Evidence from a Randomized Field Experiment on Community-Based Monitoring in Uganda.” *Quarterly Journal of Economics* 124 (2): 735–69. https://doi.org/10/bzsr9k.

Bolam, A., D. S Manandhar, P. Shrestha, M. Ellis, and A. M d. L Costello. 1998. “The Effects of Postnatal Health Education for Mothers on Infant Care and Family Planning Practices in Nepal: A Randomised Controlled Trial.” *BMJ* 316 (7134): 805–11. https://doi.org/10/d3xmrk.

Bonfrer, Igna, Lyn Breebaart, and Ellen Van de Poel. 2016. “The Effects of Ghana’s National Health Insurance Scheme on Maternal and Infant Health Care Utilization.” Edited by Osman Alimamy Sankoh. *PLOS ONE* 11 (11). https://doi.org/10/f9q46z.

Bonfrer, Igna, Ellen Van de Poel, and Eddy Van Doorslaer. 2014. “The Effects of Performance Incentives on the Utilization and Quality of Maternal and Child Care in Burundi.” *Social Science & Medicine* 123 (December): 96–104. https://doi.org/10/f6vdkg.

Borkum, Evan, Anu Rangarajan, Dana Rotz, Swetha Sridharan, Sukhmani Sethi, and Mercy Manoranjini. 2014. “Evaluation of the Team-Based Goals and Performance Based Incentives (TBGI) Innovation in Bihar.” Mathematica Policy Research Reports d8e1097122ff47a6bf42580c8. Princeton: Mathematica Policy Research. https://ideas.repec.org/p/mpr/mprres/d8e1097122ff47a6bf42580c82677834.html.

Bossio, Juan Carlos, Ivan Sanchis, Gustavo Ariel Armando, Sergio Javier Arias, and Humberto Jure. 2019. “Resultado de una estrategia de recordatorios previos y posteriores a la fecha de vacunación para mejorar la oportunidad de la vacunación a los seis meses.” *Cadernos de Saúde Pública* 35 (12). https://doi.org/10/ghpbkt.

Bradley, Janet, and Susan Igras. 2005. “Improving the Quality of Child Health Services: Participatory Action by Providers.” *International Journal for Quality in Health Care* 17 (5): 391–99. https://doi.org/10/bdjp6m.

Brenner, Stephan, Rachel P. Chase, Shannon A. McMahon, Julia Lohmann, Christopher J. Makwero, Adamson S. Muula, and Manuela De Allegri. 2020. “Effect Heterogeneity in Responding to Performance-Based Incentives: A Quasi-Experimental Comparison of Impacts on Health Service Indicators between Hospitals and Health Centers in Malawi.” *Health Systems & Reform* 6 (1). https://doi.org/10/gg2njg.

Briere, E. C., T. K. Ryman, E. Cartwright, E. T. Russo, K. A. Wannemuehler, B. L. Nygren, S. Kola, et al. 2012. “Impact of Integration of Hygiene Kit Distribution with Routine Immunizations on Infant Vaccine Coverage and Water Treatment and Handwashing Practices of Kenyan Mothers.” *Journal of Infectious Diseases* 205 (suppl 1): S56–64. https://doi.org/10/f3wknp.

Brown, V. B., O. A. Oluwatosin, J. O. Akinyemi, and A. A. Adeyemo. 2016. “Effects of Community Health Nurse-Led Intervention on Childhood Routine Immunization Completion in Primary

Health Care Centers in Ibadan, Nigeria." *Journal of Community Health* 41 (2): 265–73. https://doi.org/10/f8czr8.

Brown, Victoria Bolanle, Oyeninhun Abimbola Oluwatosin, and Martins Olusola Ogundeji. 2017. "Impact of Training Intervention on Immunization Providers' Knowledge and Practice of Routine Immunization in Ibadan, South-Western Nigeria: A Primary Health Care Experience." *Pan African Medical Journal* 26. https://doi.org/10/ghpbkv.

Brughal, R F, and J P Kevany. 1996. "Maximizing Immunization Coverage through Home Visits: A Controlled Trial in an Urban Area of Ghana." *Bulletin of the World Health Organization* 74 (5): 517–24. https://www.ncbi.nlm.nih.gov/pmc/articles/PMC2486871/.

Busso, Matias, Julian Cristia, and Sarah Humpage. 2015. "Did You Get Your Shots? Experimental Evidence on the Role of Reminders." *Journal of Health Economics* 44 (December): 226–37. https://doi.org/10/f75n4m.

Cahyadi, Nur, Rema Hanna, Benjamin A. Olken, Rizal Adi Prima, Elan Satriawan, Ekki Syamsulhakim, and Rema Hanna. 2018. "Cumulative Impacts of Conditional Cash Transfer Programs: Experimental Evidence from Indonesia." NBER Working Paper Series 24670. Cambridge: National Bureau of Economic Research. https://www.nber.org/papers/w24670.

Carnell, Mary A, Leanne Dougherty, Amanda M Pomeroy, Ali M Karim, Yared M Mekonnen, and Brian E Mulligan. 2014. "Effectiveness of Scaling up the 'Three Pillars' Approach to Accelerating MDG 4 Progress in Ethiopia." *Journal of Health, Population and Nutrition* 32 (4): 549–63. https://pubmed.ncbi.nlm.nih.gov/25895187/.

Carrillo, Bladimir, Wilman J Iglesias, and Juan C Trujillo. 2015. "Attainments and Limitations of an Early Childhood Programme in Colombia." *Health Policy and Planning* 30 (7): 906–16. https://doi.org/10/f7vmx5.

Carvalho, Natalie, Naveen Thacker, Subodh S. Gupta, and Joshua A. Salomon. 2014. "More Evidence on the Impact of India's Conditional Cash Transfer Program, Janani Suraksha Yojana: Quasi-Experimental Evaluation of the Effects on Childhood Immunization and Other Reproductive and Child Health Outcomes." Edited by Jeremy D. Goldhaber-Fiebert. *PLoS ONE* 9 (10). https://doi.org/10/gghf24.

Chakrabarti, Averi, Karen A. Grépin, and Stéphane Helleringer. 2019. "The Impact of Supplementary Immunization Activities on Routine Vaccination Coverage: An Instrumental Variable Analysis in Five Low-Income Countries." Edited by Vijayaprasad Gopichandran. *PLOS ONE* 14 (2). https://doi.org/10/gghf25.

Chandir, S., A.J. Khan, H. Hussain, H.R. Usman, S. Khowaja, N.A. Halsey, and S.B. Omer. 2010. "Effect of Food Coupon Incentives on Timely Completion of DTP Immunization Series in Children from a Low-Income Area in Karachi, Pakistan: A Longitudinal Intervention Study." *Vaccine* 28 (19): 3473–78. https://doi.org/10/cz4mk3.

Chansa, Collins, Ashis Das, Jumana Qamruddin, Jed Friedman, Akafwilangachi Mkandawire, and Monique Vledder. 2015. "Linking Results to Performance: Evidence from a Results Based Financing Pre-Pilot Project in Katete District, Zambia." Discussion Paper 98265. Washington, DC: The World Bank. https://openknowledge.worldbank.org/handle/10986/22390?show=full.

Chelagat, T., G. Kokwaro, J. Onyango, and J. Rice. 2020. "Effect of Project-Based Experiential Learning on the Health Service Delivery Indicators: A Quasi-Experiment Study." *BMC Health Services Research* 20 (1). https://doi.org/10/gg9pgv.

Chen, Li, Xiaozhen Du, Lin Zhang, Michelle Helena van Velthoven, Qiong Wu, Ruikan Yang, Ying Cao, et al. 2016. "Effectiveness of a Smartphone App on Improving Immunization of Children in Rural Sichuan Province, China: A Cluster Randomized Controlled Trial." *BMC Public Health* 16 (1). https://doi.org/10/f82vfx.

Chen, Yvonne Jie, Namrata Chindarkar, and Yun Xiao. 2019. "Effect of Reliable Electricity on Health Facilities, Health Information, and Child and Maternal Health Services Utilization: Evidence from Rural Gujarat, India." *Journal of Health, Population and Nutrition* 38 (1). https://doi.org/10/ghpbk6.

Costa-Font, Joan, and Divya Parmar. 2017. "Political Agency and Public Health Care: Evidence from India." CESifo Working Papers 6640. Munich: UNU-WIDER. https://doi.org/10.35188/UNU-WIDER/2016/179-6.

Cristia, Julian, William N. Evans, and Beomsoo Kim. 2015. "Improving the Health Coverage of the Rural Poor: Does Contracting-out Mobile Medical Teams Work?" *The Journal of Development Studies* 51 (3). https://doi.org/10/ghpwp8.

Cristia, Julián P, William N Evans, and Beomsoo Kim. 2011. "Does Contracting-out Primary Care Services Work? The Case of Rural Guatemala." IDB Working Paper Series IDB-WP-273. Washington. https://www.econstor.eu/handle/10419/88975.

Cristia, Julian, Ariadna García Prado, and Cecilia Peluffo. 2015. "The Impact of Contracting in and Contracting out Basic Health Services: The Guatemalan Experience." *World Development* 70 (June): 215–27. https://doi.org/10/gghf27.

Cruzado de la Vega, Viviana. 2017. "Pagos por desempeño para mejorar estado nutricional infantil: impacto de los convenios de apoyo presupuestario en tres regiones peruanas con alta prevalencia de desnutrición cronica infantil, 2010-2014." *Revista Peruana de Medicina Experimental y Salud Pública* 34 (3): 365–76. https://doi.org/10/gghf28.

Daoud, Adel, and Bernhard Reinsberg. 2019. "Structural Adjustment, State Capacity and Child Health: Evidence from IMF Programmes." *International Journal of Epidemiology* 48 (2): 445–54. https://doi.org/10/gfqr5z.

Demilew, Abebual, Mesay Girma, Elizabeth McElwee, Saugato Datta, Jeremy Barofsky, and Tolera Disasa. 2021. "Improving Immunisation through Supportive Feedback and Nonmonetary Incentives: Impact Evaluation in Ethiopia." Impact Evaluation Report. New Delhi: International Initiative for Impact Evaluation (3ie).

Dicko, Alassane, Sidy O Toure, Mariam Traore, Issaka Sagara, Ousmane B Toure, Mahamadou S Sissoko, Alpha T Diallo, et al. 2011. "Increase in EPI Vaccines Coverage after Implementation of Intermittent Preventive Treatment of Malaria in Infant with Sulfadoxine -Pyrimethamine in the District of Kolokani, Mali: Results from a Cluster Randomized Control Trial." *BMC Public Health* 11 (1). https://doi.org/10/cvr2cj.

Dipeolu, Isaac O. 2017. "Effect of Mobile-Phone Reminder Test Messages on Mothers' Knowledge and Completion of Routine Immunisation in Rural Areas of Oyo State, Nigeria." PhD Thesis, Ibadan, Nigeria: University of Ibadan. https://library.adhl.africa/handle/123456789/11865.

Dissieka, Romance, Marissa Soohoo, Amynah Janmohamed, and David Doledec. 2019. "Providing Mothers with Mobile Phone Message Reminders Increases Childhood Immunisation and Vitamin A Supplementation Coverage in Côte d'Ivoire: A Randomised Controlled Trial." *Journal of Public Health in Africa* 10 (1): 56–60. https://doi.org/10/ghpbk9.

Djibuti, Mamuka, George Gotsadze, Akaki Zoidze, George Mataradze, Laura C Esmail, and Jillian Clare Kohler. 2009. “The Role of Supportive Supervision on Immunization Program Outcome - a Randomized Field Trial from Georgia.” *BMC International Health and Human Rights* 9 (S1). https://doi.org/10/dxd2rk.

Domek, Gretchen J., Ingrid L. Contreras-Roldan, Sheana Bull, Sean T. O’Leary, Guillermo Antonio Bolaños Ventura, Michael Bronsert, Allison Kempe, and Edwin J. Asturias. 2019. “Text Message Reminders to Improve Infant Immunization in Guatemala: A Randomized Clinical Trial.” *Vaccine* 37 (42): 6192–6200. https://doi.org/10/ghpbmb.

Domek, Gretchen J., Ingrid L. Contreras-Roldan, Sean T. O’Leary, Sheana Bull, Anna Furniss, Allison Kempe, and Edwin J. Asturias. 2016. “SMS Text Message Reminders to Improve Infant Vaccination Coverage in Guatemala: A Pilot Randomized Controlled Trial.” *Vaccine* 34 (21): 2437–43. https://doi.org/10/f8nccx.

Drain, Paul K, Josoa S Ralaivao, Alexander Rakotonandrasana, and Mary A Carnell. 2003. “Introducing Auto-Disable Syringes to the National Immunization Programme in Madagascar.” *Bulletin of the World Health Organization*, 8.

D’Souza, Prathibha V, and J Umarani. 2014. “Teaching Package Improves Mothers Knowledge on Vaccine Preventable Diseases and Vaccination: A Quasi Experimental Study.” *International Journal of Research in Medical Sciences* 2 (3): 976–82. https://doi.org/10/ghpv3h.

Dykstra, Sarah, Amanda Glassman, Charles Kenny, and Justin Sandefur. 2019. “Regression Discontinuity Analysis of Gavi’s Impact on Vaccination Rates.” *Journal of Development Economics* 140 (September): 12–25. https://doi.org/10/gg9pgw.

Edoka, Ijeoma, Tim Ensor, Barbara McPake, Rogers Amara, Fu-Min Tseng, and Joseph Edem-Hotah. 2016. “Free Health Care for Under-Fives, Expectant and Recent Mothers? Evaluating the Impact of Sierra Leone’s Free Health Care Initiative.” *Health Economics Review* 6 (1). https://doi.org/10/gghf29.

Eichler, Rena, Paul Auxila, Uder Antoine, and Bernateau Desmangles. 2007. “Performance-Based Incentives for Health: Six Years of Results from Supply-Side Programs in Haiti.” CGD Working Paper 121. Washington, DC: Center for Global Development. http://www.ssrn.com/abstract=1003249.

Ekhaguere, Osayame A, Rosena O Oluwafemi, Bolaji Badejoko, Lawal O Oyeneyin, Azeez Butali, Elizabeth D Lowenthal, and Andrew P Steenhoff. 2019. “Automated Phone Call and Text Reminders for Childhood Immunisations (PRIMM): A Randomised Controlled Trial in Nigeria.” *BMJ Global Health* 4 (2). https://doi.org/10/gghf3b.

Engineer, Cyrus Y, Elina Dale, Anubhav Agarwal, Arunika Agarwal, Olakunle Alonge, Anbrasi Edward, Shivam Gupta, Holly B Schuh, Gilbert Burnham, and David H Peters. 2016. “Effectiveness of a Pay-for-Performance Intervention to Improve Maternal and Child Health Services in Afghanistan: A Cluster-Randomized Trial.” *International Journal of Epidemiology* 45 (2): 451–59. https://doi.org/10/gghf3c.

Eze, G U, and A O Adeleye. 2015. “Enhancing Routine Immunization Performance Using Innovative Technology in an Urban Area of Nigeria.” *West African Journal of Medicine* 34 (1): 3–10. https://pubmed.ncbi.nlm.nih.gov/26902809/.

Findley, Sally E, Omolara T Uwemedimo, Henry V Doctor, Cathy Green, Fatima Adamu, and Godwin Y Afenyadu. 2013. “Early Results of an Integrated Maternal, Newborn, and Child

Health Program, Northern Nigeria, 2009 to 2011." *BMC Public Health* 13 (1). https://doi.org/10/gbfgsv.

Gajate-Garrido, Gissele, and Clement Ahiadeke. 2012. "The Effect of Parents' Insurance Enrollment on Health Care Utilization: Evidence from Ghana." SSRN Scholarly Paper ID 2158824. Rochester, NY: Social Science Research Network. https://doi.org/10.2139/ssrn.2158824.

Garly, M. L., C. L. Martins, C. Bale, F. da Costa, F. Dias, H. Whittle, and P. Aaby. 1999. "Early Two-Dose Measles Vaccination Schedule in Guinea-Bissau: Good Protection and Coverage in Infancy." *International Journal of Epidemiology* 28 (2): 347–52. https://doi.org/10/cgqfgz.

Gauri, Varun. 2009. "Do International Treaties Promote Development? The Convention on the Rights of the Child and Basic Immunization." Policy Research Working Paper 4964. Washington: The World Bank. https://doi.org/10.1596/1813-9450-4964.

Gauri, Varun, and Peyvand Khaleghian. 2002. "Immunization in Developing Countries: Its Political and Organizational Determinants." Policy Research Working Paper 2769. Washington: The World Bank. https://www.infona.pl/resource/bwmeta1.element.elsevier-4a3b393c-c1da-3dff-9b7c-91c05767fb9d.

Gibson, Dustin G, Benard Ochieng, E Wangeci Kagucia, Joyce Were, Kyla Hayford, Lawrence H Moulton, Orin S Levine, Frank Odhiambo, Katherine L O'Brien, and Daniel R Feikin. 2017. "Mobile Phone-Delivered Reminders and Incentives to Improve Childhood Immunisation Coverage and Timeliness in Kenya (M-SIMU): A Cluster Randomised Controlled Trial." *The Lancet Global Health* 5 (4): e428–38. https://doi.org/10/f9tm87.

Gilbert, Sarah Skye, Ngwegwe Bulula, Emmanuel Yohana, Jenny Thompson, Emily Beylerian, Laurie Werner, and Jessica C. Shearer. 2020. "The Impact of an Integrated Electronic Immunization Registry and Logistics Management Information System (EIR-ELMIS) on Vaccine Availability in Three Regions in Tanzania: A Pre-Post and Time-Series Analysis." *Vaccine* 38 (3): 562–69. https://doi.org/10/ghpbsr.

Goel, Sonu, Vishal Dogra, Satish Kumar Gupta, P. V. M. Lakshmi, Sherin Varkey, Narottam Pradhan, Gopal Krishna, and Rajesh Kumar. 2012. "Effectiveness of Muskaan Ek Abhiyan (The Smile Campaign) for Strengthening Routine Immunization in Bihar, India." *Indian Pediatrics* 49 (2): 103–8. https://doi.org/10/gghf3d.

Goodson, James L., Manisha A. Kulkarni, Jodi L. Vanden Eng, Kathleen A. Wannemuehler, Annett H. Cotte, Rachelle E. Desrochers, Bakolalao Randriamanalina, and Elizabeth T. Luman. 2012. "Improved Equity in Measles Vaccination from Integrating Insecticide-Treated Bednets in a Vaccination Campaign, Madagascar: Equity in Measles Vaccination in Madagascar." *Tropical Medicine & International Health* 17 (4): 430–37. https://doi.org/10/fxsrbw.

Guindon, G. Emmanuel. 2014. "The Impact of Health Insurance on Health Services Utilization and Health Outcomes in Vietnam." *Health Economics, Policy and Law* 9 (4): 359–82. https://doi.org/10/f6g47g.

Gultiano, Soccoro A., and Elizabeth M. King. 2006. "A Better Start in Life: Evaluation Results from an Early Childhood Development Program." *Philippine Journal of Development* 33 (1/2): 101–28. https://ideas.repec.org/p/phd/pjdevt/pjd_2006_vol__xxxiii_nos__1and2-d.html.

Gurley, Nikki, Jessica Shearer, Yachna Srivastava, Sudip Mahapatra, and Michelle Desmond. 2020. "Impacts of Community-Led Video Education to Increase Vaccination Coverage in Uttar

Pradesh, India." Impact Evaluation Report 125. 2020th ed. New Delhi: International Initiative for Impact Evaluation (3ie). https://doi.org/10.23846/TW10IE125.

Habib, Muhammad Atif, Sajid Soofi, Simon Cousens, Saeed Anwar, Najib ul Haque, Imran Ahmed, Noshad Ali, Rehman Tahir, and Zulfiqar A Bhutta. 2017. "Community Engagement and Integrated Health and Polio Immunisation Campaigns in Conflict-Affected Areas of Pakistan: A Cluster Randomised Controlled Trial." *The Lancet Global Health* 5 (6): e593–603. https://doi.org/10/f962m2.

Hagiwara, Akiko, Mika Ueyama, Asad Ramlawi, and Yasuyuki Sawada. 2013. "Is the Maternal and Child Health (MCH) Handbook Effective in Improving Health-Related Behavior? Evidence from Palestine." *Journal of Public Health Policy* 34 (1): 31–45. https://doi.org/10/ghpbss.

Haji, Adam Hassan. 2017. "Evaluation of SMS and Sticker Reminders in Reducing Dropout Rates in Routine Child Immunization in Selected Districts in Kenya." Master Thesis, Juja: Jomo Kenyatta University of Agriculture and Technology. http://ir.jkuat.ac.ke/handle/123456789/3069.

Hajizadeh, Mohammad, Jody Heymann, Erin Strumpf, Sam Harper, and Arijit Nandi. 2015. "Paid Maternity Leave and Childhood Vaccination Uptake: Longitudinal Evidence from 20 Low-and-Middle-Income Countries." *Social Science & Medicine* 140 (September): 104–17. https://doi.org/10/f7ppdx.

Hategeka, Celestin, Hinda Ruton, and Michael R. Law. 2019. "Effect of a Community Health Worker MHealth Monitoring System on Uptake of Maternal and Newborn Health Services in Rwanda." *Global Health Research and Policy* 4 (1). https://doi.org/10/ghpbst.

Heinrich, Carolyn J., and Matthew T. Knowles. 2020. "A Fine Predicament: Conditioning, Compliance and Consequences in a Labeled Cash Transfer Program." *World Development* 129 (May). https://doi.org/10/ghpbsv.

Helleringer, Stephane, Patrick O. Asuming, and Jalaa Abdelwahab. 2016. "The Effect of Mass Vaccination Campaigns against Polio on the Utilization of Routine Immunization Services: A Regression Discontinuity Design." *Vaccine* 34 (33): 3817–22. https://doi.org/10/f8w56m.

Herrera-Almanza, Catalina, and Maria F. Rosales-Rueda. 2020. "Reducing the Cost of Remoteness: Community-Based Health Interventions and Fertility Choices." *Journal of Health Economics* 73 (September): 102365. https://doi.org/10/ghrbtg.

Hu, Yu, Yaping Chen, Ying Wang, Quanwei Song, and Qian Li. 2017. "Prenatal Vaccination Education Intervention Improves Both the Mothers' Knowledge and Children's Vaccination Coverage: Evidence from Randomized Controlled Trial from Eastern China." *Human Vaccines & Immunotherapeutics* 13 (6): 1477–84. https://doi.org/10/gghf3f.

Huillery, Elise, and Juliette Seban. 2019. "Financial Incentives, Efforts, and Performances in the Health Sector: Experimental Evidence from the Democratic Republic of Congo." *Economic Development and Cultural Change*, March. https://doi.org/10/ghpbsq.

Hutchinson, Paul, Peter Lance, David K. Guilkey, Mohammad Shahjahan, and Shahida Haque. 2006. "Measuring the Cost-Effectiveness of a National Health Communication Program in Rural Bangladesh." *Journal of Health Communication* 11 (sup2): 91–121. https://doi.org/10/b7wdkt.

Ikilezi, Gloria, Orvalho J. Augusto, Joseph L. Dieleman, Kenneth Sherr, and Stephen S. Lim. 2020. "Effect of Donor Funding for Immunization from GAVI and Other Development Assistance Channels on Vaccine Coverage: Evidence from 120 Low and Middle Income Recipient Countries." *Vaccine* 38 (3): 588–96. https://doi.org/10/ghpwdv.

Ikilezi, Gloria, Orvalho J Augusto, Alyssa Sbarra, Kenneth Sherr, Joseph L Dieleman, and Stephen S Lim. 2020. "Determinants of Geographical Inequalities for DTP3 Vaccine Coverage in Sub-Saharan Africa." *Vaccine* 38 (18): 3447–54. https://doi.org/10/ghpwdw.

Janssens, Wendy. 2011. "Externalities in Program Evaluation: The Impact of a Women's Empowerment Program on Immunization." *Journal of the European Economic Association* 9 (6): 1082–1113. https://doi.org/10/fbr8s8.

Jaupart, Pascal, Lizzie Dipple, and Stefan Dercon. 2019. "Has Gavi Lived up to Its Promise? Quasi-Experimental Evidence on Country Immunisation Rates and Child Mortality." *BMJ Global Health* 4 (6). https://doi.org/10/ghpwdx.

Johri, Mira, Dinesh Chandra, Karna Georges Kone, Marie-Pierre Sylvestre, Alok K Mathur, Sam Harper, and Arijit Nandi. 2020. "Social and Behavior Change Communication Interventions Delivered Face-to-Face and by a Mobile Phone to Strengthen Vaccination Uptake and Improve Child Health in Rural India: Randomized Pilot Study." *JMIR MHealth and UHealth* 8 (9). https://doi.org/10/ghpwdz.

Kagucia, E Wangeci. 2018. "MHealth Interventions to Improve Measles Vaccination Coverage and Timeliness: An Assessment of the Immediate and Long-Term Impact on Vaccine-Seeking in Rural Kenya." PhD Thesis, Baltimore, MD: Johns Hopkins University. https://jscholarship.library.jhu.edu/handle/1774.2/61130.

Kamatsuchi, Mahoko, Adrian Gheorghe, and Dina Balabanova. 2019. "The Global Scale and Implications of Delivering Multiple Interventions through Integrated Child Health Events." *BMJ Global Health* 4 (4). https://doi.org/10/ghpwd2.

Kandpal, Eeshani, Harold Alderman, Jed Friedman, Deon Filmer, Junko Onishi, and Jorge Avalos. 2016. "A Conditional Cash Transfer Program in the Philippines Reduces Severe Stunting." *The Journal of Nutrition* 146 (9): 1793–1800. https://doi.org/10/f8zrr6.

Karing, Anne. 2018. "Social Signaling and Childhood Immunization: A Field Experiment in Sierra Leone." Berkeley Working Paper. Berkeley: University of California. https://economics.yale.edu/sites/default/files/jmp_socialsignaling.pdf.

Kawakatsu, Yoshito, Tomohiko Sugishita, Kennedy Oruenjo, Stephen Wakhule, Kennedy Kibosia, Eric Were, and Sumihisa Honda. 2015. "Effectiveness of and Factors Related to Possession of a Mother and Child Health Handbook: An Analysis Using Propensity Score Matching." *Health Education Research* 30 (October): 935–46. https://doi.org/10/f749wb.

Kazi, Abdul Momin, Murtaza Ali, Khurram Zubair, Hussain Kalimuddin, Abdul Nafey Kazi, Saleem Perwaiz Iqbal, Jean-Paul Collet, and Syed Asad Ali. 2018. "Effect of Mobile Phone Text Message Reminders on Routine Immunization Uptake in Pakistan: Randomized Controlled Trial." *JMIR Public Health and Surveillance* 4 (1). https://doi.org/10/ghpwd3.

Kern, Ana Paula, Marcel de Toledo Vieira, and Ricardo da Silva Freguglia. 2018. "Impactos Do Programa Bolsa Família Na Imunização Das Crianças." Working paper. https://www.anpec.org.br/encontro/2018/submissao/files_I/i12-cb6dfc0f070c8ca9e77abc8563c3801f.pdf.

Khaleghian, Peyvand. 2003. “Decentralization and Public Services: The Case of Immunization.” Policy Research Working Paper 2989. Washington: The World Bank. https://openknowledge.worldbank.org/handle/10986/19159?show=full.

Kusuma, Dian, Hasbullah Thabrany, Budi Hidayat, Margaret McConnell, Peter Berman, and Jessica Cohen. 2017. “New Evidence on the Impact of Large-Scale Conditional Cash Transfers on Child Vaccination Rates: The Case of a Clustered-Randomized Trial in Indonesia.” *World Development* 98 (October): 497–505. https://doi.org/10/ghpwd4.

Lamanna, Camillo, and Lauren Byrne. 2019. “A Pilot Study of a Novel, Incentivised MHealth Technology to Monitor the Vaccine Supply Chain in Rural Zambia.” *Pan African Medical Journal* 33. https://doi.org/10/ghpwp5.

Lee, Scott S. 2015. “Three Field Experiments on Incentives for Health Workers.” PhD Thesis, Cambridge: Harvard University. https://dash.harvard.edu/handle/1/17467500.

Levy, Dan, and Jim Ohls. 2010. “Evaluation of Jamaica’s PATH Conditional Cash Transfer Programme.” *Journal of Development Effectiveness* 2 (4): 421–41. https://doi.org/10/dtkp26.

Lin, Meng Ann. 2016. “Stimulating Demand: An Assessment of the Conditional Cash Transfer Project in Afghanistan.” PhD Thesis, Baltimore: Johns Hopkins University. https://jscholarship.library.jhu.edu/handle/1774.2/39740.

Liu, Yong, Zhaokang Yuan, Yuxi Liu, Upali W Jayasinghe, and Mark F Harris. 2014. “Changing Community Health Service Delivery in Economically Less-Developed Rural Areas in China: Impact on Service Use and Satisfaction.” *BMJ Open* 4 (2). https://doi.org/10/f6mwb9.

Loevinsohn, B P, and E Gareaballah. 1992. “Missed Opportunities for Immunization during Visits for Curative Care: A Randomized Cross-over Trial in Sudan.” *Bulletin of the World Health Organization* 70 (3): 335–39. https://www.ncbi.nlm.nih.gov/pmc/articles/PMC2393287/.

Loevinsohn, Benjamin P., and Michael E. Loevinsohn. 1986. “Improvement in Coverage of Primary Health Care in a Developing Country through Use of Food Incentives.” *The Lancet* 327 (8493): 1314–16. https://doi.org/10/bjgw7d.

Looij, Frank van de, Dudzai Mureyi, Chenjerai Sisimayi, Jaap Koot, Portia Manangazira, and Nyasha Musuka. 2015. “Early Evidence from Results-Based Financing in Rural Zimbabwe.” *African Health Monitor* 6: 32–36. https://collaboration.worldbank.org/content/sites/collaboration-for-development/en/groups/results-based-financing/groups/oba-rbf-newsroom/documents.entry.html/2016/01/14/early_evidence_from-Kgih.html.

Maluccio, John A., and Rafael Flores. 2005. “Impact Evaluation of a Conditional Cash Transfer Program the Nicaraguan Red de Protección Social.” Research report 141. 0 ed. Washington, DC: International Food Policy Research Institute. https://doi.org/10.2499/0896291464RR141.

Mampe, Tumelo, Helen Schneider, and Gavin Reagon. 2016. “Effectiveness of Ward Based Outreach Teams in the North West Province: An Evaluation.” Cape Town: University of the Western Cape. http://rgdoi.net/10.13140/RG.2.2.36191.66725.

Masuda, Kazuya, and Chikako Yamauchi. 2020. “How Does Female Education Reduce Adolescent Pregnancy and Improve Child Health? Evidence from Uganda’s Universal Primary Education for Fully Treated Cohorts.” *The Journal of Development Studies* 56 (1): 63–86. https://doi.org/10/ggv6vq.

Mathanga, Don P., Elizabeth T. Luman, Carl H. Campbell, Chimwemwe Silwimba, and Grace Malenga. 2009. “Integration of Insecticide-Treated Net Distribution into Routine Immunization Services in Malawi: A Pilot Study.” *Tropical Medicine & International Health* 14 (7): 792–801. https://doi.org/10/b44rkh.

Mayumana, Iddy, Jo Borghi, Laura Anselmi, Masuma Mamdani, and Siri Lange. 2017. “Effects of Payment for Performance on Accountability Mechanisms: Evidence from Pwani, Tanzania.” *Social Science & Medicine* 179 (April): 61–73. https://doi.org/10/f95w4p.

Mazumder, S., S. Taneja, R. Bahl, P. Mohan, T. A. Strand, H. Sommerfelt, B. R. Kirkwood, et al. 2014. “Effect of Implementation of Integrated Management of Neonatal and Childhood Illness Programme on Treatment Seeking Practices for Morbidities in Infants: Cluster Randomised Trial.” *BMJ* 349 (August). https://doi.org/10/gb3snq.

McMahon, Shannon A., Stephan Brenner, Julia Lohmann, Christopher Makwero, Aleksandra Torbica, Don P. Mathanga, Adamson S. Muula, and Manuela De Allegri. 2016. “Evaluating Complex Health Financing Interventions: Using Mixed Methods to Inform Further Implementation of a Novel PBI Intervention in Rural Malawi.” *BMC Health Services Research* 16 (1). https://doi.org/10/f8zjkr.

Mehta, Kala M., Francois Rerolle, Sonali V. Rammohan, Davis C. Albohm, George Muwowo, Heidi Moseson, Lesley Sept, Hau L. Lee, and Eran Bendavid. 2016. “Systematic Motorcycle Management and Health Care Delivery: A Field Trial.” *American Journal of Public Health* 106 (1): 87–94. https://doi.org/10/f8hd4m.

Memon, Zahid A, Gul N Khan, Sajid B Soofi, Imam Y Baig, and Zulfiqar A Bhutta. 2015. “Impact of a Community-Based Perinatal and Newborn Preventive Care Package on Perinatal and Neonatal Mortality in a Remote Mountainous District in Northern Pakistan.” *BMC Pregnancy and Childbirth* 15 (1). https://doi.org/10/f7df7k.

Mensah, Joseph, Joseph R Oppong, and Christoph M Schmidt. 2009. “Ghana’s National Health Insurance Scheme in the Context of the Health MDGs: An Empirical Evaluation Using Propensity Score Matching.” Ruhr Economic Papers 157. https://papers.ssrn.com/sol3/papers.cfm?abstract_id=1532169.

Modi, Dhiren, Nishith Dholakia, Ravi Gopalan, Sethuraman Venkatraman, Kapilkumar Dave, Shobha Shah, Gayatri Desai, et al. 2019. “MHealth Intervention ‘ImTeCHO’ to Improve Delivery of Maternal, Neonatal, and Child Care Services—A Cluster-Randomized Trial in Tribal Areas of Gujarat, India.” Edited by Jenny E Myers. *PLOS Medicine* 16 (10). https://doi.org/10/gg66n6.

Mohan, Pavitrra, Baya Kishore, Sharad Singh, Rajiv Bahl, Anju Puri, and Rajesh Kumar. 2012. “Assessment of Implementation of Integrated Management of Neonatal and Childhood Illness in India.” *Journal of Health, Population and Nutrition* 29 (6): 629–38. https://doi.org/10/ghpwft.

Mohanan, Manoj, Vikram S. Rajan, Kendal Swanson, and Harsha Thirumurthy. 2020. “Information and Facilitation Interventions for Accountability in Health and Nutrition: Evidence from a Randomized Trial in India.” ERID Working Paper 295. Durham: Duke University. https://www.ssrn.com/abstract=3544786.

More, Neena Shah, Ujwala Bapat, Sushmita Das, Glyn Alcock, Sarita Patil, Maya Porel, Leena Vaidya, Armida Fernandez, Wasundhara Joshi, and David Osrin. 2012. “Community Mobilization in Mumbai Slums to Improve Perinatal Care and Outcomes: A Cluster

Randomized Controlled Trial." Edited by A. Metin Gülmezoglu. *PLoS Medicine* 9 (7). https://doi.org/10/p5w.

More, Neena Shah, Sushmita Das, Ujwala Bapat, Glyn Alcock, Shreya Manjrekar, Vikas Kamble, Rijuta Sawant, et al. 2017. "Community Resource Centres to Improve the Health of Women and Children in Informal Settlements in Mumbai: A Cluster-Randomised, Controlled Trial." *The Lancet Global Health* 5 (3): e335–49. https://doi.org/10/f9tpkj.

Morris, Saul S, Rafael Flores, Pedro Olinto, and Juan Manuel Medina. 2004. "Monetary Incentives in Primary Health Care and Effects on Use and Coverage of Preventive Health Care Interventions in Rural Honduras: Cluster Randomised Trial." *The Lancet* 364 (9450): 2030–37. https://doi.org/10/ckxp28.

Murthy, Nirmala, Subhashini Chandrasekharan, Muthu Perumal Prakash, Nadi N. Kaonga, Joanne Peter, Aakash Ganju, and Patricia N. Mechael. 2019. "The Impact of an MHealth Voice Message Service (MMitra) on Infant Care Knowledge, and Practices among Low-Income Women in India: Findings from a Pseudo-Randomized Controlled Trial." *Maternal and Child Health Journal* 23 (12): 1658–69. https://doi.org/10/ghpwfx.

Musa, O I, D B Parakoyi, and A A Akanbi. 2006. "Evaluation of Health Education Intervention on Safe Immunization Injection among Health Workers in Ilorin, Nigeria." *Annals of African Medicine* 5 (3): 122–28. https://www.semanticscholar.org/paper/Evaluation-of-Health-Education-Intervention-on-Safe-Musa-Parakoyi/5f3eaa0090f82149c7de44c442ec094d31a04591?p2df.

Nagar, Ruchit, Mohammad Sarparajul Ambiya, Khushi Baby, Logan Stone, Deepa Manjanatha, and Preethi Venkat. 2020. "A Mixed-Methods Evaluation to Determine the Effects of a Novel MHealth Platform for Maternal Child Health Tracking in Rural Udaipur, India." 3ie Series Report 129. New Delhi: International Initiative for Impact Evaluation (3ie). https://developmentevidence.3ieimpact.org/search-result-details/impact-evaluation-repository/a-mixed-methods-evaluation-to-determine-the-effects-of-a-novel-mhealth-platform-for-maternal-child-health-tracking-in-rural-udaipur-india/8984.

Nagar, Ruchit, Preethi Venkat, Logan D. Stone, Kyle A. Engel, Praneeth Sadda, and Mohammed Shahnawaz. 2018. "A Cluster Randomized Trial to Determine the Effectiveness of a Novel, Digital Pendant and Voice Reminder Platform on Increasing Infant Immunization Adherence in Rural Udaipur, India." *Vaccine* 36 (44): 6567–77. https://doi.org/10/gfqfwq.

Nanyunja, Miriam, Rosamund F. Lewis, Issa Makumbi, Rachel Seruyange, Eva Kabwongera, Possy Mugyenyi, and Ambrose Talisuna. 2003. "Impact of Mass Measles Campaigns among Children Less than 5 Years Old in Uganda." *The Journal of Infectious Diseases* 187 (s1): S63–68. https://doi.org/10/bzksr6.

Nasir, Narila Mutia, Yuli Amran, and Yasuhide Nakamura. 2017. "Changing Knowledge and Practices of Mothers on Newborn Care through Mother Class: An Intervention Study in Indonesia." *Journal of Tropical Pediatrics* 63 (6): 440–46. https://doi.org/10/gghf3q.

Nzioki, Japheth Mativo, James Ouma, James Hebert Ombaka, and Rosebella Ongutu Onyango. 2017. "Community Health Worker Interventions Are Key to Optimal Infant Immunization Coverage, Evidence from a Pretest-Posttest Experiment in Mwingi, Kenya." *Pan African Medical Journal* 28. https://doi.org/10/gghf3p.

Oche, M. O., A. S. Umar, M. T. O. Ibrahim, and K. Sabitu. 2011. “An Assessment of the Impact of Health Education on Maternal Knowledge and Practice of Childhood Immunization in Kware, Sokoto State.” *Journal of Public Health and Epidemiology* 3 (10): 440–47. https://academicjournals.org/article/article1379497483_Oche%20et%20al.pdf.

Okeke, Edward, Peter Glick, Isa Sadeeq Abubakar, AV Chari, Emma Pitchforth, Josephine Exley, Usman Bashir, Claude Setodji, Kun Gu, and Obinna Onwujekwe. 2017. “Better Obstetrics in Rural Nigeria: Evaluating the Midwives Service Scheme.” 3ie Impact Evaluation Report 56. International Initiative for Impact Evaluation (3ie). https://www.3ieimpact.org/evidence-hub/publications/impact-evaluations/better-obstetrics-rural-nigeria-evaluating-midwives.

Okoli, Ugo, Laura Morris, Adetokunbo Oshin, Muhammad A Pate, Chidimma Aigbe, and Ado Muhammad. 2014. “Conditional Cash Transfer Schemes in Nigeria: Potential Gains for Maternal and Child Health Service Uptake in a National Pilot Programme.” *BMC Pregnancy and Childbirth* 14 (1). https://doi.org/10/f6tssk.

Olayo, Rose, Charles Wafula, Evalyne Aseyo, Constantine Loum, and Dan Kaseje. 2014. “A Quasi-Experimental Assessment of the Effectiveness of the Community Health Strategy on Health Outcomes in Kenya.” *BMC Health Services Research* 14 (S1). https://doi.org/10/gb36rc.

Olken, Benjamin A., Junko Onishi, and Susan Wong. 2014. “Should Aid Reward Performance? Evidence from a Field Experiment on Health and Education in Indonesia.” *American Economic Journal: Applied Economics* 6 (4): 1–34. https://doi.org/10/gf7j3c.

Onishi, Junko. 2014. “Philippines Conditional Cash Transfer Program Impact Evaluation 2012.” World Bank Report 75533-PH. Washington, DC: The World Bank. https://assessments.hpc.tools/sites/default/files/assessments/Philippines%20Conditional%20Cash%20Transfer%20Program%2C%20Impact%20Evaluation%202012.pdf.

Owais, Aatekah, Beenish Hanif, Amna R Siddiqui, Ajmal Agha, and Anita KM Zaidi. 2011. “Does Improving Maternal Knowledge of Vaccines Impact Infant Immunization Rates? A Community-Based Randomized-Controlled Trial in Karachi, Pakistan.” *BMC Public Health* 11 (1). https://doi.org/10/bpswbk.

Oyo-Ita, Angela, Xavier Bosch-Capblanch, Amanda Ross, Patrick Hanlon, Afiong Oku, Ekperonne Esu, Soter Ameh, Bisi Oduwole, Dachi Arikpo, and Martin Meremikwu. 2020. “Impacts of Engaging Communities through Traditional and Religious Leaders on Vaccination Coverage in Cross River State, Nigeria.” Impact Evaluation Report 127. 2020th ed. New Delhi: International Initiative for Impact Evaluation (3ie). https://doi.org/10.23846/TW10IE127.

Özer, Mustafa, Jan Fidrmuc, and Mehmet Ali Eryurt. 2018. “Maternal Education and Childhood Immunization in Turkey.” *Health Economics* 27 (8): 1218–29. https://doi.org/10/gdkgd3.

Padilla, Alcides de J., and Juan C. Trujillo. 2015. “An Impact Assessment of the Child Growth, Development and Care Program in the Caribbean Region of Colombia.” *Cadernos de Saúde Pública* 31 (10): 2099–2109. https://doi.org/10/gghf3w.

Pandey, Priyanka, Ashwini R. Sehgal, Michelle Riboud, David Levine, and Madhav Goyal. 2007. “Informing Resource-Poor Populations and the Delivery of Entitled Health and Social Services in Rural India: A Cluster Randomized Controlled Trial.” *JAMA* 298 (16): 1867–75. https://doi.org/10/ddxdhz.

Pathak, Yuvraj, and Karen Macours. 2017. "Women's Political Reservation, Early Childhood Development, and Learning in India." *Economic Development and Cultural Change* 65 (4): 741–66. https://doi.org/10/ghpwf6.

Powell-Jackson, Timothy, Camilla Fabbri, Varun Dutt, Sarah Tougher, and Kultar Singh. 2018. "Effect and Cost-Effectiveness of Educating Mothers about Childhood DPT Vaccination on Immunisation Uptake, Knowledge, and Perceptions in Uttar Pradesh, India: A Randomised Controlled Trial." Edited by James K. Tumwine. *PLOS Medicine* 15 (3). https://doi.org/10/gc4pqg.

Pramanik, Santanu, Arpita Ghosh, Rituu B. Nanda, Marlou de Rouw, Philip Forth, and Sandra Albert. 2018. "Impact Evaluation of a Community Engagement Intervention in Improving Childhood Immunization Coverage: A Cluster Randomized Controlled Trial in Assam, India." *BMC Public Health* 18 (1). https://doi.org/10/gdhbjf.

Prinja, Shankar, Ruby Nimesh, Aditi Gupta, Pankaj Bahuguna, Madhu Gupta, and Jarnail Singh Thakur. 2017. "Impact of M-Health Application Used by Community Health Volunteers on Improving Utilisation of Maternal, New-Born and Child Health Care Services in a Rural Area of Uttar Pradesh, India." *Tropical Medicine & International Health* 22 (7): 895–907. https://doi.org/10/gghf35.

Prosser, Wendy, Philippe Jaillard, Emmanuelle Assy, Shawn T. Brown, Graça Matsinhe, Mawutondji Dekoun, and Bruce Y. Lee. 2017. "System Redesign of the Immunization Supply Chain: Experiences from Benin and Mozambique." *Vaccine* 35 (17): 2162–66. https://doi.org/10/f96jsd.

Rahman, Atif, Abid Malik, Siham Sikander, Christopher Roberts, and Francis Creed. 2008. "Cognitive Behaviour Therapy-Based Intervention by Community Health Workers for Mothers with Depression and Their Infants in Rural Pakistan: A Cluster-Randomised Controlled Trial." *The Lancet* 372 (9642): 902–9. https://doi.org/10/fsjx85.

Rahman, Mahfuzar, Fakir Md. Yunus, Rasheduzzaman Shah, Fatema Tuz Jhohura, Sabuj Kanti Mistry, Tasmeen Quayyum, Bachera Aktar, and Kaosar Afsana. 2016. "A Controlled Before-and-after Perspective on the Improving Maternal, Neonatal, and Child Survival Program in Rural Bangladesh: An Impact Analysis." Edited by Gabriel Gebhardt. *PLOS ONE* 11 (9). https://doi.org/10/f9q958.

Rahman, Mohammad Mahbubur, and Saseendran Pallikadavath. 2019. "Maternal and Child Health Care Services' Utilization Data from the Fourth Round of District Level Household Survey in India." *Data in Brief* 23 (April). https://doi.org/10/ghpwgm.

Rajkotia, Yogesh, Omer Zang, Pierre Nguimkeu, Jessica Gergen, Iva Djurovic, Paula Vaz, Franscisco Mbofana, and Kebba Jobarteh. 2017. "The Effect of a Performance-Based Financing Program on HIV and Maternal/Child Health Services in Mozambique—an Impact Evaluation." *Health Policy and Planning* 32 (10): 1386–96. https://doi.org/10/gcfv5b.

Rao, Tanvi. 2014. "The Impact of a Community Health Worker Program on Childhood Immunization: Evidence from India's 'ASHA' Workers." http://www.ssrn.com/abstract=2444391.

Rasella, Davide, Rosana Aquino, Carlos AT Santos, Rômulo Paes-Sousa, and Mauricio L Barreto. 2013. "Effect of a Conditional Cash Transfer Programme on Childhood Mortality: A Nationwide Analysis of Brazilian Municipalities." *The Lancet* 382 (9886): 57–64. https://doi.org/10/f2kfx4.

Reinbold, Gary W. 2019. "Effects of the Convention on the Rights of the Child on Child Mortality and Vaccination Rates: A Synthetic Control Analysis." *BMC International Health and Human Rights* 19 (1). https://doi.org/10/ghgdqs.

Robertson, Laura, Phyllis Mushati, Jeffrey W Eaton, Lovemore Dumba, Gideon Mavise, Jeremiah Makoni, Christina Schumacher, et al. 2013. "Effects of Unconditional and Conditional Cash Transfers on Child Health and Development in Zimbabwe: A Cluster-Randomised Trial." *The Lancet* 381 (9874): 1283–92. https://doi.org/10/f2hmx3.

Robinson, J Stephen, Barton R Burkhalter, Barbie Rasmussen, and Ristianto Sugiono. 2001. "Low-Cost on-the-Job Peer Training of Nurses Improved Immunization Coverage in Indonesia." *Bulletin of the World Health Organization* 79 (2): 150–58. https://www.scielosp.org/article/bwho/2001.v79n2/150-158/en/.

Roth, A. E., C. Stabell Benn, H. Ravn, A. Rodrigues, I. M. Lisse, M. Yazdanbakhsh, H. Whittle, and P. Aaby. 2010. "Effect of Revaccination with BCG in Early Childhood on Mortality: Randomised Trial in Guinea-Bissau." *BMJ* 340 (mar15 1). https://doi.org/10/c8q74b.

Roy, Swapan Kumar, Farzana Bilkes, Khaleda Islam, Gulshan Ara, Phillip Tanner, Irena Wosk, Ahmed Shafiqur Rahman, Barnali Chakraborty, Saira Parveen Jolly, and Wahjiah Khatun. 2008. "Impact of Pilot Project of Rural Maintenance Programme (RMP) on Destitute Women: CARE, Bangladesh." *Food and Nutrition Bulletin* 29 (1): 67–75. https://doi.org/10/gghf3z.

Rusa, Louis, Jean de Dieu Ngirabega, Willy Janssen, Stefaan Van Bastelaere, Denis Porignon, and Werner Vandenbulcke. 2009. "Performance-Based Financing for Better Quality of Services in Rwandan Health Centres: 3-Year Experience." *Tropical Medicine & International Health* 14 (7): 830–37. https://doi.org/10/cc5zvh.

Ryman, Tove K., Elizabeth C. Briere, Emily Cartwright, Karen Schlanger, Kathleen A. Wannemuehler, Elizabeth T. Russo, Steve Kola, et al. 2012. "Integration of Routine Vaccination and Hygiene Interventions: A Comparison of 2 Strategies in Kenya." *The Journal of Infectious Diseases* 205 (suppl_1): S65–76. https://doi.org/10/f3wkpb.

Ryman, Tove K., Ajay Trakroo, Aaron Wallace, Satish Kumar Gupta, Karen Wilkins, Pankaj Mehta, and Vance Dietz. 2011. "Implementation and Evaluation of the Reaching Every District (RED) Strategy in Assam, India, 2005–2008." *Vaccine* 29 (14): 2555–60. https://doi.org/10/bjqf85.

Saggurti, Niranjan, Yamini Atmavilas, Akash Porwal, Janine Schooley, Rajshree Das, Narender Kande, Laili Irani, and Katherine Hay. 2018. "Effect of Health Intervention Integration within Women's Self-Help Groups on Collectivization and Healthy Practices around Reproductive, Maternal, Neonatal and Child Health in Rural India." Edited by Alessandra N. Bazzano. *PLOS ONE* 13 (8). https://doi.org/10/gd4w3d.

Salami, Lamidhi, Edgard-Marius Dona Ouendo, and Benjamin Fayomi. 2016. "Effects of Results Based Financing Models on Data Quality Improvement in Benin on 2014." *Universal Journal of Public Health* 4 (6): 324–31. https://doi.org/10/ghpwgc.

Salami, Lamidhi, Edgard-Marius Ouendo, and Benjamin Fayomi. 2018. "Effects of Results Based Financing Models on the Performance of Exposed Health Zones in Benin." *International Journal Of Community Medicine And Public Health* 5 (10): 4188–99. https://doi.org/10/ghpwgd.

Sangwan, Santosh, and Anju Manocha. 2009. "Maternal Knowledge and Child Health." *Journal of Human Ecology* 25 (1): 51–54. https://doi.org/10/gghf33.

Sankar, Deepa. 2013. “Improving Early Childhood Development through Community Mobilization and Integrated Planning for Children. Results from the Evaluation of Bachpan Program, Ratlam District, Madhya Pradesh, India.” Discussion Paper Series 59. Washington, D.C.: The World Bank. http://crossasia-repository.ub.uni-heidelberg.de/3467/.

Sato, Ryoko, and Abdullahi Belel. 2020. “The Effect of Performance-Based Financing on Child Vaccinations in Northern Nigeria.” *Vaccine* 38 (9): 2209–15. https://doi.org/10/ghpwgf.

Schlumberger, M., A. Bamoko, T.M. Yaméogo, F. Rouvet, R. Ouedraogo, B. Traoré, M. Tinto, et al. 2015. “Impact positif sur le Programme élargi de vaccinations de l’envoi de SMS de rappel à partir d’un registre informatisé, Bobo-Dioulasso (Burkina Faso).” *Bulletin de la Société de pathologie exotique* 108 (5): 349–54. https://doi.org/10/gghf34.

Sengupta, Nandana, and Aakanksha Sinha. 2018. “Is India’s Safe Motherhood Scheme Leading to Better Child Health Care Practices?” *Global Social Welfare* 5 (1): 49–58. https://doi.org/10/ghpwgg.

Seth, Rajeev, Ibukunoluwa Akinboyo, Ankur Chhabra, Yawar Qaiyum, Anita Shet, Nikhil Gupte, Ajay K. Jain, and Sanjay K. Jain. 2018. “Mobile Phone Incentives for Childhood Immunizations in Rural India.” *Pediatrics* 141 (4): e20173455. https://doi.org/10/gdcwg5.

Shei, Amie, Federico Costa, Mitermayer G Reis, and Albert I Ko. 2014. “The Impact of Brazil’s Bolsa Família Conditional Cash Transfer Program on Children’s Health Care Utilization and Health Outcomes.” *BMC International Health and Human Rights* 14 (1). https://doi.org/10/gbf36j.

Sherry, Tisamarie B., Sebastian Bauhoff, and Manoj Mohanan. 2017. “Multitasking and Heterogeneous Treatment Effects in Pay-for-Performance in Health Care: Evidence from Rwanda.” *American Journal of Health Economics* 3 (2): 192–226. https://doi.org/10/ghpwgh.

Shuaib, Waqas, Julia Marielly Suarez, Juan David Romero, Carlos Dillon Pamello, Richard Alweis, Aizaaz Ali Khan, Syed Raza Shah, Hassan Shahid, Serge B. PierreCharles, and Laura Rosemary Sanchez. 2016. “Transforming Patient Care by Introducing an Electronic Medical Records Initiative in a Developing Country.” *Health Informatics Journal* 22 (4): 975–83. https://doi.org/10/gghf36.

Shukla, Mahesh. 2018. “Impact of a Health Governance Intervention on Provincial Health System Performance in Afghanistan: A Quasi-Experimental Study.” *Health Systems & Reform* 4 (3): 249–66. https://doi.org/10/ghpwgj.

Siddiqi, Danya Arif, Rozina Feroz Ali, Mehr Munir, Mubarak Taighoon Shah, Aamir Javed Khan, and Subhash Chandir. 2020. “Effect of Vaccine Reminder and Tracker Bracelets on Routine Childhood Immunization Coverage and Timeliness in Urban Pakistan (2017-18): A Randomized Controlled Trial.” *BMC Public Health* 20 (1): 1086. https://doi.org/10/ghq97m.

Sinha, Nistha, and Joanne K Yoong. 2009. “Long-Term Financial Incentives and Investment in Daughters: Evidence from Conditional Cash Transfers in North India.” RAND Labor and Population working paper series WR-667. Santa Monica: RAND Corporation. https://doi.org/10.1596/1813-9450-4860.

Soeters, Robert, Peter Bob Peerenboom, Pacifique Mushagalusa, and Célestin Kimanuka. 2011. “Performance-Based Financing Experiment Improved Health Care in the Democratic Republic of Congo.” *Health Affairs* 30 (8): 1518–27. https://doi.org/10/c6w2rq.

Steinhardt, L. C., I. Aman, I. Pakzad, B. Kumar, L. P. Singh, and D. H. Peters. 2011. “Removing User Fees for Basic Health Services: A Pilot Study and National Roll-out in Afghanistan.” *Health Policy and Planning* 26 (Suppl. 2): ii92–103. https://doi.org/10/drchbp.

Subedi, Sudarshan, Bishnu Prasad Sharma, and Chiranjivi Adhikari. 2018. “Cost and Consequences of Integrated Public Health Campaign in Baglung District, Nepal.” *Economic Journal of Development Issues* 23 & 24 (January): 113–22. https://doi.org/10/ghpwgk.

Talukder, Md., Ubaidur Rob, Syed Abu Musa, Ashish Bajracharya, Kaji Keya, Forhana Noor, Eshita Jahan, Md. Hossain, Jyotirmoy Saha, and Benjamin Bellows. 2014. “Evaluation of the Impact of the Voucher Program for Improving Maternal Health Behavior and Status in Bangladesh.” Dhaka: Population Council. https://doi.org/10.31899/rh10.1000.

Tandon, B. N., and A. Sahai. 1988. “Immunization in India: Contribution of Integrated Child Development Services Scheme to Expanded Programme of Immunization.” *Journal of Tropical Pediatrics* 34 (6): 309–12. https://doi.org/10/ghpwgr.

Tanner, Jeffery, Ryotaro Hayashi, and Yunsun Li. 2015. “Improving Coverage and Utilization of Maternal and Child Health Services in Lao PDR: Impact Evaluation of the Community Nutrition Project.” IEG Working Paper 99929. Washington: The World Bank Group. https://documents.worldbank.org/en/publication/documents-reports/documentdetail/441841468187767237/improving-coverage-and-utilization-of-maternal-and-child-health-services-in-lao-pdr-impact-evaluation-of-the-community-nutrition-project.

Thomas, Ranjeeta Alice. 2011. “Essays on Ex Ante Evaluations of Cash Transfer Programs.” PhD Thesis, York: University of York. http://etheses.whiterose.ac.uk/2004/.

Thomson, Dana R, Cheryl Amoroso, Sidney Atwood, Matthew H Bonds, Felix Cyamatare Rwabukwisi, Peter Drobac, Karen E Finnegan, et al. 2018. “Impact of a Health System Strengthening Intervention on Maternal and Child Health Outputs and Outcomes in Rural Rwanda 2005–2010.” *BMJ Global Health* 3 (2). https://doi.org/10/gdcrw5.

Thysen, Sanne Marie, Stine Byberg, Marie Pedersen, Amabelia Rodrigues, Henrik Ravn, Cesario Martins, Christine Stabell Benn, Peter Aaby, and Ane Bærent Fisker. 2014. “BCG Coverage and Barriers to BCG Vaccination in Guinea-Bissau: An Observational Study.” *BMC Public Health* 14 (1). https://doi.org/10/f6k4xv.

Uddin, Md. Jasim, Md. Shamsuzzaman, Lily Horng, Alain Labrique, Lavanya Vasudevan, Kelsey Zeller, Mridul Chowdhury, Charles P. Larson, David Bishai, and Nurul Alam. 2016. “Use of Mobile Phones for Improving Vaccination Coverage among Children Living in Rural Hard-to-Reach Areas and Urban Streets of Bangladesh.” *Vaccine* 34 (2): 276–83. https://doi.org/10/f766t2.

Unger, Jean-Pierre. 1991. “Can Intensive Campaigns Dynamize Front Line Health Services? The Evaluation of an Immunization Campaign in Thiès Health District, Senegal.” *Social Science & Medicine* 32 (3): 249–59. https://doi.org/10/bc5fn2.

USAID. 2008. “Essential Services for Health in Ethiopia: Final Report.” Washington, DC: USAID. https://publications.jsi.com/JSIInternet/Inc/Common/_download_pub.cfm?id=10111&lid=3.

Usman, Hussain R., Saeed Akhtar, Faiza Habib, and Imtiaz Jehan. 2009. “Redesigned Immunization Card and Center-Based Education to Reduce Childhood Immunization Dropouts in Urban Pakistan: A Randomized Controlled Trial.” *Vaccine* 27 (3): 467–72. https://doi.org/10/bcv8fd.

Usman, Hussain R., Mohammad H. Rahbar, Sibylle Kristensen, Sten H. Vermund, Russell S. Kirby, Faiza Habib, and Eric Chamot. 2011. “Randomized Controlled Trial to Improve Childhood Immunization Adherence in Rural Pakistan: Redesigned Immunization Card and Maternal Education.” *Tropical Medicine & International Health* 16 (3): 334–42. https://doi.org/10/dm9tm7.

Vaidyanathan, Radha. 2019. “Immunization Coverage among Under-Five Children Living along a School Student through Child-to-Child and Child-to-Parent Information, Education and Communication Strategy.” *Indian Journal of Public Health* 63 (4): 334–40. https://doi.org/10/ghpwgq.

Van de Poel, Ellen, Gabriela Flores, Por Ir, and Owen O’Donnell. 2016. “Impact of Performance-Based Financing in a Low-Resource Setting: A Decade of Experience in Cambodia.” *Health Economics* 25 (6): 688–705. https://doi.org/10/f8kzhk.

Varghese, Beena, Reetabrata Roy, Somen Saha, and Sidsel Roalkvam. 2014. “Fostering Maternal and Newborn Care in India the Yashoda Way: Does This Improve Maternal and Newborn Care Practices during Institutional Delivery?” Edited by Hamid Reza Baradaran. *PLoS ONE* 9 (1). https://doi.org/10/f5s7zr.

Verma, M, J Chhatwal, and P V Varughese. 1995. “Antenatal Period: An Educational Opportunity.” *Indian Pediatrics* 32: 171–77. https://www.semanticscholar.org/paper/Antenatal-period%3A-an-educational-opportunity.-Verma-Chhatwal/b44c9c679879ada229d8a2719b5637155190f4d1?p2df.

Wadhwa, Sagar. 2019. “Conditional Cash Transfers and Parental Investment in Daughters: Evidence from India.” Paper presented at the Population Association of America 2019 Annual Meeting, Austin, TX. http://paa2019.populationassociation.org/abstracts/193315.

Wagstaff, Adam, and Shengchao Yu. 2007. “Do Health Sector Reforms Have Their Intended Impacts ? The World Bank’S Health VIII Project in Gansu Province, China.” *Journal of Health Economics* 26: 505–35. https://doi.org/10/cx4kc6.

Wallace, Aaron S., Kenny Peetosutan, Andi Untung, Marisa Ricardo, Prima Yosephine, Kathleen Wannemuehler, David W. Brown, et al. 2019. “Home-Based Records and Vaccination Appointment Stickers as Parental Reminders to Reduce Vaccination Dropout in Indonesia: A Cluster-Randomized Controlled Trial.” *Vaccine* 37 (45): 6814–23. https://doi.org/10/ghpwgw.

Walque, Damien de, Paul Jacob Robyn, Hamadou Saidou, Gaston Sorgho, and Maria Steenland. 2017. “Looking into the Performance-Based Financing Black Box: Evidence from an Impact Evaluation in the Health Sector in Cameroon.” Policy Research Working Paper 8162. Washington, DC: The World Bank. https://doi.org/10.1596/1813-9450-8162.

Wang, Paul C., Albert Mwango, Sarah Moberley, Benjamin J. Brockman, Alison L. Connor, Penelope Kalesha-Masumbu, Simon Mutembo, et al. 2015. “A Cluster Randomised Trial on the Impact of Integrating Early Infant HIV Diagnosis with the Expanded Programme on Immunization on Immunization and HIV Testing Rates in Rural Health Facilities in Southern Zambia.” Edited by David Joseph Diemert. *PLOS ONE* 10 (10). https://doi.org/10/f779tz.

Webster, Jayne, Justine Landegger, Jane Bruce, Dickson Malunda, Tracey Chantler, Edward Kumakech, Laura Schmucker, Lilian Kiapi, Naoko Kozuki, and Comfort Olorunsaiye. 2019. “Impacts of IRC’s Fifth Child Community Engagement Strategy to Increase Immunisation in Northern Uganda.” 3ie Grantee Final Report. New Delhi: International Initiative for Impact

Evaluation (3ie). https://www.3ieimpact.org/sites/default/files/2019-02/gfr-TW10.1018-IRC-Imunization-Program-Uganda.pdf.

Weldemariam, Mesele. 2010. "The Impact of Fiscal Decentralization on Education and Health Outcomes in Ethiopia: A Regional Panel Data Analysis." Master Thesis, Addis Ababa: Addis Ababa University. http://etd.aau.edu.et/handle/123456789/14217?show=full.

Wong, Benjamin KC, Shaza A Fadel, Shally Awasthi, Ajay Khera, Rajesh Kumar, Geetha Menon, and Prabhat Jha. 2019. "The Impact of Measles Immunization Campaigns in India Using a Nationally Representative Sample of 27,000 Child Deaths." *ELife* 8 (March). https://doi.org/10/ghpwgx.

Yoong, Joanne. 2007. "Does Decentralization Hurt Childhood Immunization?" Stanford: Stanford University. http://citeseerx.ist.psu.edu/viewdoc/summary?doi=10.1.1.370.4021.

Yoosuf, Abdul-Sattar. 1993. "Group Learning by Mothers about Primary Health Care." *World Health Forum* 14 (1): 20–22. https://pubmed.ncbi.nlm.nih.gov/8439363/.

Younes, Leila, Tanja A J Houweling, Kishwar Azad, Abdul Kuddus, Sanjit Shaha, Bedowra Haq, Tasmin Nahar, et al. 2015. "The Effect of Participatory Women's Groups on Infant Feeding and Child Health Knowledge, Behaviour and Outcomes in Rural Bangladesh: A Controlled before-and-after Study." *Journal of Epidemiology and Community Health* 69 (4): 374–81. https://doi.org/10/f65x9m.

Zang, Omer, Sebastien Djienouassi, Gaston Sorgho, and Jean Claude Taptue. 2015. "Impact of Performance-Based Financing on Health-Care Quality and Utilization in Urban Areas of Cameroon." *African Health Monitor* 7 (21): 10–14. https://indexmedicus.afro.who.int/aim/opac_css/doc_num.php?explnum_id=72209.

Zeng, Wu, Donald S Shepard, Jean de Dieu Rusatira, Aaron P Blaakman, and Bernice M Nsitou. 2018. "Evaluation of Results-Based Financing in the Republic of the Congo: A Comparison Group Pre–Post Study." *Health Policy and Planning* 33 (3): 392–400. https://doi.org/10/gcvmpw.

Zeng, Wu, Daxin Sun, Henry Mphwanthe, Tianwen Huan, Jae Eun Nam, Pascal Saint-Firmin, Gerald Manthalu, Suneeta Sharma, and Arin Dutta. 2019. "The Impact and Cost-Effectiveness of User Fee Exemption by Contracting out Essential Health Package Services in Malawi." *BMJ Global Health* 4 (2). https://doi.org/10/gghf4b.

Zizien, Zawora Rita, Catherine Korachais, Philippe Compaoré, Valéry Ridde, and Vincent De Brouwere. 2019. "Contribution of the Results-based Financing Strategy to Improving Maternal and Child Health Indicators in Burkina Faso." *The International Journal of Health Planning and Management* 34 (1): 111–29. https://doi.org/10/gd2zrp.

Zombré, David, Manuela De Allegri, and Valéry Ridde. 2020. "No Effects of Pilot Performance-Based Intervention Implementation and Withdrawal on the Coverage of Maternal and Child Health Services in the Koulikoro Region, Mali: An Interrupted Time Series Analysis." *Health Policy and Planning* 35 (4): 379–87. https://doi.org/10/ggnfpf.

Systematic reviews

High confidence

Kaufman J, Ryan R, Walsh L, Horey D, Leask J, Robinson P, Hill S, 2018. Face-to-face interventions for informing or educating parents about early childhood vaccination. Cochrane Database of Systematic Reviews 5, CD010038.

Lagarde, 2009a. The impact of conditional cash transfers on health outcomes and use of health services in low and middle income countries.

Lagarde, 2009b. The impact of contracting out on health outcomes and use of health services in low and middle-income countries.

Linde, 2019. One-way SMS and healthcare outcomes in Africa: Systematic review of randomised trials with meta-analysis.

Molina, Ezequiel Laura Carella, Ana Pacheco, Guillermo Cruces, Leonardo Gasparini, 2016. Community monitoring interventions to curb corruption and increase access and quality of service delivery in low- and middle-income countries: a systematic review. Campbell Systematic Reviews 12.

Odendaal Willem A, Ward Kim, Uneke Jesse, Uro-Chukwu Henry, Chitama Dereck, Balakrishna Yusentha, Kredo Tamara, 2018. Contracting out to improve the use of clinical health services and health outcomes in low- and middle-income countries. Cochrane Database of Systematic Reviews 4, CD008133. https://doi.org/10.1002/14651858.CD008133.pub2

Oyo-Ita Angela, Wiysonge Charles S, Oringanje Chioma, Nwachukwu Chukwuemeka E, Oduwole Olabisi, Meremikwu Martin M, 2016. Interventions for improving coverage of childhood immunisation in low- and middle-income countries. The Cochrane database of systematic reviews 7, CD008145–CD008145.

Witter, 2012. Paying for performance to improve the delivery of health interventions in low- and middle-income countries.

Medium confidence

Atkinson, 2019. Effectiveness of digital technologies at improving vaccine uptake and series completion–A systematic review and meta-analysis of randomized controlled trials.

Bassani Diego G, Paul Arora, Wazny Kerri, Gaffey Michelle F, Lenters Lindsey, Zulfiqar A Bhutta, 2013. Financial incentives and coverage of child health interventions: a systematic review and meta-analysis. BMC Public Health 13, 1–13. https://doi.org/10.1186/1471-2458-13-S3-S30

Bhutta, 2008. Interventions to address maternal, newborn, and child survival: what difference can integrated primary health care strategies make?

Gilmore, 2013. Effectiveness of community health workers delivering preventive interventions for maternal and child health in low- and middle-income countries: a systematic review.

Johri M, Pérez M C, Arsenault C, Sharma J K, Pai N P, Pahwa S, Sylvestre M P, 2015. Strategies to increase the demand for childhood vaccination in low- and middle-income countries: A

systematic review and meta-analysis. Bulletin of the World Health Organization 93, 339-346C. https://doi.org/10.2471/BLT.14.146951

Lukusa L A, Ndze V N, Mbeye N M, Wiysonge C S, 2018. A systematic review and meta-analysis of the effects of educating parents on the benefits and schedules of childhood vaccinations in low and middle-income countries. Human vaccines & immunotherapeutics 14, 1–31. https://doi.org/10.1080/21645515.2018.1457931

Mekonnen, 2019. Effect of mobile text message reminders on routine childhood vaccination: a systematic review and meta-analysis.

Saeterdal I, Lewin S, Austvoll-Dahlgren A, Glenton C, Munabi-Babigumira S, 2014. Interventions aimed at communities to inform and/or educate about early childhood vaccination. Cochrane Database of Systematic Reviews CD010232.

Yuan B, Malqvist M, Trygg N, Qian X, Ng N, Thomsen S, 2014. What interventions are effective on reducing inequalities in maternal and child health in low- and middle-income settings? A systematic review. BMC Public Health 14, 634.

Low confidence

Aaby, 2016. Randomized Trials Comparing Inactivated Vaccine After Medium-or High-titer Measles Vaccine With Standard Titer Measles Vaccine After Inactivated Vaccine.

Abdulrahman, 2017. mHealth: a narrative synthesis of evidence of its application in improving childhood immunization coverage.

Baequni, 2012. Is Maternal and Child Health Handbook Effective? Meta-Analysis of the Effects of MCH Handbook.

Batt, 2004. The costs, effects and cost-effectiveness of strategies to increase coverage of routine immunizations in low- and middle-income countries: systematic review of the grey literature.

Bright Tess, Felix Lambert, Kuper Hannah, Polack Sarah, 2017. A systematic review of strategies to increase access to health services among children in low and middle income countries. BMC Health Services Research 17, 1–19. https://doi.org/10.1186/s12913-017-2180-9

Chutiyami M, Wyver S, Amin J, 2019. Are parent-held child health records a valuable health intervention? A systematic review and meta-analysis. International Journal of Environmental Research and Public Health 16. https://doi.org/10.3390/ijerph16020220

Crocker-Buque Tim, Mindra Godwin, Duncan Richard, Mounier-Jack Sandra, 2017. Immunization, urbanization and slums - a systematic review of factors and interventions. BMC Public Health 17, 1–16. https://doi.org/10.1186/s12889-017-4473-7

Das, 2016. Effect of pay for performance to improve quality of maternal and child care in low-and middle-income countries: a systematic review.

de Souza Cruz, Rebeca Carmo, Azevedo de Moura, Leides Barroso, Soares Neto, Joaquim José, 2017. Conditional cash transfers and the creation of equal opportunities of health for children in low and middle-income countries: a literature review. International Journal for Equity in Health 16, 1–12. https://doi.org/10.1186/s12939-017-0647-2

Deardorff K V, Rubin Means, A, Ásbjörnsdóttir K H, Walson J, 2018. Strategies to improve treatment coverage in community-based public health programs: A systematic review of the literature. PLoS Neglected Tropical Diseases 12. https://doi.org/10.1371/journal.pntd.0006211

Freeman Paul A, Schleiff Meike, Sacks Emma, Rassekh Bahie M, Gupta Sundeep, Perry Henry B, 2017. Comprehensive review of the evidence regarding the effectiveness of community-based primary health care in improving maternal, neonatal and child health: 4. child health findings. Journal of Global Health 7, 367–387. https://doi.org/10.7189/jogh.07.010904

Gera Tarun, Shah Dheeraj, Garner Paul, Richardson Marty, Sachdev Harshpal S, 2016. Integrated management of childhood illness (IMCI) strategy for children under five. Cochrane Database of Systematic Reviews CD010123. https://doi.org/10.1002/14651858.CD010123.pub2

Giedion, 2013. The impact of universal coverage schemes in the developing world: a review of the existing evidence.

Harvey H, Reissland N, Mason J, 2015. Parental reminder, recall and educational interventions to improve early childhood immunisation uptake: A systematic review and meta-analysis. Vaccine 33, 2862–2880. https://doi.org/10.1016/j.vaccine.2015.04.085

Hatt Laurel, Ben Johns, Catherine Connor, Megan Meline, Matt Kukla, Kaelan Moat, 2015. Impact of Health Systems Strengthening on Health.

Iwu C J, Jaca A, Abdullahi L H, Ngcobo N J, Wiysonge C S, 2019. A scoping review of interventions for vaccine stock management in primary health-care facilities. Human Vaccines and Immunotherapeutics 15, 2666–2672. https://doi.org/10.1080/21645515.2019.1607130

Jarrett C, Wilson R, O’Leary M, Eckersberger E, Larson H J, 2015. Strategies for addressing vaccine hesitancy - a systematic review. (Special Issue: WHO recommendations regarding vaccine hesitancy.). Vaccine 33, 4180–4190.

Karageorgos, 2018. The promise of mobile technologies for the health care system in the developing world: a systematic review.

Kim S S, Patel M, Hinman A, 2017. Use of m-Health in polio eradication and other immunization activities in developing countries. Vaccine 35, 1373–1379.

Loevinsohn B, April Harding, 2004. Contracting for the delivery of community health services: A review of Global Experience Report No. 31506.

Munk, 2019. Systematic review of the costs and effectiveness of interventions to increase infant vaccination coverage in low-and middle-income countries.

Mureed S, Somronghtong R, Kumar R, Ghaffar A, Chapman R S, 2015. Enhanced immunization coverage through interventions for childhood cluster diseases in developing countries. Journal of Ayub Medical College, Abbottabad : JAMC 27, 223–227.

Naugle Danielle A, Hornik Robert C, 2014. Systematic review of the effectiveness of mass media interventions for child survival in low- and middle-income countries. Journal of Health Communication 19, 190–215.

Nelson Kristin N, Wallace Aaron S, Sodha Samir V, Daniels Danni, Dietz Vance, 2016. Assessing strategies for increasing urban routine immunization coverage of childhood vaccines in low and middle-income countries: A systematic review of peer-reviewed literature. Vaccine 34, 5495–5503. https://doi.org/10.1016/j.vaccine.2016.09.038

Nutman S, McKee D, Khoshnood K, 2013. Externalities of prevention of mother-to-child transmission programs: A systematic review. AIDS and Behavior 17, 445–460. https://doi.org/10.1007/s10461-012-0228-8

Oliver-Williams C, Brown E, Devereux S, Fairhead C, Holeman I, 2017. Using Mobile Phones to Improve Vaccination Uptake in 21 Low- and Middle-Income Countries: Systematic Review. JMIR MHealth and UHealth 5, e148.

Omoniyi, 2019. Realist Synthesis of the International Theory and Evidence on Strategies to Improve Childhood Vaccination in Low- and Middle-Income Countries: Developing Strategies for the Nigerian Healthcare System.

Owusu-Addo Ebenezer, Cross Ruth, 2014. The impact of conditional cash transfers on child health in low- and middle-income countries: A systematic review. International Journal of Public Health 59, 609–618.

Ozawa, 2018. Systematic review of the incremental costs of interventions that increase immunization coverage.

Pal Moneeta, Goodyear-Smith Felicity, Exeter Daniel, 2016. Systematic review of pertussis immunisation among Asians. International Journal of Human Rights in Healthcare 9, 135–146. https://doi.org/10.1108/IJHRH-02-2016-0002

Pantoja T, Opiyo N, Lewin S, Paulsen E, Ciapponi A, Wiysonge C S, Herrera C A, Rada G, Penaloza B, Dudley L, Gagnon M P, Garcia Marti, S, Oxman A D, 2017. Implementation strategies for health systems in low-income countries: an overview of systematic reviews. Cochrane Database of Systematic Reviews 9, CD011086.

Parrado Jiménez, 2013. Revisión sistemática de la literatura del efecto de pago por desempeño en la calidad de la atención en países de bajos y medianos ingresos.

Pegurri Elisabetta, Fox-Rushby Julia A, Damian Walker, 2005. The effects and costs of expanding the coverage of immunisation services in developing countries: a systematic literature review. Vaccine 23, 1624–1635. https://doi.org/10.1016/j.vaccine.2004.02.029

Rahman A, Fisher J, Bower P, Luchters S, Tran T, Yasamy M T, Saxena S, Waheed W, 2013. Interventions for common perinatal mental disorders in women in low- and middle-income countries: a systematic review and meta-analysis. Bulletin of the World Health Organization 91, 593-601I.

Ranganathan M, Lagarde M, 2012. Promoting healthy behaviours and improving health outcomes in low and middle income countries: A review of the impact of conditional cash transfer programmes. Preventive Medicine 55, S95–S105. https://doi.org/10.1016/j.ypmed.2011.11.015

Ryman, 2008. Too little but not too late: Results of a literature review to improve routine immunization programs in developing countries.

Shea Beverley, Andersson Neil, Henry David, 2009. Increasing the demand for childhood vaccination in developing countries: a systematic review. BMC International Health & Human Rights 9, 1–12. https://doi.org/10.1186/1472-698X-9-S1-S5

Smith B L, Zizzo S, Amzel A, Wiant S, Pezzulo M C, Konopka S, Golin R, Vrazo A C, 2018. Integration of Neonatal and Child Health Interventions with Pediatric HIV Interventions in Global Health. International Journal of MCH & AIDS 7, 192–206.

Watterson Jessica L, Walsh Julia, Madeka Isheeta, 2015. Using mHealth to Improve Usage of Antenatal Care, Postnatal Care, and Immunization: A Systematic Review of the Literature. BioMed Research International 2015, 1–9. https://doi.org/10.1155/2015/153402

Willey, 2013. Effectiveness of interventions to strengthen national health service delivery on coverage, access, quality and equity in the use of health services in low and lower middle income countries.

Wiysonge, 2012. Building evidence for improving childhood immunisation coverage in Africa.

Appendix D: Additional figures

Figure D.1: Impact evaluations by specific intervention category (multi-component separated)

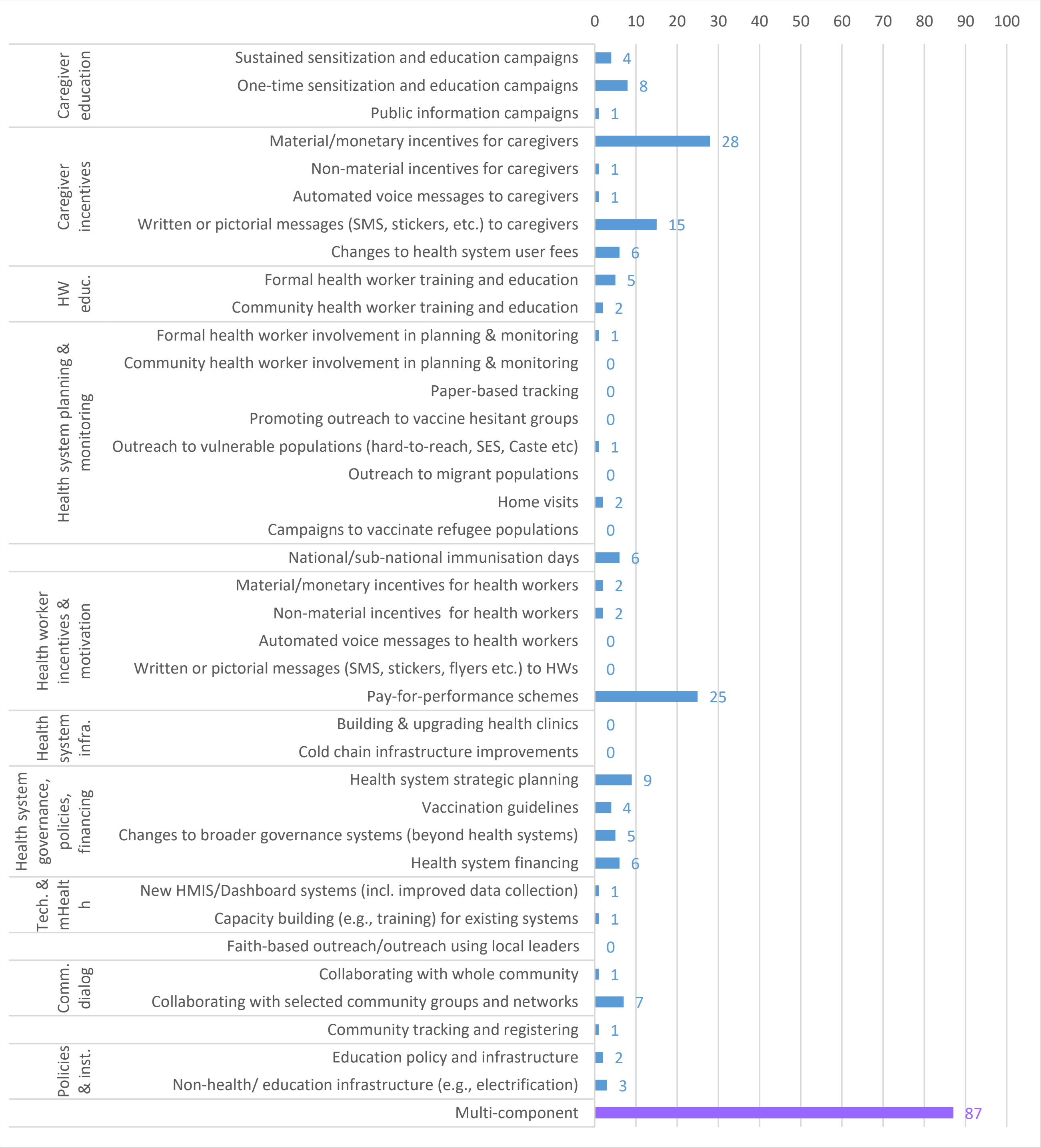

Figure D.2: SR critical appraisals – methods used to identify, include, and critically appraise studies

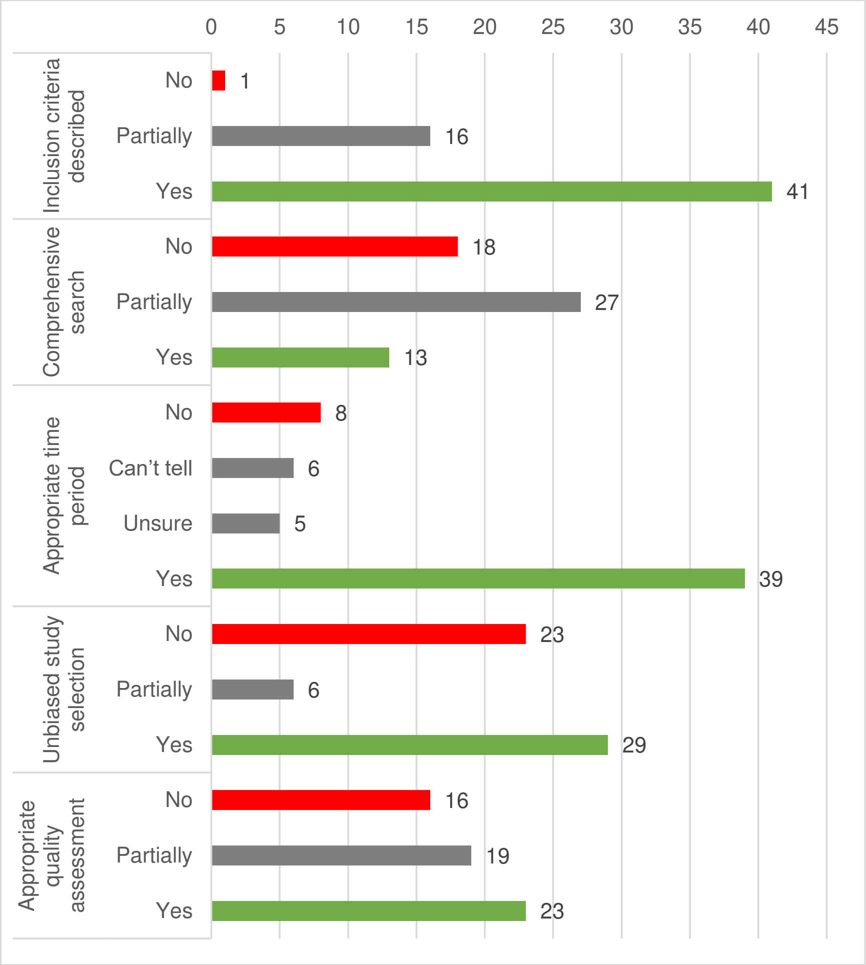

Figure D.3: SR critical appraisals – analysis methods

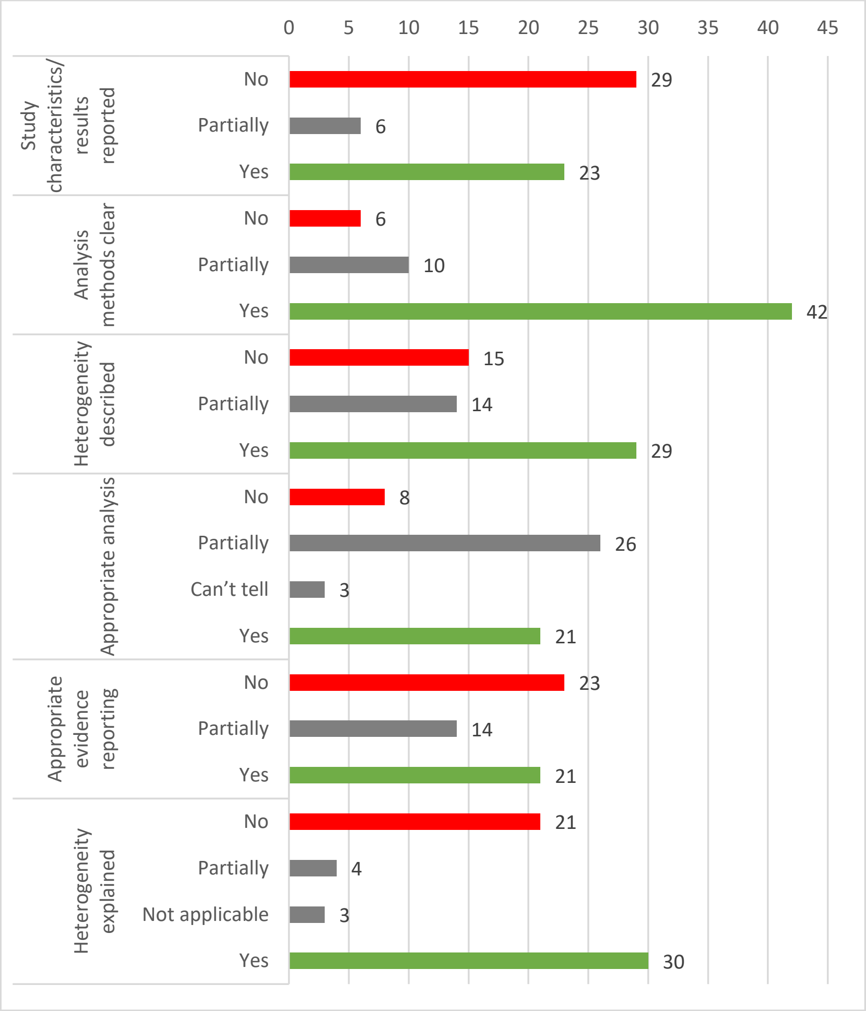

Supplement: Supplementary data [file bmjopen-2021-058258supp001.pdf]
